# Longitudinal characterization of behavioral, morphological and transcriptomic changes in a tauopathy mouse model

**DOI:** 10.18632/aging.205057

**Published:** 2023-11-03

**Authors:** Qing Cao, Manasa Kumar, Allea Frazier, Jamal B. Williams, Shengkai Zhao, Zhen Yan

**Affiliations:** 1 Department of Physiology and Biophysics, State University of New York at Buffalo, Jacobs School of Medicine and Biomedical Sciences, Buffalo, NY 14203, USA

**Keywords:** Alzheimer's disease, tau, cognitive behaviors, transcriptomic, neuronal morphology

## Abstract

Neurodegenerative disorders, such as Alzheimer’s disease (AD), have the gradual onset of neurobiological changes preceding clinical diagnosis by decades. To elucidate how brain dysfunction proceeds in neurodegenerative disorders, we performed longitudinal characterization of behavioral, morphological, and transcriptomic changes in a tauopathy mouse model, P301S transgenic mice. P301S mice exhibited cognitive deficits as early as 3 months old, and deficits in social preference and social cognition at 5–6 months. They had a significant decrease of arborization in basal dendrites of hippocampal pyramidal neurons from 3 months and apical dendrites of PFC pyramidal neurons at 9 months. Transcriptomic analysis of genome-wide changes revealed the enrichment of synaptic gene upregulation at 3 months of age, while most of these synaptic genes were downregulated in PFC and hippocampus of P301S mice at 9 months. These time-dependent changes in gene expression may lead to progressive alterations of neuronal structure and function, resulting in the manifestation of behavioral symptoms in tauopathies.

## INTRODUCTION

Alzheimer’s disease (AD), a multifactorial neurodegenerative disorder characterized by accelerated cognitive decline, is the most common form of dementia [1, 2]. Hyperphosphorylation, misfolding, and aggregation of the microtubule-associated protein tau (MAPT) is one of the pathological hallmarks of AD [3–7]. Moreover, about 80 tau mutations are found in AD-related neurodegenerative disorders [8, 9], such as frontotemporal dementia with parkinsonism linked to chromosome 17 (FTDP-17), corticobasal degeneration (CBD), and progressive supranuclear palsy (PSP). A majority of these missense tau mutations are clustered in the microtubule-assembly domain, causing the reduction of microtubule stability, impairment of axonal transport and dysfunction of synaptic transmission in tauopathies [10–13].

One of the tauopathy models is the PS19 transgenic mice carrying human P301S tau mutation driven by the mouse prion protein promoter. P301S mice exhibit phenotypes resembling early-onset FTDP-17 [14]. Extensive studies of P301S mice further discovered synaptic pathology at 3 months of age, filamentous tau lesions and progressive tau accumulations in PFC, as well as cognitive impairment, at 6 months of age, neuronal loss and hippocampal and entorhinal cortical atrophy by 9–12 months of age [10, 15–19].

Brain regions significantly affected at the early stage of AD include prefrontal cortex (PFC) and hippocampus [20–23]. The most abundant type of neuron in PFC is the pyramidal neuron, each containing a large apical dendrite extending from the soma and ending in a tuft of dendrites and several relatively short basal dendrites. Apical tufts are responsible for cross connections between hemispheres and between regions, while basal dendrites receive inputs from neighboring interneurons and pyramidal neurons [24–26]. Layer V pyramidal neurons serve as the primary output of the PFC. CA1 pyramidal neurons get their primary excitatory inputs at spines on apical and basal dendrites from axon collaterals of CA3 pyramidal neurons, and CA1 pyramidal neurons are the primary output of the hippocampus. Studies in AD patients and AD animal models have found neurite pathology and shaft reductions in pyramidal neurons of PFC (particularly Layer V) and hippocampus (particularly CA1) [27–30]. Here we used the PS19 (P301S) mouse model of tauopathies to characterize longitudinal changes in behavioral, morphological, and transcriptomic levels.

## RESULTS

### Hyperphosphorylated tau is present since the early age in P301S mice

**Figure 1:**
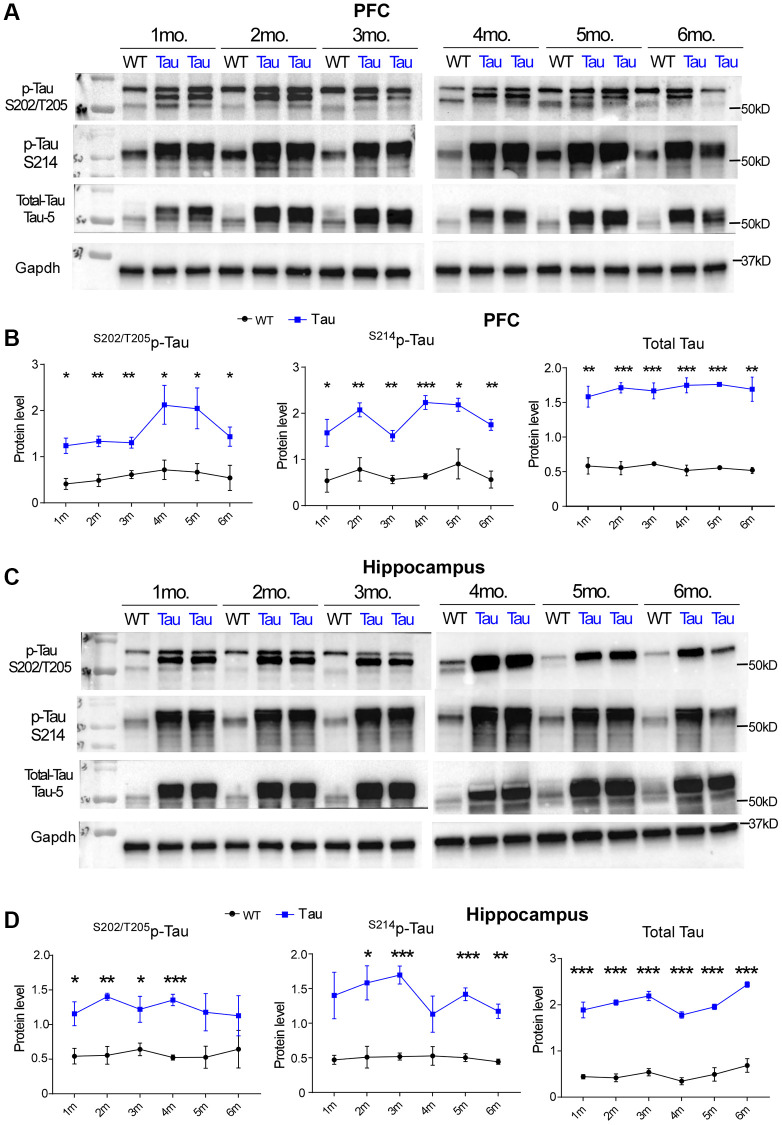
**High expression of hyperphosphorylated tau and total tau was found in P301S Tau mice from early ages.** (**A**, **C**) Representative Western blots showing ^S202/T205^p-tau, ^S214^p-tau and total tau in PFC (**A**) and Hippocampus (**C**) of WT vs. P301S transgenic mice (WT: *n* = 3, P301S: *n* = 4) at 1 month to 6 months of age. (**B**, **D**) Plots of quantification data of ^S202/T205^p-tau, ^S214^p-tau and total tau (normalized to GAPDH) in PFC (**B**) and Hippocampus (**D**) of WT vs. P301S mice at various ages. At each time point, ^*^p < 0.05, ^**^p < 0.01, and ^***^p < 0.001, *t*-test.

Hyperphosphorylation and aggregation of tau are associated with neurodegenerative disorders, and the “pathological” tau phosphorylation sites, such as Ser214, Ser262, Ser202/Thr205 and Ser396/Ser404, are highly relevant to microtubule detachment, cytoskeleton degradation and synaptic failure [11–13]. Phosphorylation at Ser202/Thr205 has been used to classify the staging of AD-related neurofibrillary pathology known as Braak stages [31]. To examine how early tau hyperphosphorylation appears in a tauopathy model expressing P301S mutant tau [16], we measured the level of ^S202/T205^p-tau, ^S214^p-tau and total tau in P301S mice from 1 to 6 months in both PFC and hippocampus regions. Compared to WT, levels of ^S202/T205^p-tau, ^S214^p-tau and total tau were significantly higher in both PFC (Figure 1A, 1B) and hippocampus (Figure 1C, 1D) of P301S mice. This increase was seen from 1 month in both regions and remained consistently increased through 6 months.

### P301S mice exhibit cognitive and social deficits in an age-dependent manner

Cognitive decline is one of the most prominent symptoms of AD. Next, we performed behavioral assays to characterize the longitudinal changes in cognitive processes in P301S mice. One of the cognitive behavioral tests is Barnes maze (BM), a spatial memory task wherein the mouse’s capability of recalling the location of an escape hole out of eight holes with spatial cues is tested. Another cognitive behavioral assay is Novel Object Recognition test (NORT), a short-term memory task wherein the mouse’s capability of distinguishing a novel object from a familiar object is examined. Two-way ANOVA was used to analyze the difference in performance between WT and P301S mice of different ages (factors: Genotype x Age).

**Figure 2:**
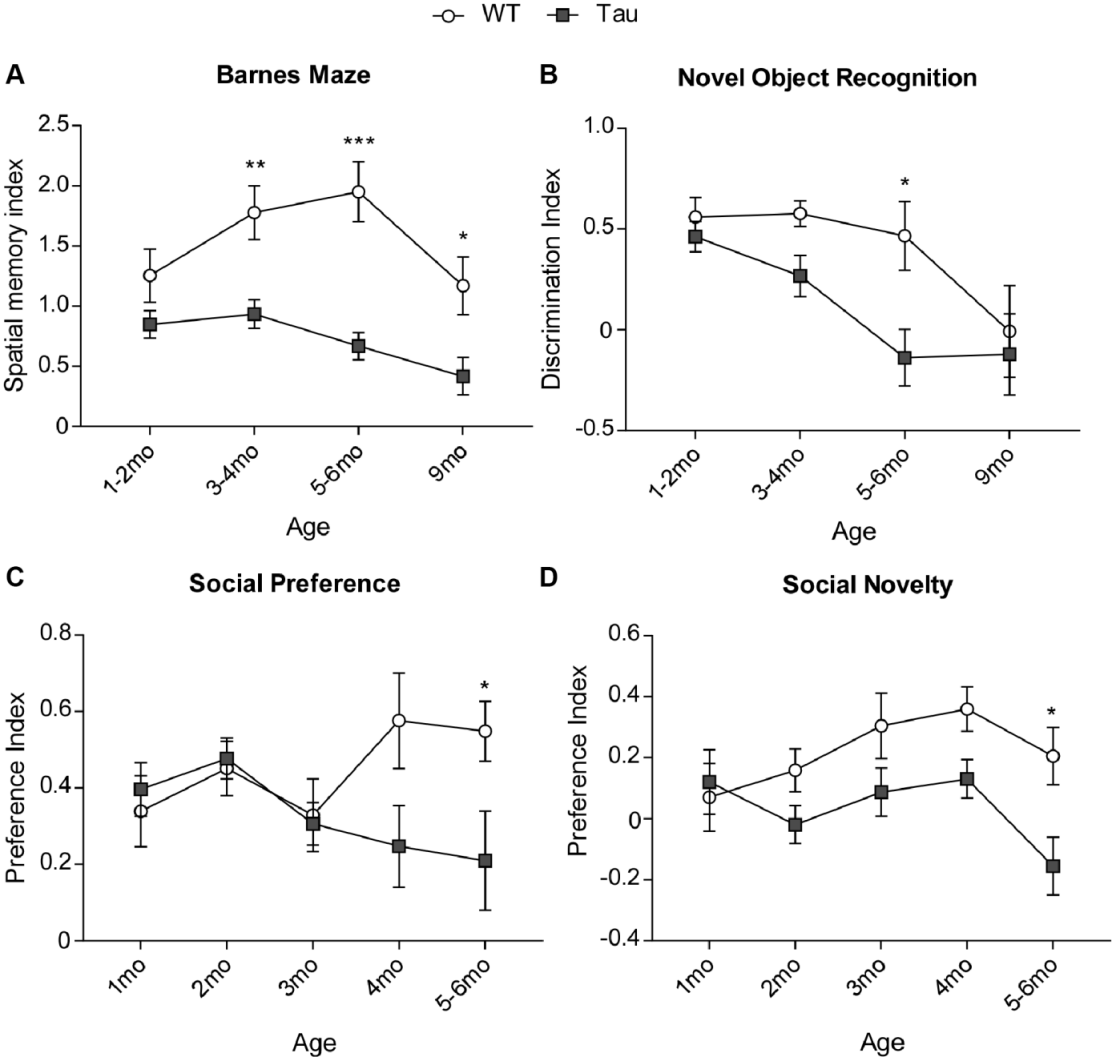
**Behavioral tests showed cognitive and social deficits in P301S Tau mice at early time points.** (**A**) Plots of spatial memory index in Barnes Maze tests of WT vs. P301S transgenic mice at different ages (*n* = WT/P301S: 1–2 m, 14/21; 3–4 m, 15/18; 5–6 m, 15/16, 9 m, 11/10). (**B**) Plots of discrimination index in Novel Object Recognition tests of WT vs. P301S transgenic mice at different ages (*n* = WT/P301S: 1–2 m, 14/21; 3–4 m, 15/17; 5–6 m, 17/9, 9 m, 11/9). (**C**) Plots of social preference index in 3-chamber sociability tests of WT vs. P301S transgenic mice at different ages (*n* = WT/Tau: 1 m, 8/10; 2 m, 13/17; 3 m, 9/13; 4 m, 7/6; 5–6 m, 9/9). (**D**) Plots of social novelty index in social cognition tests of WT vs. P301S transgenic mice at different ages (*n* = WT/Tau: 1 m, 8/10; 2 m, 14/18; 3 m, 9/13; 4 m, 7/6; 5–6 m, 9/9). ^*^p < 0.05, ^**^p < 0.01, and ^***^p < 0.001, two-way ANOVA.

In BM tests (Figure 2A), P301S mice showed a progressive memory impairment (F_3,113 (age)_ = 3.45, *p* < 0.05; F_1,113 (genotype)_ = 38.76, *p* < 0.0001, two-way ANOVA). The spatial memory index (calculated as Time on correct hole (T1)/Time on incorrect holes (T2)) was similar at 1–2 months, but started to show significant deficits in P301S mice at 3–4 months (WT: *n* = 15, P301S: *n* = 18, *p* < 0.01, two-way ANOVA), persisted through 5–6 months (WT: *n* = 17, P301S: *n* = 16, *p* < 0.001, two-way ANOVA), and 9 months old (WT: *n* = 11, P301S: *n* = 10, *p* = 0.06, two-way ANOVA). In NORT tests (Figure 2B), a similar memory decline was found (F_3,105 (age)_ = 6.84, *p* < 0.001; F_1,105 (genotype)_ = 8.36, *p* < 0.01, two-way ANOVA). The discrimination index (calculated as (T_novel_ − T_familiar_)/(T_novel_ + T_familiar_)) showed deficits in P301S mice at 3–4 months and reached significance at 5–6 months of age (WT: *n* = 17, P301S: *n* = 9, *p* < 0.05, two-way ANOVA).

Besides cognitive deficiency, another behavioral alteration of AD is social impairment [32]. Next, we characterized the longitudinal changes in sociability using 3-chamber social preference tests [33] in which the animal’s preference to interact with a social stimulus (an age and gender-matched mouse) vs. a non-social object was tested. Another assay for social cognition is the social novelty test [34], in which the animal’s capability of distinguishing a new social stimulus from an old social stimulus is examined.

In social preference tests (Figure 2C), P301S mice displayed a progressive loss of preference towards the social stimulus against the non-social (ns) stimulus (F_4,91 (interaction)_ = 2.47, *p* = 0.05; F_1,91 (genotype)_ = 4.94, *p* < 0.05, two-way ANOVA). Social preference index (calculated as (T_social_-T_ns_)/(T_social_ + T_ns_)) was lower in P301S mice than WT mice starting at 4 months and reached statistical significance at 5–6 months (WT: *n* = 9, P301S: *n* = 9, *p* < 0.05, two-way ANOVA). In social novelty tests (Figure 2D), P301S mice also displayed a progressive loss of preference towards the new social stimulus against the old social stimulus (F_4,93 (genotype)_ = 10.52, *p* < 0.01, two-way ANOVA). The discrimination index (calculated as (T_new_ − T_old_)/(T_new_ + T_old_)) was lower in P301S mice than WT mice for 2–4 months and reached statistical significance at 5–6 months of age *(*WT: *n* = 9, P301S: *n* = 9, *p* < 0.05, two-way ANOVA).

An AD model, 5xFAD mouse, which carries five familial AD mutations on human amyloid precursor protein (K670N/M671L + I716V + V717I) and human presenilin 1 (M146L + L286V) [35], was also tested to examine the trajectory of behavioral changes. In Barnes maze tests (Supplementary Figure 1A), FAD mice didn’t exhibit memory deficits until 5–6 months old (*n* = 6–7/group/age, F_2,31 (interaction)_ = 4.86, *p* < 0.05; F_1,31 (genotype)_ = 4.2, *p* < 0.05, two-way ANOVA). No significant change was found in 5xFAD mice in social preference (Supplementary Figure 1B) and social novelty (Supplementary Figure 1C) tests.

Taken together, these data indicate that the tauopathy model exhibit significant cognitive and social deficits at 5–6 months. For some more challenging cognitive tasks, such as BM, the significant deficits are manifested at even earlier time points (3–4 months). Thus, P301S mice is a better model to capture early changes in various behavioral aspects of AD.

### Pyramidal neurons in PFC and hippocampus of P301S mice exhibit altered dendritic morphology at different ages

To examine the longitudinal changes in neuronal structure, which may be associated with the alteration of behavioral function, we performed Golgi-Cox staining of pyramidal neurons in PFC and hippocampus of P301S mice at early (3-month) and late (9-month) time points. Neurons were traced and reconstructed from z-stack images using Neuromantic application. Axon was excluded from reconstruction as it would interfere with the analysis of dendrites. Number of branches were evaluated using Sholl analysis and compared using two-way ANOVA (factors: Genotype x Sholl Radius).

**Figure 3:**
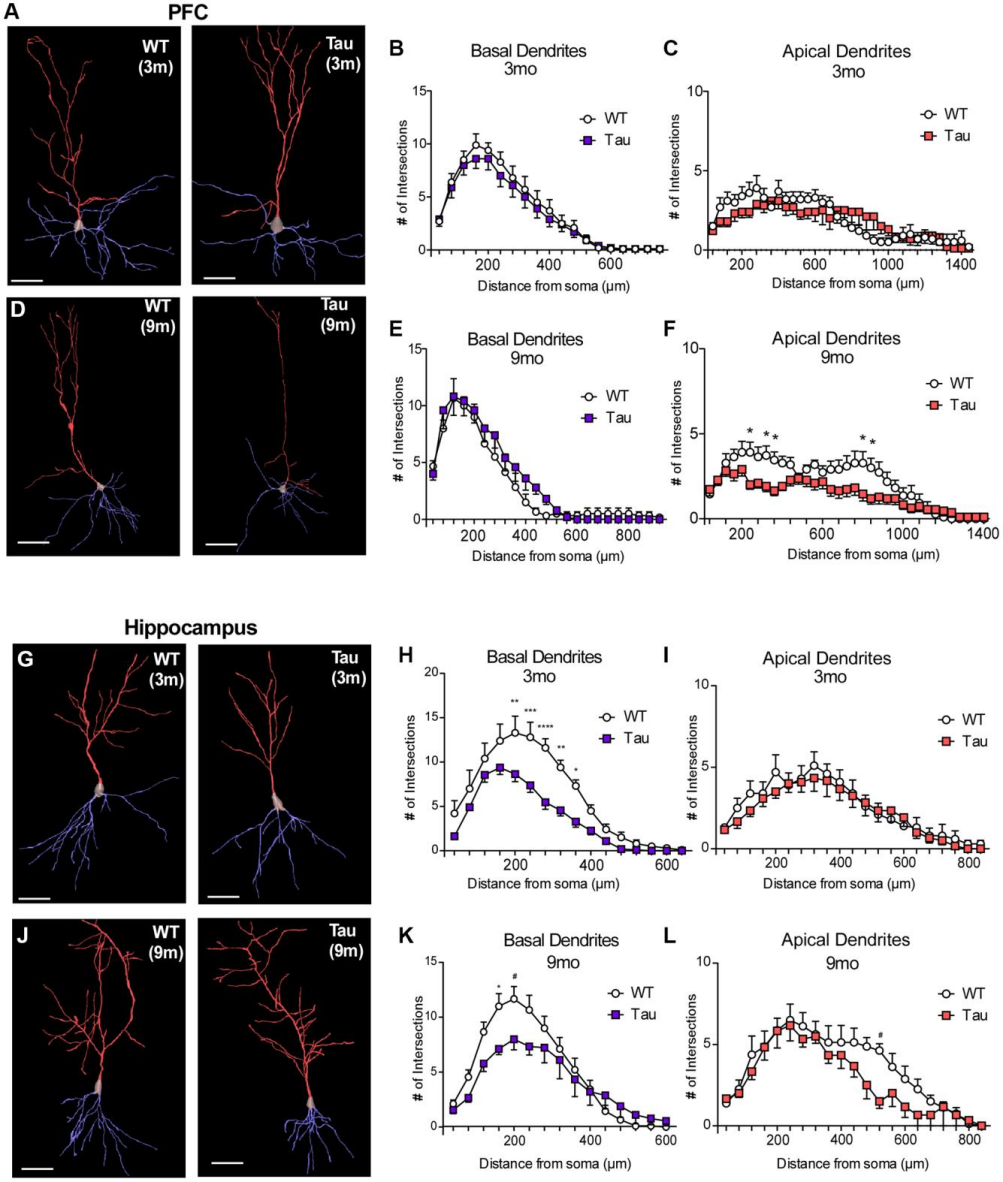
**Golgi staining revealed morphological changes in pyramidal neurons of PFC and hippocampus from P301S Tau mice at different ages.** (**A**, **D**, **G**, **J**) Representative reconstructed images of layer V pyramidal neurons in PFC (**A**, **D**) or CA1 hippocampus (**G**, **J**) from WT and P301S mice at 3 months (**A**, **G**) and 9 months (**D**, **J**). Basal dendrites are traced in blue, while apical dendrites are traced in red. Scale = 100 μm. (**B**, **C**, **E**, **F**) Plots of dendritic branching as measured by Sholl analysis of basal dendrites and apical dendrites of layer V PFC pyramidal neurons from WT and P301S mice at 3 months (**B**, **C**, *n* = 10 neurons/group) and 9 months (**E**, **F**, *n* = 11 neurons/group). (**H**, **I**, **K**, **L**) Plots of dendritic branching as measured by Sholl analysis of basal dendrites and apical dendrites of CA1 pyramidal neurons from WT and P301S mice at 3 months (**H**, **I**, *n* = 10 neurons/group) and 9 months (**K**, **L**, *n* = 10 neurons/group). ^*^p < 0.05, ^**^p < 0.01, ^***^p < 0.001, and ^****^p < 0.0001, two-way ANOVA.

Representative traced PFC pyramidal neurons of WT vs. P301S mice at 3 months and 9 months are shown in Figure 3A, 3D. Z-project images of these neurons are shown in Supplementary Figure 2A, 2C. At 3 months old (Figure 3B, 3C), no changes in basal (*n* = 10 neurons per group, F_1,342_ = 2.49, *p* = 0.12) or apical (F_1,648_ = 1.49, *p* = 0.22) dendrite arborization were observed. At 9 months of age (Figure 3E, 3F), basal dendrites showed no significant changes *(n* = 11 neurons per group, F_1,460_ = 2.02, *p* = 0.16). However, Sholl analysis of apical dendrites revealed a significant (F_1,700_ = 61.98, *p* < 0.0001) reduction of the number of projections from proximal (240 μm, *p* = 0.02; 320 μm, *p* = 0.04; 360 μm, *p* = 0.04) and distal (800 μm, *p* = 0.04; 840 μm, *p* = 0.04) stems of apical dendrites in PFC pyramidal neurons from P301S mice.

Representative traced hippocampal CA1 pyramidal neurons of WT vs. P301S mice at 3 months and 9 months are shown in Figure 3G, 3J. Z-project images of these neurons are shown in Supplementary Figure 2B, 2D. At 3 months (Figure 3H, 3I), Sholl analysis for arborization of basal dendrites revealed a significant reduction in CA1 pyramidal neurons of P301S mice (*n* = 10 neurons per group, F_1,304_ = 65.62, *p* < 0.0001; 200 μm, *p* = 0.0045; 240 μm, *p* = 0.0004; 280 μm, *p* < 0.0001; 320 μm, *p* = 0.0026; 360 μm, *p* = 0.027), while apical dendrites did not show any changes (F_1,420_ = 2.15, *p* = 0.14). At 9 months (Figure 3K, 3L), Sholl analysis revealed a significant reduction in the number of branches from basal dendrites (*n* = 10 neurons per group, F_1,240_ = 9.17, *p* = 0.0027; 160 μm, *p* = 0.045; 200 μm, *p* = 0.077), as well as a decrease in projections from distal apical stems (F_1,252_ = 12.43, *p* = 0.0005; 520 μm, *p* = 0.05).

### Transcriptomic analysis reveals longitudinal alterations of gene expression in the hippocampus of P301S mice

To find out the molecular basis that might underlie the longitudinal changes in behavioral phenotypes and neuronal morphology, we analyzed the RNAseq data from hippocampus of WT vs. P301S (PS19) mice at various ages [36]. Using a cutoff of P_adj_ < 0.05 and Fold change |(FC)|>1.2, we identified differentially expressed genes (DEGs) at the late stage (9 and 12 months) of P301S tau mice (283 upregulated, 206 downregulated). The 283 late stage upregulated genes (Supplementary Figure 3A–3D) are enriched in microglia cell activation and related phagocytosis process. The protein-protein interaction network demonstrated hub genes including complement system (C1qa, C1qb). These genes had a low expression at the early stage of P301S tau mice, and gradually increased at the older age. The 206 late stage downregulated genes (Supplementary Figure 3E–3H) are enriched in synaptic transmission and action potential-related pathway, and many hub genes are ion channels (Scn2b, Kcnip2). These genes had a high expression at the early stage, and gradually decreased at the older stage. This pattern is consistent with transcriptomic changes in neurodegenerative diseases by other studies [37–39].

**Figure 4:**
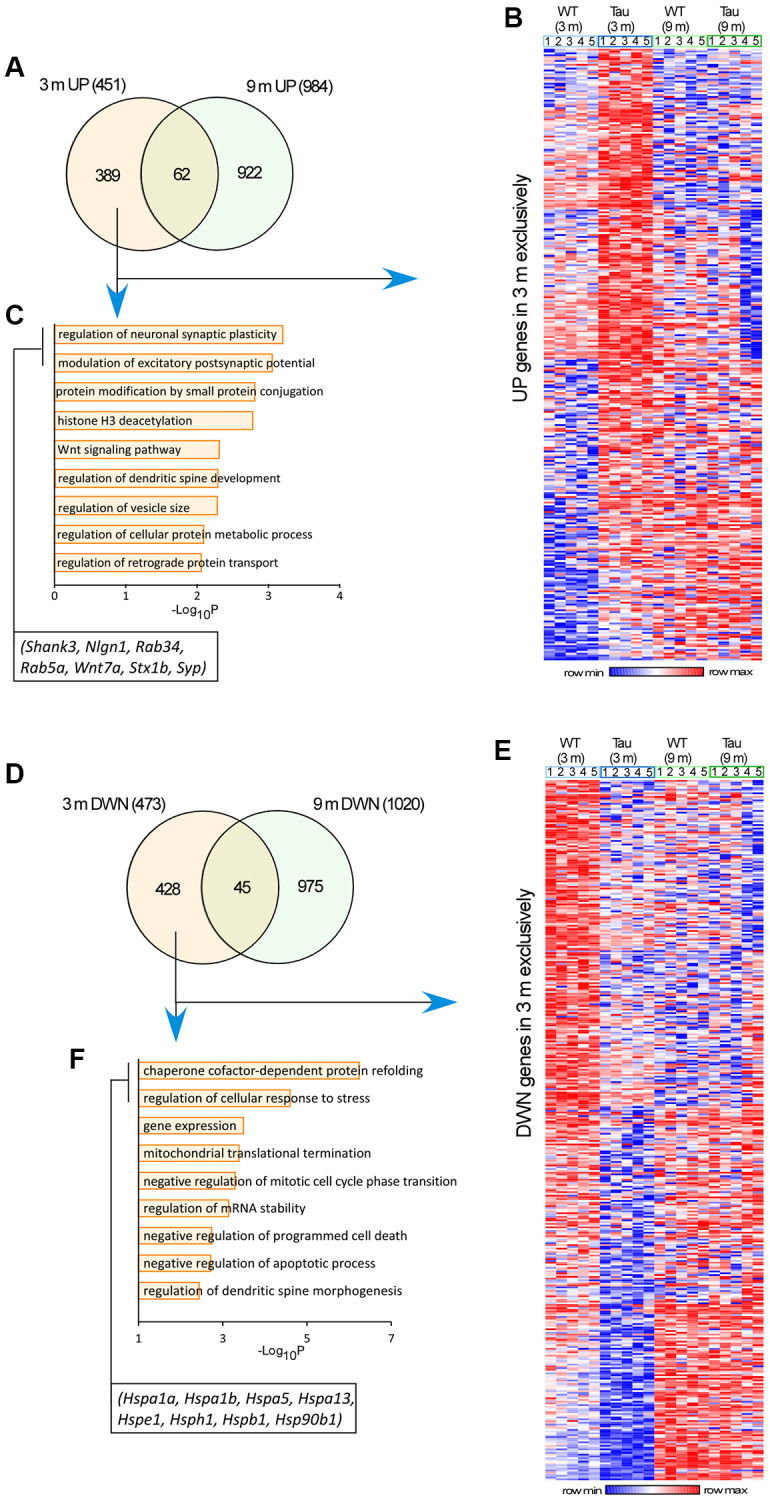
**Transcriptomic analysis revealed genome-wide alterations in the hippocampus of P301S mice at different ages.** (**A**, **D**) Venn-diagrams showing the significantly up-regulated (**A**) or down-regulated (**D**) genes in P301S mice at 3 months and 9 months. (**B**, **E**) Heatmaps representing expression (row z-score) of genes that were up-regulated (**B**) or down-regulated (**E**) in P301S mice exclusively at 3 months, compared to age-matched WT samples (*n* = 5 mice/group). (**C**, **F**) GO Biological Process analysis of up-regulated genes (**C**) or down-regulated genes (**F**) in P301S mice exclusively at 3 months.

Next, we searched for gene changes that occur at the early stage. Among the 451 upregulated genes in the hippocampus of 3-month-old P301S mice, 389 genes were exclusively increased at 3 months, compared to 3-month-old WT mice (Figure 4A, 4B and Supplementary Table 1). GO Biological Process (BP) analyses of these upregulated genes indicated that the most prominently enriched pathways included the regulation of neuronal synaptic plasticity, modulation of excitatory postsynaptic potential, *Wnt* signaling, and regulation of spine development. Key genes in the top two GO pathways included *Nlgn1* (encoding Neuroligin-1), *Shank3, Stx1b* (encoding Syntaxin 1B), *Syp* (encoding Synaptophysin), *Rab34,* and *Rab5a* (Figure 4C), all of which are involved in synaptic organization and synaptic vesicle trafficking.

Among the 473 downregulated genes in the hippocampus of 3-month-old P301S mice, 428 genes were exclusively decreased at 3 months (Figure 4D, 4E, and Supplementary Table 2). GO BP analyses of these downregulated genes indicated that the most prominently enriched pathways included the regulation of protein folding, cellular response to stress, gene expression, and mitochondrial translational termination. Key genes in the top two GO pathways included *Hspa1a, Hspa1B, Hspa5, Hspa13, Hspe1, Hsph1, Hspb1,* and *Hsp90b1* (Figure 4F), all of which encode the heat shock protein family members that act as chaperones to regulate protein assembly and ER homeostasis.

### Gene expression is altered in the PFC and hippocampus of P301S mice at different ages

**Figure 5:**
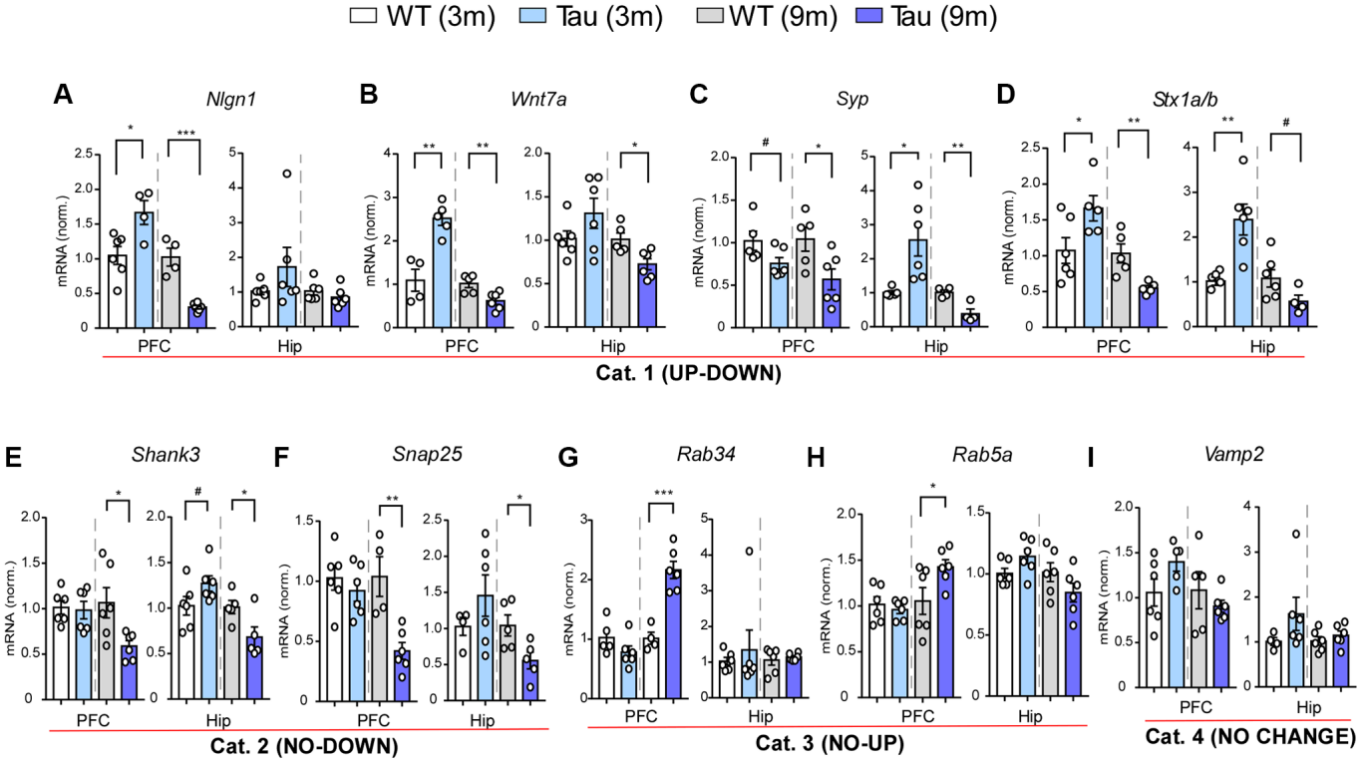
**Gene expression profiling revealed various patterns of changes in PFC and hippocampus of P301S mice at different ages.** (**A**–**I**) Bar graphs of mRNA levels in PFC and hippocampus of WT and P301S mice at 3 and 9 months (n: WT/Tau = 6/6 per group), which included: Category 1 (UP-DOWN) (**A**–**D**, *Nlgn1, Wnt7a, Syp, Stx1b*); Category 2 (NO-DOWN) (**E**, **F**, *Shank3*, *Snap25*); Category 3 (NO-UP) (**G**, **H**, *Rab34, Rab5a*); and Category 4 (NO CHANGE) (**I**, *Vamp2*). ^#^*p* < 0.1, ^*^p < 0.05, ^**^p < 0.01, ^***^p < 0.001, *t*-test.

To validate the microarray data, we next performed quantitative PCR (qPCR) to examine the expression of selected genes in PFC and hippocampus of WT vs. P301S mice at 3 months and 9 months (*n* = 6/group). We first focused on synaptic genes, many of which were found to be exclusively upregulated in hippocampus of P301S mice at 3 months by the transcriptomic analysis. We revealed 4 patterns of gene alterations. Category 1 (UP-DOWN) genes showed the elevated expression at 3 months and the diminished expression at 9 months in both regions of P301S mice, including the genes involved in synaptic transmission and plasticity like *Nlgn1, Wnt7a, Syp,* and *Stx1a/b* (Figure 5A–5D), which is largely consistent with the microarray data. Category 2 (NO-DOWN) genes showed no change at 3 months in either PFC or hippocampus, but a significant downregulation at 9 months in both regions, which included *Shank3* (encoding a postsynaptic scaffolding protein regulating dendritic spine formation and synapse maintenance) and *Snap25* (encoding a SNARE complex protein regulating transmitter release) (Figure 5E, 5F). Category 3 (NO-UP) genes showed no change at 3 months and a significant upregulation at 9 months in PFC, while remained unchanged in hippocampus, which included *Rab34* and *Rab5a* (encoding Ras superfamily protein members involved in endocytosis) (Figure 5G, 5H). Category 4 (NO CHANGE) included *Vamp2,* which showed no change at 3 months or 9 months in PFC or hippocampus (Figure 5I).

We further examined a few heat shock protein genes, which were found to be exclusively downregulated in hippocampus of P301S mice at 3 months by the transcriptomic analysis. Surprisingly, we found either no change or a significant increase in most of these genes in PFC or hippocampus of 3-month-old P301S mice (*n* = 6), compared to 3-month-old WT mice (*n* = 6) (Supplementary Figure 4). The discrepancy could be due to small sample sizes or different methodological sensitivities.

While it is difficult to point out which category (UP-DOWN, NO-DOWN, NO-UP) would be the key players, we speculate that the loss of synaptic proteins resulting from less transcription (UP-DOWN, NO-DOWN) and/or more endocytosis (NO-UP) is responsible for the synaptic deficits in aged group.

Given the dynamic alteration of synaptic genes in P301S mice, we finally performed Western Blotting to detect protein changes at different time points. As shown in Supplementary Figure 5, the synaptic proteins NLGN1, STX1A, VAMP2, SYP, and SHANK3 were largely unchanged or showed a trend of reduction in total lysates of PFC and hippocampus from P301S mice at 3 months and 9 months, compared to age-matched WT mice (*n* = 4/group).

## DISCUSSION

In this study, we have used P301S tauopathy model to characterize longitudinal changes in various behaviors, neuronal morphologies and gene expression. The new information regarding the abnormalities in different domains and trajectories of phenotypical manifestations could provide insights into the discovery of causal mechanisms for AD.

Tau is overexpressed from birth in P301S mice, and we tested whether hyperphosphorylated tau had age-related and region-specific changes. We found that P301S mice expressed constantly high levels of phosphorylated and total tau in both PFC and hippocampus since the early age, which is slightly different from a previous study showing that synaptic tau protein levels were significantly increased in the hippocampus, but reduced in the PFC, of P301S mice in response to age [40]. The conformational change in mutant tau renders it to be proaggregation, and aggregated tau can induce synaptic decay and neuronal loss [41, 42]. Furthermore, hyperphosphorylation of tau induces tau missorting from axons to the somatodendritic compartment, which can cause synaptic dysfunction [11].

Many prior studies have assessed the appearance of tangles in P301S mice. Neurofibrillary tangles (NFTs) were found in neocortex, amygdala, hippocampus, brainstem and spinal cord of P301S mice at 6 months old [16]. The properties of tan tangles were also measured in hippocampus of aged P301S mice (14 months old) [43]. In the study of autophagy homeostasis of misfolded protein diseases [44], P301S mice (2–3 months old) were stained with MC1 (an antibody raised to paired helical filaments that are the predominant component of tangles). In a recent study of tau seeding and spreading mechanisms [45], tau oligomers and tau fibrils (two main precursors of NFTs) were examined in 6-month-old P301S mice.

Our behavioral assays of P301S mice have revealed significant deficits in spatial memory by Barnes maze at 3 months, and deficits in recognition memory at 5–6 months, consistent with prior findings on cognition and memory impairment of these mice at an early time-point (~3 months) [16, 18]. In agreement with this, spatial memory deficits by Morris Water maze were found to be the earliest phenotype in AD models [46], while recognition deficits manifested later [47], similar to the symptomatic progression of AD patients. In addition, we have uncovered the significant deficits in social preference and social cognition behaviors in P301S mice at 5–6 months. These data suggest that the P301S tauopathy model produces robust changes in the brain even preceding the formation of neurofibrillary tangles, which is enough to produce observable behavioral phenotypes at an early stage.

One notable finding is that older WT mice (9 months) show the worse performance in the Novel Object Recognition task, compared to younger WT mice. This ageing-induced cognitive impairment may result from working memory deficits mediated by PFC and episodic memory deficits mediated by hippocampus [48]. Aging could cause subtle synaptic alterations without inducing neuron loss [48–50].

Our morphological studies have revealed a significant reduction of the arborization of basal dendrites in CA1 pyramidal neurons of P301S mice at 3 months, which persisted till 9 months. It is consistent with the finding that hippocampus is one of the first regions in the human brain to be affected by AD [51]. It is also corroborative of previous studies [52], which reported a reduction of overall dendritic length in CA1 pyramidal neurons of the P301L tauopathy mouse model. For layer V PFC pyramidal neurons, we found a significant reduction in the number of secondary and tertiary branches extending from the proximal and distal stems of the apical dendrite in P301S mice at 9 months. This loss of dendritic arborization will lead to the reduced inputs from apical tufts, resulting in diminished output signals from PFC in P301S mice.

Other than dendritic arborizations, another morphological change could be in dendritic spines. A previous study has reported a significant reduction of dendritic spine density in hippocampal pyramidal neurons of P301S mice from young adulthood onwards [53]. However, another study has found unchanged spine density in the hippocampus and medial PFC of PS19 mice at 6 and 9 months [40].

Our transcriptomic analysis has revealed that the upregulated genes in hippocampus of P301S mice at 3 months are enriched in synaptic plasticity. The expression of these synaptic genes, including *NLGN1, SYP, WNT7A,* and *STX1B*, was found to be significantly increased in PFC and hippocampus of P301S mice at 3 months and decreased at 9 months by qPCR assays. It is consistent with a prior transcriptomic study of PFC from humans with different stages of AD [20], which found that genes regulating synaptic function and ATP synthesis (865 genes) were upregulated during the early pre-symptomatic stage of AD (Braak 0–III), and downregulated at later stages that coincide with the appearance of pathological features (amyloid plaques and tau tangles) and cognitive impairment (Braak IV–VI).

The temporally orchestrated increase of synaptic genes in P301S mice suggests that synaptic activity is increased at the early stage, which may represent a coping mechanism against the increasing cell stress from pathological burdens. In agreement with this, increased neuronal activity was observed in humans during preclinical or early stages of AD, which was found to parallel with the increased expression of genes involved in synaptic transmission and plasticity [54]. Patients at risk for AD carrying the presenilin I (*PSEN I*) mutation showed higher activation of the hippocampus and frontal/temporal cortices during associative memory encoding years before clinical symptoms manifested [55–57]. Several APP transgenic mouse models, such as TgCRND8 and 3xTg-AD (which contains mutant human tau P301L), had increased hippocampal synaptic plasticity, which was associated with episodic memory deficits [58, 59]. A recent study investigating the V337M mutation of *MAPT* in a cerebral organoid model reported the upregulation of synaptic genes enriched in glutamatergic signaling pathways in 2-month mutant neurons preceding cell death [60]. The upregulation of glutamatergic signaling, combined with other changes, such as increase in autophagy-lysosomal pathway markers and splicing changes, was found to produce an increase in vulnerability to excitotoxicity.

There are several limitations to our findings. First, qPCR experiments have validated the mRNA changes of some, but not all, genes found in transcriptome analysis. The different results from RNA-seq and qPCR could be due to the relatively small sample sizes, small fold changes, distinct samples, and different sensitivities, which results in the limited power of significance with each approach. Because of the modest change in mRNA levels, we did not get the same protein changes in some groups, since Western Blotting is much less sensitive than qPCR. Furthermore, many of the detected proteins are enriched at synapses, and are not well represented in total protein lysates, which may cause the lack of significant changes in PS19 mice. Second, despite the observed changes in dendritic architecture and synaptic genes, we have not included longitudinal functional studies on the impact of tau mutation on synaptic transmission. Our previous electrophysiological studies of PFC pyramidal neurons found significant deficits of AMPAR- and NMDAR-mediated synaptic currents in 6-month-old P301S mice [10, 19, 61]. Future studies will determine whether the age-dependent up- and down-regulation of synaptic signaling pathways has an impact on synaptic function or intrinsic neuronal properties in PFC and hippocampus.

In conclusion, our longitudinal characterization of behavioral, morphological and transcriptomic changes in a tauopathy mouse model is to elucidate potential mechanisms that drive the progression of AD and related neurodegenerative disorders. Manipulation of key molecular players coupled with electrophysiological measurements of neuronal functions in future studies will help identify early intervention strategies for these diseases.

## MATERIALS AND METHODS

### Animals

All experiments were performed with the approval of the State University of New York at Buffalo Animal Care Committee. The PS19 mouse line harboring the T34 isoform of microtubule-associated protein tau (MAPT) with one N-terminal insert and four microtubule binding repeats (1N4R) encoding the human P301S mutation [16] was obtained from the Jackson laboratory. The genetic background was (C57BL/6 × C3H) F1, and the breeding system was noncarrier × hemizygote. Genotyping was performed by PCR of tail DNA according to the manufacturer’s protocol. Both male and female P301S mice (1–9 months) and age-matched WT littermates were used. Since no sex-dependent effects were found in our measured parameters, data from both males and females were pooled together.

### Behavioral testing

Animals were habituated to the experimental room in their home cages for at least 30 minutes before testing. The room light was adjusted to dim during all behavioral experiments. Mice were returned to the home cages between trials to rest. To mask olfactory cues, all testing apparatuses were cleaned with 75% ethanol. Operators were blind to experimental groups during testing and scoring. ANY-maze 5.1 (Stoelting) was used for animal tracking and data analysis. To avoid potential problems of repeated measurements, each animal was only tested once for each behavioral assay. At each age group, different mice were used.

#### Barnes maze

As described before [62, 63], the mouse was placed on a round platform with eight equally spaced holes at the edge, one of which was attached with an escape box (correct hole). Bright overhead light was applied as a weak aversive stimulation to increase the motivation to escape from the circular platform. During the three learning phases (5-min interval) (information acquisition), the mouse could explore the platform using distal visual cues until finding the correct hole and entering the escape box. Then, the mouse was placed in its home cage to rest for 15 min. In the memory phase (information retention and retrieval), the escape box was removed, and the mouse was put back on the platform to explore for 5 min. In this phase, the time spent on the correct hole (T1) and the other seven incorrect holes (T2) were counted. Spatial memory index was calculated as T1/T2.

#### Novel object recognition test

The animal was first put on a round platform with no objects for habituation (5 min), then two identical objects were placed on the platform for the animal to explore (5 min). In the test phase, a novel object and a familiar object from the last phase were placed on the platform for the animal to explore (5 min). The mouse was removed from the arena and placed in its holding cage for 5 minutes between phases. All objects were made of plastic toys (height, about 5 cm) with similar textures, colors, and sizes but distinctive shapes. The objects were positioned in two adjacent corners (10 cm from the walls) counterbalanced. The arena and objects were cleaned between each trial with 70% alcohol to mask any olfactory cues. Exploration was defined by directing the nose at ≤2 cm to the object and/or touching it with the nose. Exploration time of the familiar and novel objects was recorded and used to calculate a discrimination index (time on novel object − time on familiar object)/total time on both.

#### Social preference and novelty test

As described before [33, 34, 64], the test mouse was first placed into a 3-chamber Plexiglass arena (L: 101.6 cm, W: 50.8 cm, H: 50.8 cm) containing two empty inverted pencil cups on the side chambers for 10-min habituation. On the following day, the mouse was reintroduced to the apparatus for a 10-min trial in which the pencil cups contained two identical non-social objects. The animal was returned to its home cage for 5 minutes. Then it was placed into the apparatus for a 10-min trial (social preference test), in which one cup contained a novel non-social object and the other contained a social stimulus (an age- and sex-matched WT mouse). Five minutes later, the animal was tested in a final 10-min trial, in which one cup contained a novel social stimulus (a new age- and sex-matched WT mouse) and the other contained an old social stimulus (same mouse from the prior phase). The amount of time spent interacting with each stimulus was recorded. The social preference index was calculated as (time on social stimulus – time on non-social object)/total time on both. The social novelty index was calculated as (time on new social stimulus – time on old social stimulus)/total time on both.

### Golgi-cox staining and Sholl analysis

Neurons were visualized using FD Rapid GolgiStain™ Kit (PK401A, FD Neurotechnologies, Inc.). Mouse brains were perfused with 1X PBS. After 15 days of stain impregnation, brains were sliced (100 μm) using a vibratome and mounted onto gelatin-coated slides (PO101, FD Neurotechnologies, Inc.). Allowing the slides to dry for 48 hours, slices were treated with blackening solution, dehydrated through graded ethanol, cleared with xylene, and cover-slipped with DPX mounting medium. For analysis, 4 to 8 neurons were selected per region per animal. Pyramidal neurons from the infralimbic and prelimbic regions of the PFC and the CA1 region of the hippocampus were selected. A neuron was selected if at least two intact primary basal dendrites, along with intact distal apical stem(s), were attached to the soma. Z-stack images were taken with 10× (1.6× magnification) or 20× (1× magnification) objectives using Leica DMi8 inverted microscope for bright-field imaging and Leica LAS X software for image acquisition. Neurons were semi-automatically traced and reconstructed using Neuromantic application v1.6.3. Sholl analysis was performed by uploading the reconstruction file (.swc) into ImageJ Sholl Analysis Plugin part of SNT, v4.1.12 [65]. Analyses for basal and apical dendrites were done separately at 40 μm intervals.

Both apical and basal dendrites of 84 (total) pyramidal neurons in 8 groups from two different regions (PFC and CA1) in two different genotypes (WT and P301S) of mice at two different ages (3 months and 9 months) were reconstructed and analyzed. The technical limitation of Golgi staining precludes the possibility of getting a large number of neurons with completely preserved basal and apical dendritic arborizations from each animal.

### Bioinformatic analysis

RNA-seq dataset (GSE89979) for gene expression in hippocampus of PS19 Tau Transgenic mice at 3 and 9 months [36] was acquired using NCBI’s public database gene expression omnibus (GEO) and used for Differentially Expressed Gene (DEG) analysis, as what we previously described [39]. Genes with differential expression of adjusted *p*-values below 0.05 and fold change (FC) above 1.2 were considered as being significantly different. Gene Ontology (GO) analyses and functional classification was done using Enrichr. The *p*-value for each category and the combined score generated by Enrichr were used to identify top biological process enrichment for each dataset.

### Quantitative real-time PCR

Total RNA was isolated with the TRIzol reagent (Invitrogen, USA), and the genomic DNA was removed by incubating with RNase-free DNase I (Invitrogen, USA). Purified mRNA was then converted to cDNA with an iScript reverse transcription kit (Bio-Rad, USA). Quantitative real-time PCR was performed on the iCycler iQ Real-Time PCR Detection System and iQ Supermix (Bio-Rad, USA) according to the manufacturer’s instructions. The average value of two replicates of each sample was expressed as the threshold cycle (Ct), at which the fluorescence signal reaches 10X the SD of the baseline. Then, the difference (ΔCt) between the Ct value for target gene and the Ct value for housekeeping gene GAPDH (ΔCt = Ct(target gene) – Ct(GAPDH)) was calculated for each sample. The relative level of target gene expression was determined by fold change (FC) = 2^−ΔΔCt^, where ΔΔCt = ΔCt – mean of ΔCt (control group). Primers used are:

**Table:** 

**Gene**	**Name**	**Primers**
*Nlgn1*	Neuroligin 1	f: CGAGCACTGGGGATTCATCT
		r: ATTCACCCACGGACACTTCC
*Stx1a/b*	Syntaxin 1A/B	f: CGTGGAGAGCCAGACTATGT
		r: CTGGAGTGGAGTGGCAGTTT
*Wnt7a*	Wnt Family Member 7A	f: CTCTTCGGTGGTAGCTCTGG
		r: TATGACGATGATGGCGTCCG
*Syp*	Synaptophysin	f: GTCAGTTCCGGGTGGTCAAG
		r: AAGTACACTTGGTGCAGCCT
*Shank3*	SH3 and ankyrin repeat domains 3	f: GATCTGCCATCCCTACAAC
		r: AGCTAAGGGTGAGCTAGGAT
*Rab34*	Ras-related protein Rab-34	f: ATTTCCGGGGATCGTGTTGC
		r: GGGATGGCCCTACCATAACAG
*Rab5A*	Ras-related protein Rab-5A	f: CAGGGTGAGAAGAAGGAGCGAG
		r: AAATTACCTGGGCCGCGT
*Snap25*	Synaptosome Associated Protein, 25kDa	f: TCCCGAGAAGCCCAGGTAAG
		r: GCAGCTCACCTCGAAAACAC
*Vamp2*	Vesicle Associated Membrane Protein 2	f: GTCTCTCCTGCGTTCCCC
		r: CGACCTCACAGATGCGATCC
*Hspa1a*	Heat Shock Protein Family A (HSP70) Member 1A	f: TATGTGGCCTTGAGGACTGT
		r: ACAAATCACATCAGCGGGGC
*Hspa1b*	Heat Shock Protein Family A (HSP70) Member 1B	f: TGCTTGGGCACCGATTACTG
		r: AGTGCTGCTCCCAACATTAC
*Hspa5*	Heat Shock Protein Family A (HSP70) Member 5	f: GAGTCTGCTTCGTGTCTCCT
		r: GCAGTCAGGCAGGAGTCTTAG
*Hspa13*	Heat Shock Protein Family A (HSP70) Member 13	f: CACGAGCGATGTCTGGAAAC
		r: CTGGAAGGGAGAAAGCCGTA
*Hspb1*	Heat Shock Protein Family B (Small) Member 1	f: ATCACTGGCAAGCACGAAGA
		r: GGCCTCGAAAGTAACCGGAA
*Hspe1*	Heat Shock Protein Family E (Hsp10) Member 1	f: TTTTCACGTGTCCCAGCCG
		r: GTTTCGGCAGCACTCCTTTC
*Hsph1*	Heat Shock Protein Family H (Hsp110) Member 1	f: AGGCTACATAAGGCTGAGCG
		r: ATGTAGCAGCTCTGTGAGCC
*Hsp90b1*	Heat Shock Protein 90 Beta Family Member 1	f: GCTCCTGAGACCGAAAAGGA
		r: GCCTTCTCGGCTTTTACCCA
*GAPDH*	Glyceraldehyde-3-phosphate dehydrogenase	f: GACAACTCACTCAAGATTGTCAG
		r: ATGGCATGGACTGTGGTCATGAG

### Western blotting

Mice were sacrificed by decapitation, and brains were quickly removed and cooled in ice-cold PBS. Next, brains were placed and sliced in a 1 mm interval mouse brain matrix (Zivic Instruments, USA). Brain sections were selected and 2 punches from PFC and whole hippocampus were taken. The tissue was homogenized in 0.2 μM filtered 1% SDS supplemented with protease inhibitor (Complete, Roche, USA). Protein concentration was quantified with Bradford protocol (Bio-Rad, USA). Samples were boiled in 4X SDS loading buffer for 5 min and the total protein extracts (10 μg) were separated on 6% or 12% SDS gels.

Nitrocellulose membranes were incubated overnight at 4°C with the following primary antibodies: tau (Tau-5) (1:500, Thermo Fisher Scientific, AHB0042), Ser202/Thr205 phospho-tau (AT8) (1:500; Thermo Fisher Scientific, MN1020), Ser214 phospho-tau (1:1000; Thermo Fisher Scientific, 44-742G), Nlgn1 (1:5000, Proteintech, 66964-1-Ig), Stx1a (1:500, Proteintech, 66437-1-Ig), Syp (1:5000, BD Biosciences, 611880), Shank3 (1:500, NeuroMab, 367-62), Vamp2 (1:500, Proteintech, 10135-1-AP), glyceraldehyde-3-phosphate dehydrogenase (GAPDH) (1:1000, Cell Signaling, 5174). All proteins were detected by using an anti-mouse (1:2000, GE Lifesciences, NA931) or anti-rabbit (1:2000, GE Lifesciences, NA934) secondary antibody IgG coupled to peroxidase and developed by ECL (SuperSignal West-Pico, Thermo Fisher). Images and data analysis were acquired with Chemidoc XRS system (Bio-Rad).

### Statistical analyses

Data were analyzed with GraphPad Prism v.7 (GraphPad). Differences between two groups were assessed with unpaired Student’s *t*-test with unequal variance. Experiments with more than two groups were subjected to two-way ANOVA with Bonferroni correction for multiple *post hoc* comparisons. Data points identified as statistically significant outliers (as determined by Grubb’s test) were removed from the analyses. All values are mean ± SEM.

## Supplementary Materials

Supplementary Figures

Supplementary Tables
