# Supplementary material for: Longitudinal characterization of behavioral, morphological and transcriptomic changes in a tauopathy mouse model
Source: Aging (Albany NY). 2023 Nov 3;15(21):11697–719. doi: 10.18632/aging.205057 (PMC10683589; doi:10.18632/aging.205057)
Supplement: Supplementary Figures [file aging-15-205057-s001.pdf]

SUPPLEMENTARY FIGURES

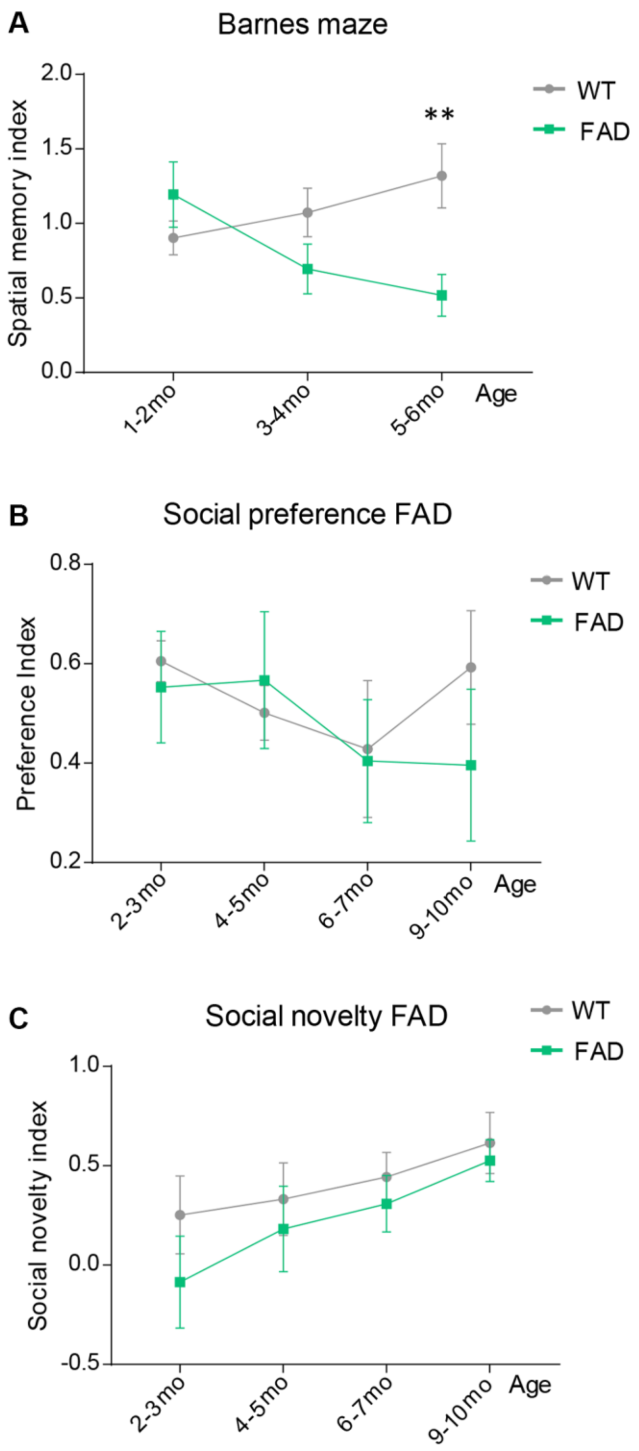

Supplementary Figure 1. Behavioral tests of 5XFAD mice at various ages (**A**–**C**). Plot of spatial memory index in Barnes maze tests, social preference index and novel social discrimination index in sociability tests of WT vs. FAD mice from 1–2 months old to 9–10 months old. (*n* = 5–7/group/age) $^{*}p < 0.05$, two-way ANOVA.

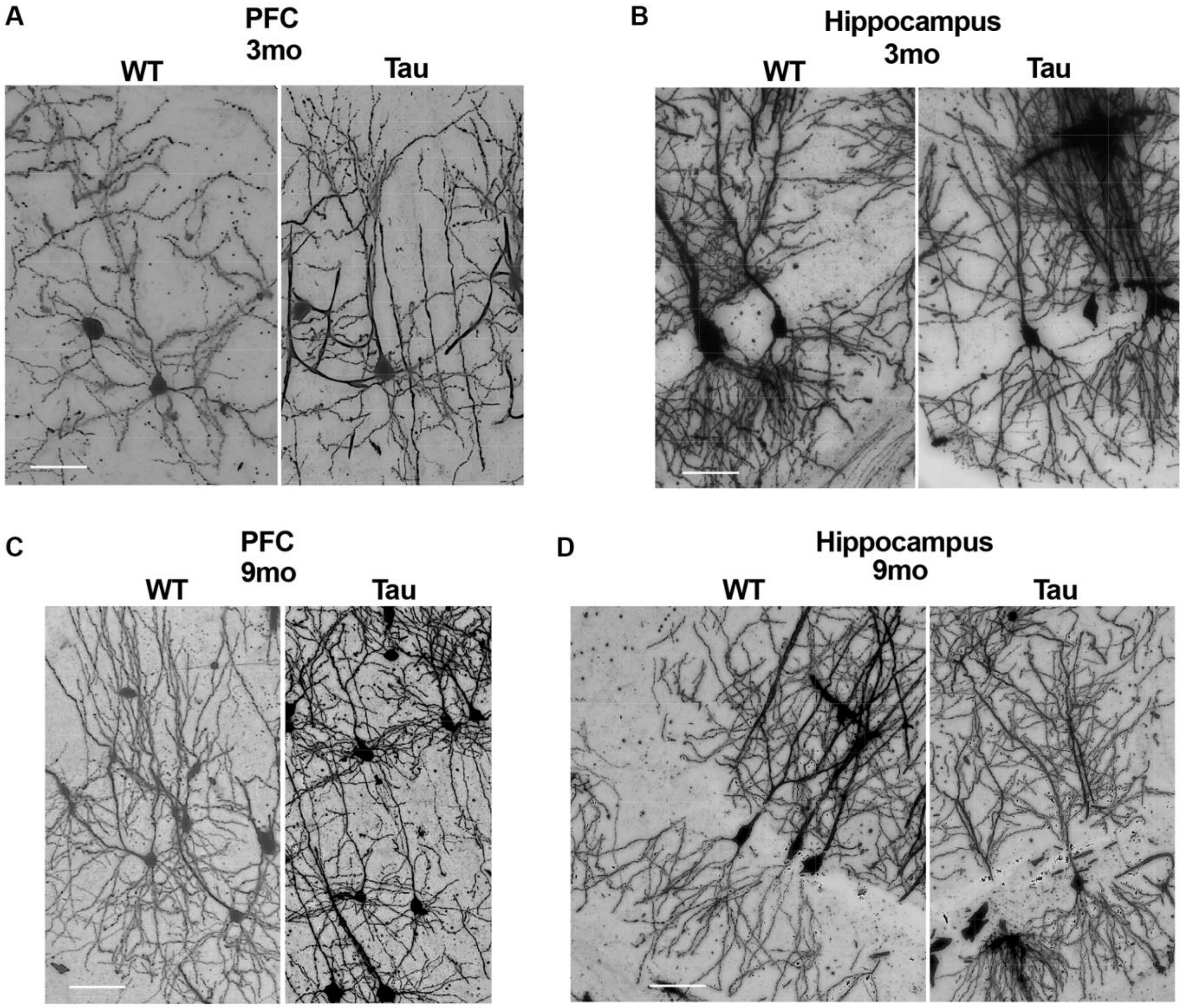

Supplementary Figure 2. Images of Golgi-stained neurons shown in Figure 4. (**A**, **C**) Images (z-projected) of layer V PFC pyramidal neurons at 3 months and 9 months of WT and P301S mice. (**B**, **D**) Images (z-projected) of CA1 hippocampus pyramidal neurons at 3 months and 9 months of WT and P301S mice. Scale = 100 mm.

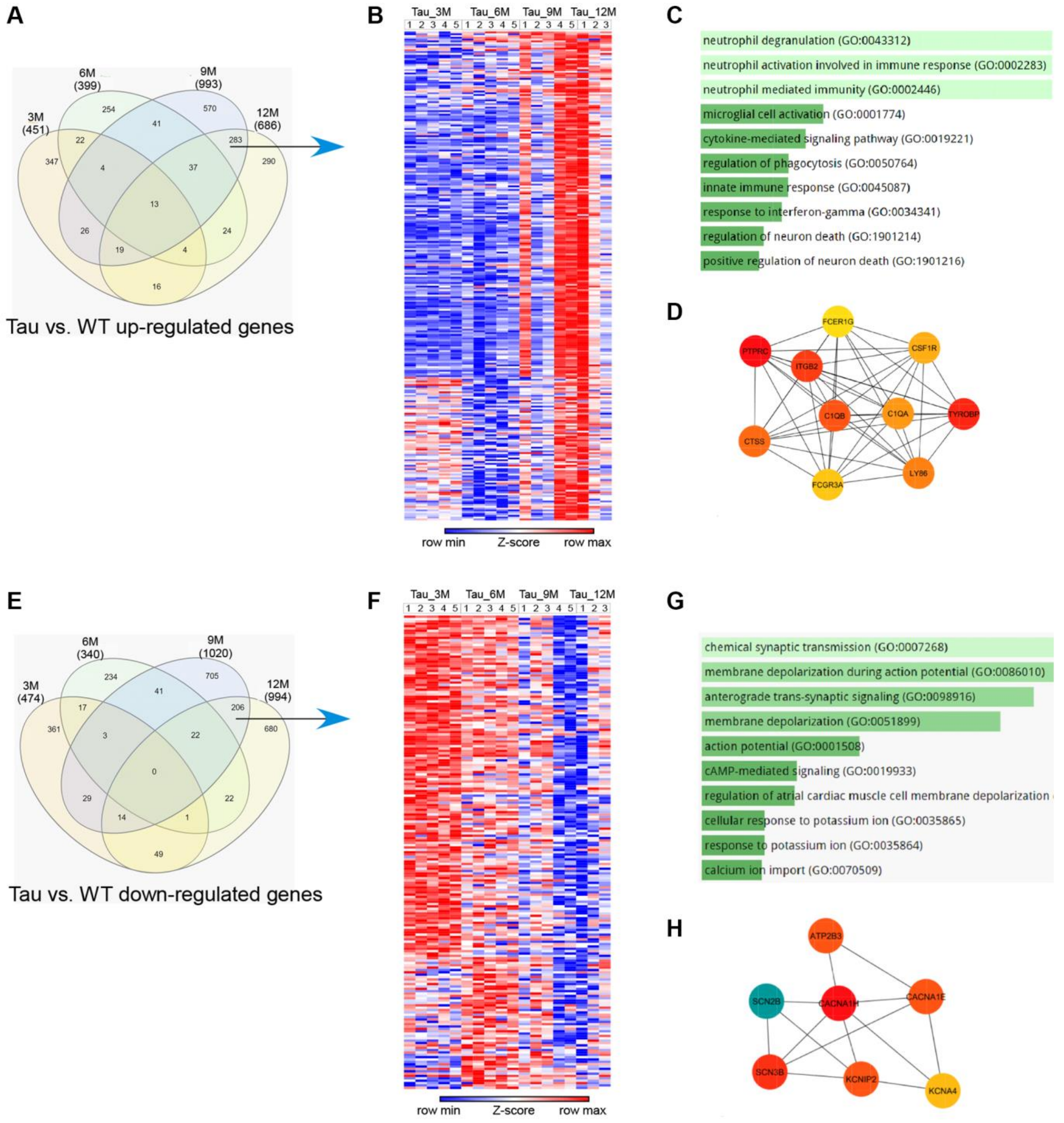

Supplementary Figure 3. Progressive transcriptomic changes in hippocampus of PS19 (P301S) mice. (**A**, **E**) Venn diagram showing the overlapping of up-regulated or down-regulated genes in PS19 mice at various ages. (**B**, **F**) Heatmap representing expression (row z-score) of up-regulated or down-regulated genes at the late stage (9 and 12 months). (**C**, **G**) GO Biological Process analysis of the up- or down-regulated genes at the late stage. (**D**, **H**) Hub genes detected from protein-protein interactions of up- or down-regulated genes at the late stage.

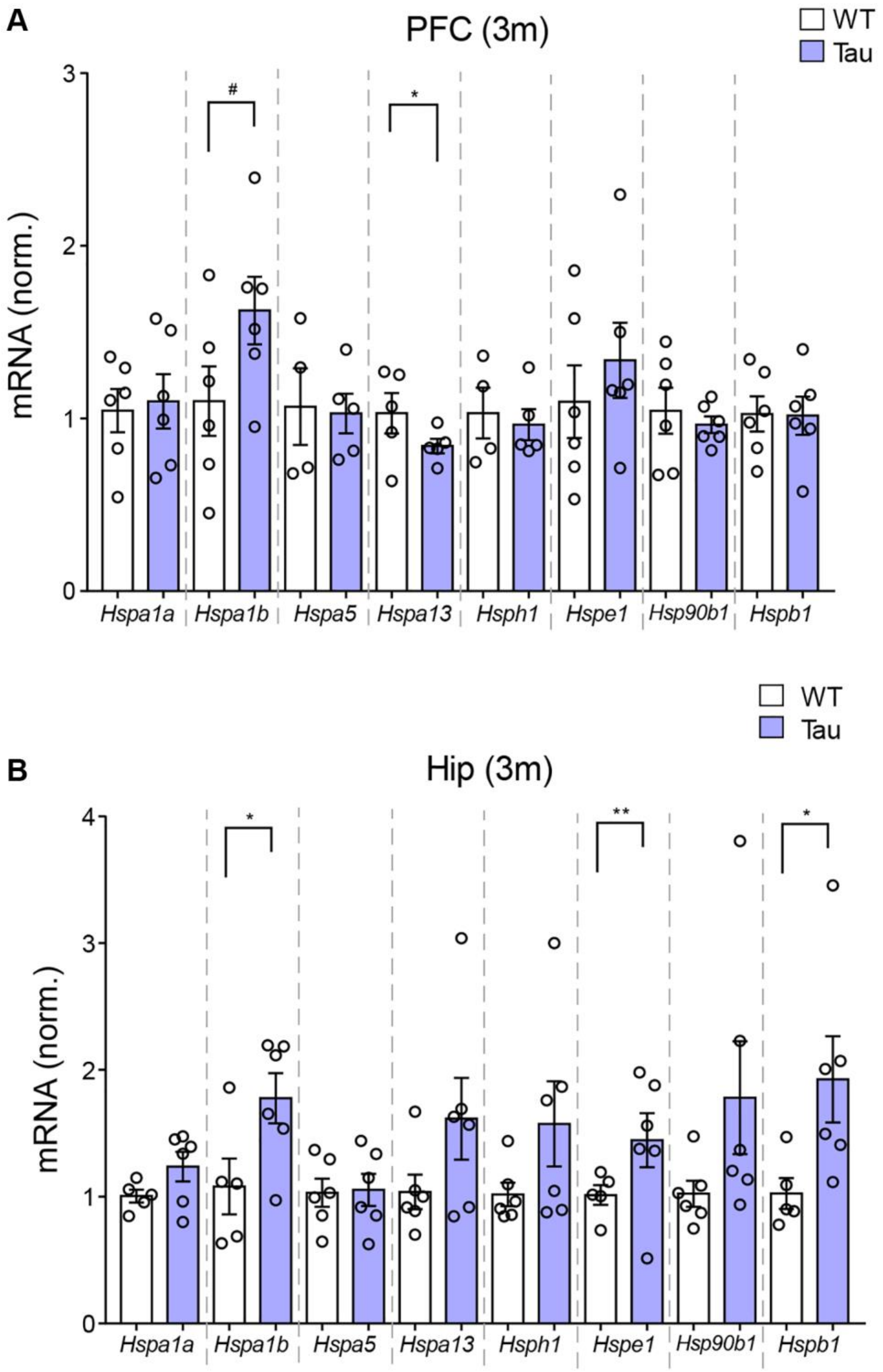

Supplementary Figure 4. Expression of genes encoding heat-shock proteins by qPCR assays. (**A**, **B**) Bar graphs of mRNA levels in PFC (**A**) and hippocampus (**B**) of 3-month-old WT vs. P301S mice (n = 6 pairs). $^{\#}p < 0.1$, $^{*}p < 0.05$, $^{**}p < 0.01$, t-test.

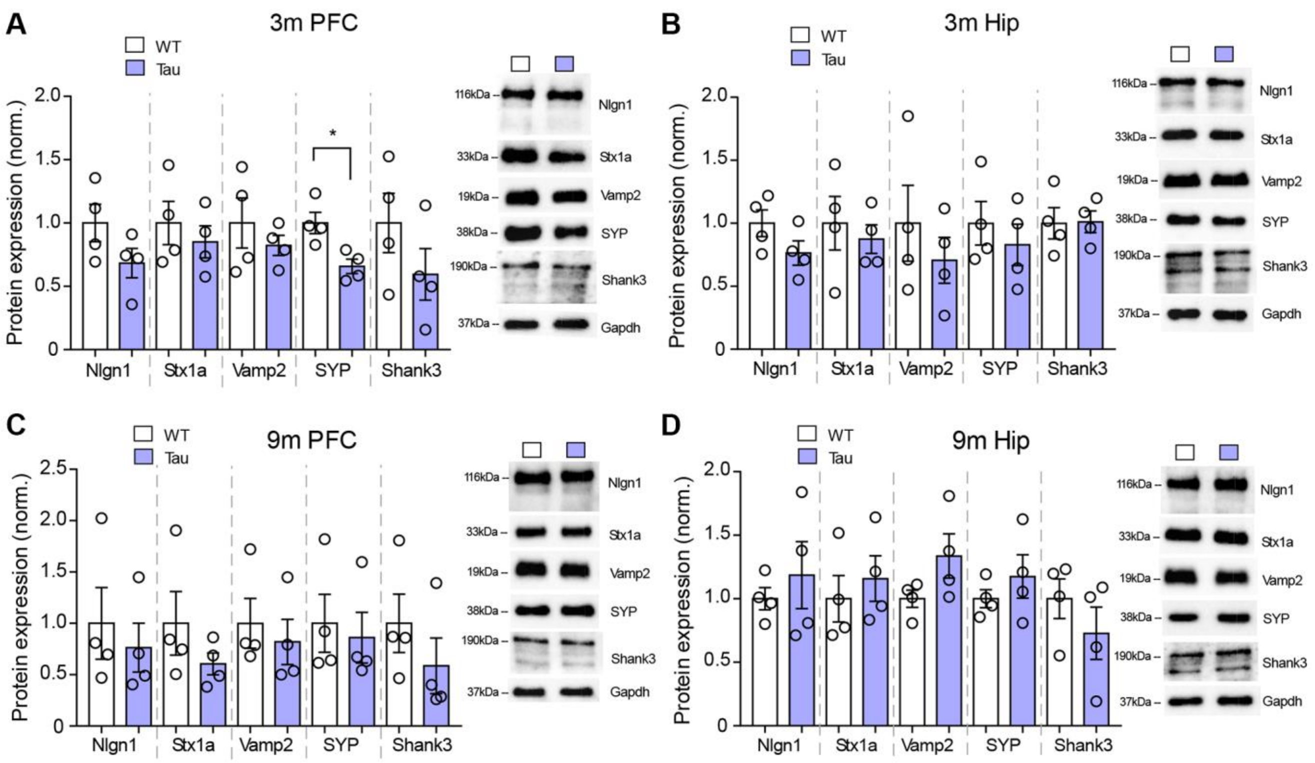

Supplementary Figure 5. Expression of synaptic proteins in total lysates of PFC and hippocampus by Western blotting. (**A**–**D**) Bar graphs showing the protein level of Nlgn1, Stx1a, Vamp2, SYP, Shank3 in PFC and hippocampus of 3-month-old (**A**, **B**) or 9-month-old (**C**, **D**) WT vs. P301S mice (*n* = 4 mice/group). Representative blots are also shown. $^{*}p < 0.05$, *t*-test.
