# Supplementary material for: Longitudinal characterization of behavioral, morphological and transcriptomic changes in a tauopathy mouse model
Source: Aging (Albany NY). 2023 Nov 3;15(21):11697–719. doi: 10.18632/aging.205057 (PMC10683589; doi:10.18632/aging.205057)
Supplement: Supplementary Tables [file aging-15-205057-s002.pdf]

Supplementary Table 1. List of genes exclusively upregulated in the hippocampus of P301S mice at 3 months, compared to 3-month-old WT mice.

Gene symbol	WT_1_3M	WT_2_3M	WT_3_3M	WT_4_3M	WT_5_3M	Tau_1_3M	Tau_2_3M	Tau_3_3M	Tau_4_3M	Tau_5_3M	WT_1_9M	WT_2_9M	WT_3_9M	WT_4_9M	WT_5_9M	Tau_1_9M	Tau_2_9M	Tau_3_9M	Tau_4_9M	Tau_5_9M
Slc35a1	-1.91	-0.47	-0.05	0.80	1.17	1.71	1.26	2.12	1.39	1.52	-0.55	-1.23	0.25	0.59	-0.01	-1.45	0.26	0.55	0.11	-0.21
Zfp131	-1.78	-0.04	-0.06	0.36	0.06	0.96	2.53	0.28	1.00	1.46	0.63	-1.03	0.92	-0.89	-0.68	0.03	0.63	-0.93	-0.07	0.30
LOC552904	0.31	-1.10	-1.67	-0.34	-0.29	1.34	1.10	-0.30	1.93	0.50	-0.76	-1.59	-0.72	-0.15	0.13	-0.40	-0.31	1.16	-0.01	-0.50
AI195381	-0.92	-1.04	0.17	0.19	-0.14	1.86	0.72	0.18	1.84	1.16	0.01	-0.24	-0.36	-0.10	-1.15	0.06	0.64	-0.17	-0.62	0.17
Stx1b	0.10	0.16	0.52	-0.14	-2.52	1.49	1.08	0.45	1.63	0.92	-0.28	-1.79	0.25	-0.45	-1.78	0.14	-0.10	0.20	-1.12	-0.92
Derl2	0.73	0.97	-1.02	0.53	-0.32	2.27	2.35	1.04	1.10	1.51	-1.53	0.04	0.19	-0.13	-0.46	-0.14	-0.03	0.44	-0.11	-0.01
Dcp2	0.17	-2.57	-1.28	0.21	0.88	0.56	0.98	0.43	1.79	0.93	1.26	-0.91	-0.32	-2.71	-1.39	0.13	0.34	-1.10	0.99	0.47
Ap4s1	0.17	-0.28	-0.05	-0.34	-0.81	0.48	1.27	1.63	0.95	1.22	-2.11	0.02	-0.79	-1.42	0.01	-0.32	-0.15	-0.98	1.44	0.90
Dalrd3	-0.72	-1.08	1.63	2.34	1.73	-0.02	2.46	-0.69	-0.42	1.64	-0.08	0.07	-0.13	0.28	-1.26	-2.02	-0.49	-0.73	-0.42	-0.37
Rnf24	0.61	-0.54	0.44	0.14	0.40	2.12	0.25	1.44	0.85	2.83	-1.04	0.24	-0.15	-0.53	0.26	-1.51	0.40	0.42	-0.30	0.22
Tmem186	-0.29	-0.30	-2.21	-1.36	-0.47	-0.19	1.13	-0.74	0.64	0.57	-1.75	-1.12	0.05	-1.92	-0.17	0.06	-1.52	0.61	-0.64	0.13
Mex3a	-1.61	-0.27	-0.08	0.51	0.76	1.55	1.44	1.65	0.29	0.34	-0.35	-0.45	-0.71	-0.62	-1.49	-0.22	0.47	0.29	-0.09	-0.40
Carkd	0.55	-1.02	-0.12	0.87	-0.40	1.37	-0.55	1.69	0.92	2.40	-0.71	0.50	0.07	1.25	-0.22	0.66	-1.12	-0.76	-0.27	-0.01
Tiam1	-0.75	-0.80	-0.50	-0.16	0.89	1.58	0.01	1.20	0.97	0.85	-0.55	-0.99	0.30	-0.91	0.93	-1.91	0.69	-0.93	-0.63	-0.24
Picalm	0.60	-0.38	0.19	-0.53	0.12	2.19	0.39	0.39	1.70	1.17	-0.64	-0.42	-2.11	-1.02	0.63	0.55	1.05	-0.55	-1.36	0.75
BC022960	-0.36	-0.11	0.14	0.90	-0.51	0.80	0.48	0.53	1.74	2.27	1.51	-0.59	1.37	-0.62	0.64	0.48	0.26	-0.13	1.84	-0.08
Purg	1.33	-0.49	0.12	0.22	0.36	1.68	0.21	0.93	1.93	2.51	0.61	0.00	0.43	0.64	-1.16	-0.64	-1.01	0.64	0.10	-0.24
Brinp3	0.06	0.71	-0.50	0.19	0.31	0.55	2.13	1.66	1.18	0.94	1.25	-0.73	-1.55	-1.69	-0.86	1.02	-0.64	-0.45	-0.05	0.46
Rpia	-0.46	-0.64	-0.01	0.25	0.49	0.49	1.61	1.11	1.31	0.79	-0.31	0.04	0.50	-0.32	0.24	0.87	-0.33	-0.07	-0.63	-0.01
Mrps25	-0.25	-1.19	-0.03	-0.17	0.50	0.73	0.88	0.62	0.52	1.69	0.83	-0.45	0.12	-0.13	0.74	-0.55	0.85	0.02	0.58	-0.20
Cirbp	-1.13	1.12	0.01	0.05	-0.10	1.84	1.29	1.75	0.61	0.02	-0.05	0.33	0.80	-1.02	0.03	0.21	0.13	1.17	-0.02	0.31
Zranb2	0.44	-1.02	0.41	0.46	0.32	1.56	0.09	0.16	2.60	1.70	-0.48	-0.48	0.75	-0.61	0.51	-0.31	0.20	0.19	-0.45	-0.14
Acbd6	-0.12	1.08	-0.50	-0.94	0.17	0.90	1.93	1.05	0.66	0.60	-2.10	-0.52	0.44	-0.87	1.28	-0.16	0.80	-1.00	-1.03	-0.44
Clpp	-0.92	0.45	1.01	0.70	-0.09	1.03	1.08	2.23	1.26	0.98	0.48	-0.58	-0.54	-0.47	0.24	-0.64	-1.65	0.10	-0.57	0.55
Smim19	-0.20	-0.55	-0.07	0.78	-0.08	1.02	1.83	0.67	1.54	0.24	1.26	0.07	0.43	-1.21	-0.95	0.40	-0.98	-0.79	-0.40	0.75
BC038331	0.03	-0.60	0.53	0.38	-0.06	-0.45	1.46	1.51	1.70	1.47	-0.71	2.14	0.27	-0.53	-0.62	-1.03	1.48	-0.91	-1.14	0.93
Ppt2	0.87	0.79	-0.11	0.08	-0.03	1.27	2.37	1.04	1.32	0.91	0.77	-1.03	-1.01	-0.86	-0.88	1.78	-0.55	0.28	-0.52	-0.86
Ppip5k2	-1.56	0.04	0.45	0.86	0.45	0.75	1.24	2.21	1.05	0.30	-0.86	-0.31	1.70	0.05	-0.29	-0.18	-0.23	-0.16	1.19	-0.07
Sdc3	-0.22	0.61	-0.05	0.88	0.24	1.83	1.14	0.25	2.12	1.38	0.27	1.05	-0.50	-0.62	-1.08	0.25	0.49	-1.09	-1.26	0.29
Tmem88b	-1.15	0.63	0.65	0.82	-0.74	1.04	0.81	0.94	1.01	1.65	-0.59	-0.47	0.03	-0.41	-1.49	-0.23	-0.68	0.61	0.68	1.04
Sssca1	-0.78	0.88	-0.51	0.76	0.07	1.30	0.66	0.65	1.58	1.44	0.12	-0.95	0.27	-0.50	0.63	0.21	1.17	1.34	0.10	0.29
Dnaic1	0.20	-1.08	0.12	0.84	-0.57	1.18	0.82	0.80	0.64	1.29	-1.31	-0.75	1.56	0.68	-1.44	-0.43	0.23	0.60	0.44	0.42
Efs	0.08	0.95	-0.63	0.09	-0.32	1.21	0.79	1.11	1.29	0.95	-0.75	1.16	-1.35	-1.63	-0.20	-2.17	0.24	-0.05	-0.17	-0.56
AU044157	0.51	0.28	-1.54	-0.63	0.10	0.28	0.71	1.00	1.17	0.70	-2.67	-0.15	-0.01	0.26	0.38	-0.16	0.01	-0.46	0.01	-0.75
Gfra2	0.11	0.20	-0.45	0.70	-1.09	1.21	0.70	0.90	0.59	1.20	0.78	-1.81	0.64	-0.59	-1.86	-1.30	-2.63	-1.01	-0.89	0.28
Slc35e2	0.08	-1.53	0.32	0.30	0.52	1.12	0.92	1.02	1.17	0.54	-0.49	-2.37	-0.09	-0.66	-0.39	-0.99	0.22	0.64	0.04	0.26
Clca3a1	-0.60	1.22	0.67	-0.08	-0.79	0.73	1.15	1.21	1.17	1.23	0.38	-1.31	-0.10	-1.48	-0.76	1.29	-0.52	1.07	-0.97	1.62
Srgap1	0.62	-0.60	-0.13	0.44	-1.57	0.10	1.20	0.88	0.75	0.88	-2.74	0.38	-0.77	-0.51	-0.57	-0.30	-1.06	1.01	-0.48	-1.50
Ptrf	0.22	0.27	-0.14	0.73	0.07	0.96	1.30	1.17	1.47	1.21	0.28	1.49	0.03	-0.81	-1.66	-0.70	-1.03	-0.30	0.51	0.02
Plvap	0.06	-0.08	0.31	-0.21	0.78	1.03	0.06	1.85	1.12	1.73	0.39	0.02	0.10	-2.08	-0.13	0.13	-1.62	-0.82	0.17	-1.41
Tm2d1	0.08	0.11	-1.29	-0.93	0.46	-0.11	1.16	0.83	-0.15	1.58	0.21	-1.06	-0.57	0.36	-0.47	-0.52	-0.94	-2.05	0.09	-1.83
Cep164	0.69	-0.25	0.44	1.06	0.39	1.47	1.19	0.94	2.02	1.58	-1.26	-0.05	0.93	-0.58	-0.61	-1.16	-0.07	-0.06	0.41	0.42
Foxk2	0.46	-0.31	-0.53	0.33	0.70	1.93	0.90	0.86	0.52	1.32	0.33	-2.54	-0.78	0.37	0.47	-1.60	-0.21	0.40	-1.20	-0.01
D14Ertd725	-1.24	-0.37	-0.86	-0.59	0.26	-0.66	0.11	0.72	0.36	1.54	0.11	-2.82	-0.65	-1.48	0.03	0.17	0.33	0.61	-0.68	-1.93
Cbln4	0.18	-0.27	-0.28	0.40	0.44	0.76	2.56	0.41	0.83	0.75	0.10	-0.81	0.21	-1.14	-0.65	-0.44	-0.63	-1.56	-0.21	-0.30
Ssh1	-0.46	0.10	-0.82	0.50	-0.15	0.39	-0.38	0.47	1.76	1.77	0.11	-0.31	-1.17	-1.85	0.20	0.19	-1.56	0.08	1.01	0.21
Shank3	1.05	-1.15	-0.19	-0.62	0.26	0.08	0.72	0.91	1.42	0.98	0.62	-0.64	-0.55	-1.15	-0.83	-2.27	1.01	-0.27	-0.88	-0.26
Akr1e1	-0.43	-0.23	-0.38	0.11	-0.51	0.38	1.25	0.61	0.60	0.45	-0.36	0.30	0.24	-0.34	-1.33	-1.77	0.29	0.83	0.65	1.13
Raph1	0.68	-0.24	-0.86	-0.55	-1.03	0.91	0.21	0.17	1.24	0.17	-1.16	-2.04	0.30	-0.50	0.14	0.93	-1.18	0.06	0.23	-1.14
Pfas	0.65	1.02	0.82	1.07	0.57	2.04	1.47	2.22	1.68	1.39	-1.01	-1.40	0.30	-1.00	-0.53	-0.55	-0.05	0.13	-0.76	-1.09
Fam109a	-0.41	-1.02	0.43	-0.74	0.49	0.88	0.38	0.38	1.75	-0.01	1.18	0.70	1.52	-0.85	0.04	0.77	-2.81	-0.38	-1.18	-0.54
Ap5m1	0.21	0.51	0.23	0.26	0.79	1.76	1.23	0.61	1.29	1.71	-0.85	0.50	-0.60	-0.55	0.88	0.26	-0.99	-0.73	-0.44	0.24
Pmpca	0.58	-0.02	0.34	-0.23	-0.72	0.49	1.16	0.04	1.92	0.95	-1.05	0.50	0.39	-0.90	1.43	0.45	-1.25	-0.39	-0.09	-0.93
Meig1	-0.81	0.58	0.36	0.19	-0.27	0.44	1.14	0.46	1.08	1.49	0.14	-0.82	0.02	1.17	-1.01	0.46	0.27	0.46	0.18	0.75
Dffa	-1.23	-0.42	0.15	0.14	0.29	1.28	-0.12	0.55	0.87	0.87	0.74	0.28	0.98	-1.40	-0.64	-2.28	-0.03	0.05	0.58	-1.74
Kndc1	0.59	0.87	1.01	0.73	0.78	2.23	0.89	1.74	1.51	2.04	-0.23	-1.04	-0.61	-1.43	-1.42	-0.34	-0.79	-0.26	-0.89	0.23
Plpp1	0.09	-0.76	0.17	-0.17	-0.91	0.69	1.10	0.59	0.85	-0.39	-3.93	-1.10	0.37	-0.16	-0.43	-0.46	0.89	-1.91	-1.78	0.11
Il17rb	-0.34	0.18	0.03	0.46	-0.56	1.23	0.59	0.15	1.07	1.11	-0.64	-1.41	-0.05	-0.54	0.81	-2.81	-0.68	1.25	0.52	0.31
Mid1	-0.89	-1.09	0.49	0.22	0.16	1.07	0.47	0.86	0.43	0.40	-1.54	0.03	-1.27	-2.13	0.56	-1.31	-0.87	0.47	0.83	-0.16
Slc52a2	0.80	0.94	-0.17	0.72	-0.57	0.66	1.69	0.99	1.36	1.38	-0.74	-1.55	-0.98	-1.80	0.44	-1.70	0.33	1.23	-1.88	0.54
Fam204a	1.42	0.71	0.46	0.44	-0.82	1.56	1.28	1.33	1.41	0.96	0.04	-1.97	0.10	0.37	0.76	-1.03	-0.30	0.24	-0.59	0.84
Dact3	1.82	0.49	1.11	0.81	0.40	1.67	1.69	1.29	1.87	2.42	-0.48	-0.50	-1.14	-1.19	-0.51	-0.86	-0.38	0.02	-1.65	-0.55
Zfp956	0.34	0.98	1.24	-0.10	0.17	1.85	1.37	1.23	0.90	1.54	-1.13	0.38	-0.09	-1.33	-0.32	1.11	0.43	0.01	-0.01	-0.18
Ly6g6e	-0.49	-0.08	-0.28	0.78	0.10	0.55	1.41	0.64	0.32	1.36	-0.70	0.76	-3.41	-1.41	0.50	1.21	-0.70	-0.84	0.94	-1.80
Fam229b	-0.37	-1.02	-0.40	-0.51	-0.83	-0.55	0.22	0.59	-0.07	0.92	-1.07	-1.07	1.07	0.84	-1.31	-0.60	0.34	-0.28	0.17	-1.07
Atp13a3	-0.28	0.43	0.63	1.31	-0.19	0.79	0.92	0.72	1.86	1.83	0.64	-0.19	0.88	-1.48	0.07	0.01	-1.18	0.63	0.19	-0.33
Fa2h	-0.67	0.49	-0.13	0.46	-0.31	1.40	0.59	0.32	0.65	1.04	0.36	-2.19	-0.68	0.76	-1.91	-0.56	0.34	0.72	-0.12	0.38
Phxr1	-0.05	-0.65	0.04	-1.45	-0.27	-0.10	0.42	0.12	0.68	0.64	-0.45	-0.10	-0.84	-1.71	0.57	-1.24	-0.75	0.73	0.01	0.86
AA645497	-0.77	-1.19	0.22	-0.48	-0.10	0.09	0.10	0.67	0.26	0.66	0.49	0.28	0.19	-1.70	-0.32	-1.28	-4.40	-0.26	0.62	-0.16
Plekhg4	0.26	-0.59	-0.81	-0.20	-0.55	-0.07	0.41	0.67	0.54	0.64	-5.02	0.17	0.55	0.12	-0.57	0.00	0.36	0.69	-0.26	0.25
Farp2	0.34	0.13	0.68	-0.03	0.70	0.53	1.08	1.92	1.42	0.97	-0.21	-0.73	0.72	-0.02	-1.06	0.43	-1.41	0.58	-0.43	-0.72
Pgap2	0.75	1.36	0.13	0.78	0.38	0.79	1.53	1.92	1.74	1.48	-0.52	0.33	0.06	-1.85	-0.45	-0.47	-1.09	0.44	0.50	0.41
Rps6ka4	0.69	-0.19	0.77	1.16	0.23	0.83	1.59	1.58	1.03	1.66	-0.44	-0.19	-1.61	-1.83	-1.59	-0.39	-1.13	-0.07	-0.75	-0.37
Hdac3	1.03	1.03	0.65	0.42	0.63	1.31	1.03	2.21	2.15	1.09	0.78	-0.88	-0.56	-0.07	0.06	-1.59	-0.92	-0.08	-1.01	-0.86
Rab24	1.17	0.66	0.07	1.10	0.41	1.55	1.66	1.80	0.87	1.50	0.50	0.47	0.56	-1.14	-0.92	1.65	-0.93	-0.22	-0.42	-1.53
Aldh9a1	1.00	0.15	0.63	0.24	0.43	0.99	0.69	1.16	2.47	1.11	-0.33	-0.88	-0.04	-1.03	-0.35	-0.03	-1.34	-0.55	0.42	-1.30
Rsph10b	0.89	0.26	0.82	0.02	-0.29	1.45	0.74	1.06	1.05	1.27	0.56	-2.71	-0.66	-0.38	-0.30	-0.91	-1.22	-0.20	0.24	-0.68

Rab34	-0.11	0.84	0.77	0.45	0.23	0.85	1.31	1.11	1.74	1.01	-0.81	0.40	-0.10	-1.25	-0.62	-0.50	-0.46	-0.40	0.07	0.72
Gpr146	0.82	-0.07	0.61	0.05	0.52	0.92	1.85	0.64	1.34	0.85	-1.50	0.34	-0.51	-0.28	-0.22	0.60	-2.11	-0.64	-0.06	-0.02
Slc7a6	0.67	0.24	0.36	0.85	0.49	1.38	1.38	1.90	0.65	0.84	-0.95	-0.44	0.20	-0.66	1.54	-0.09	0.43	0.30	0.45	-0.09
Gins1	0.11	-0.37	-0.50	0.04	0.65	0.40	0.69	0.47	0.87	0.79	-0.14	0.10	-0.81	-0.79	1.07	-1.28	-4.02	0.23	0.62	-1.54
Alg2	1.10	1.18	1.33	0.95	1.33	1.55	1.47	1.74	2.31	2.01	-0.36	-0.74	-0.34	-0.53	-0.49	-0.68	-0.68	-0.06	-0.89	-1.17
Tle2	0.72	1.04	1.25	0.72	1.05	1.77	1.47	1.56	1.78	1.35	-0.46	-0.65	0.06	-0.57	-0.80	-1.47	-0.26	-0.16	-0.11	-0.33
Grwd1	0.88	0.74	0.58	0.62	1.18	1.21	1.29	1.55	1.56	1.47	0.38	-0.99	-1.08	-1.68	-0.55	0.52	0.95	-0.34	-1.03	-1.62
Rab5a	0.56	-0.01	0.74	0.42	0.05	1.11	0.71	1.05	0.90	1.04	0.54	-1.18	1.27	-0.12	-1.01	-0.45	1.02	1.38	0.60	0.49
Tubb5	-0.86	-0.18	0.23	-1.99	-0.51	0.98	0.32	0.33	1.99	2.95	0.28	0.62	-0.14	-1.54	-0.13	0.56	-0.31	0.80	-0.71	0.16
Spire1	-0.41	-1.63	-2.01	-1.88	-1.51	0.35	0.35	0.69	-0.04	0.48	0.88	0.54	-0.22	-0.32	-1.41	0.55	0.01	0.40	0.66	0.16
Fmc1	-1.38	-0.52	-3.17	-0.59	-0.97	0.99	0.71	-1.07	0.99	0.97	0.99	-0.33	0.41	0.13	-0.44	-0.17	-1.15	-0.54	-0.81	0.71
Hnmt	-0.34	-1.09	-1.60	-0.76	-1.57	1.43	1.39	0.76	-0.85	0.82	1.68	0.12	0.73	0.88	-0.85	-1.34	0.80	-0.26	1.10	-0.68
Shtn1	-1.91	0.27	-0.26	-0.27	-1.56	0.91	0.10	2.02	0.72	1.21	-0.37	-0.34	-0.58	-0.51	0.90	0.48	1.52	0.09	-0.47	-0.56
Iars2	-0.81	0.52	0.25	-1.44	-4.35	0.70	0.61	0.26	0.27	0.54	0.32	0.67	0.73	0.41	-0.14	0.52	0.12	-0.59	0.27	0.44
Huwe1	0.76	0.08	-1.38	-2.98	-2.57	0.36	0.85	0.34	0.65	-0.16	0.81	0.61	0.36	1.09	0.85	0.86	0.39	-0.29	0.16	0.78
Wnt8b	-4.20	0.18	-0.07	-0.71	0.34	0.75	0.32	0.84	0.86	0.87	1.63	-1.31	-0.55	0.26	-0.82	-0.43	-0.41	-1.07	0.01	0.53
Gm19313	-0.71	-2.01	-0.99	-0.80	-0.87	0.32	0.27	0.52	1.48	0.05	-0.64	-0.92	-1.96	0.10	-1.38	-0.63	-2.15	-0.72	0.86	0.37
Scgn	-0.24	0.69	-2.49	-3.09	0.41	0.76	1.16	0.85	0.87	-0.45	-0.56	1.28	0.02	-0.10	0.87	-0.89	0.31	-0.91	-0.77	-0.85
Tmtc3	-0.82	-0.66	-1.76	-1.49	-0.84	0.92	0.68	0.38	0.02	0.30	-0.39	0.75	-1.05	0.15	0.55	-0.21	-0.41	0.47	-0.20	0.38
Tshz3	0.39	-0.32	-1.48	-0.85	-0.89	0.21	1.51	1.57	-0.30	1.67	0.35	0.46	-0.66	-1.83	1.68	0.36	-1.07	-1.15	-0.15	-0.08
Emsy	0.94	-0.60	-3.04	-0.26	-1.25	0.19	1.17	0.75	0.62	0.89	-0.05	0.83	0.21	0.40	-0.42	-1.43	-1.04	0.80	0.36	0.59
Nob1	0.35	-1.36	-0.96	-1.03	-0.39	1.43	-0.52	0.61	1.14	1.53	-0.03	-0.81	-1.31	-1.34	0.52	1.96	-0.72	1.11	-2.07	0.30
Xlr	-2.30	0.74	-1.41	-0.05	-2.09	0.28	0.95	0.63	1.15	-0.71	-0.40	-0.46	-0.48	-0.58	-1.94	1.08	0.00	0.97	1.69	1.76
Naf1	-0.95	-1.24	-0.04	-0.17	-0.79	0.80	0.73	0.34	0.36	1.78	-0.62	-0.07	-0.51	1.11	-0.35	1.68	-0.88	0.37	0.28	2.11
C76872	-0.43	-1.28	-0.29	-2.39	-1.21	0.85	-0.26	-0.11	0.66	0.35	-0.83	0.14	-1.88	1.29	0.96	-0.71	0.80	-0.45	0.59	-0.12
Blvra	-1.33	-0.10	-1.11	-0.88	-1.97	-0.20	1.94	-0.07	0.61	-0.60	1.52	-0.34	0.37	0.11	-0.77	0.03	-0.86	-0.96	0.06	0.11
Kcnip1	-0.18	0.66	-0.35	0.94	-2.86	1.04	1.41	0.80	1.01	0.89	0.30	0.96	-0.45	-0.17	0.96	0.26	0.61	0.09	0.17	0.78
Rps15	0.32	0.61	0.46	-2.43	-0.26	0.49	1.10	0.96	1.89	1.08	-0.52	0.46	-1.84	-0.12	-0.06	0.22	-1.64	0.34	-0.20	1.08
Ovgp1	-1.54	-0.53	-0.44	-0.65	-0.28	1.71	-0.36	0.91	0.49	0.59	-0.13	-0.35	-2.23	-0.28	1.07	0.73	0.96	1.83	-0.52	-0.45
Tbck	0.05	-2.81	-0.32	-2.42	-0.51	0.42	0.31	0.50	-0.55	-0.15	0.82	0.66	0.70	-0.02	0.68	0.71	0.54	-3.51	0.32	0.47
Tmem217	-0.08	-0.01	-2.91	-0.34	0.10	-0.40	0.95	0.64	0.79	1.23	0.23	1.03	0.82	-1.02	-0.96	-0.45	0.64	1.28	0.28	0.86
Per1	0.40	-1.14	-0.02	0.04	0.32	0.55	1.37	1.24	1.02	1.84	0.25	0.26	-1.54	0.20	1.38	0.29	-0.35	-0.50	0.96	1.02
Fam45a	-0.04	0.92	0.21	-0.93	-1.28	1.25	0.15	0.94	1.62	1.31	-0.17	1.21	-0.45	-0.41	0.14	0.83	-1.37	-0.11	1.45	0.70
Oxr1	-0.01	-0.50	-1.27	-2.18	-0.87	0.15	-0.38	-0.20	0.27	1.74	1.79	1.56	1.47	0.18	0.55	1.00	0.58	0.18	-1.88	-1.63
Zfp566	-0.09	-1.25	-0.12	-0.77	-2.35	0.43	1.18	-0.96	0.52	0.63	0.65	-1.46	-1.67	1.08	0.40	0.70	0.47	-0.65	0.07	-0.35
Gm38604	-1.62	0.71	-0.96	-0.68	-0.55	0.78	1.13	1.28	0.09	-0.04	0.46	-0.89	0.44	-0.28	-2.23	1.21	0.26	0.69	-0.53	0.25
Cradd	1.67	-0.35	-0.77	-1.38	-1.00	1.62	0.73	0.76	0.71	0.60	0.61	-0.01	-1.25	-0.46	0.69	0.71	-2.37	-1.34	-0.04	-0.16
Sowahb	0.14	-0.62	-1.34	-0.81	-1.60	1.26	-0.56	1.34	-0.82	0.79	0.26	-0.71	-1.95	2.33	0.84	-0.82	-0.65	0.14	-1.17	-0.13
Gxylt1	0.28	-1.07	-1.14	-0.50	0.74	1.35	1.10	1.09	1.05	-0.06	-0.34	-0.78	-0.29	1.16	0.47	0.37	0.76	-0.19	-0.98	1.70
Zfp78	0.09	-2.31	-0.87	0.49	0.05	0.78	0.36	1.83	-0.34	1.01	0.83	1.11	-0.85	-0.20	1.29	0.29	0.53	-0.48	-0.06	-0.26
Wdr6	-0.19	0.78	-0.50	-0.51	-0.11	2.45	1.95	0.70	0.46	0.07	0.93	-0.42	0.71	-0.43	0.16	0.15	1.85	0.79	0.65	-0.83
Rangap1	0.03	-0.23	-1.92	-0.07	-0.63	0.62	0.78	0.52	0.54	0.88	0.26	1.52	0.28	0.41	-0.44	-0.19	-0.33	-2.29	0.16	-0.11
Krt42	-0.28	-0.25	-1.06	-0.11	-2.28	0.30	0.04	1.61	-0.41	0.63	-1.28	0.78	-1.99	0.49	0.51	-1.02	-0.19	-1.07	0.47	-0.13
Patl2	-0.71	-2.61	-1.46	-1.06	0.35	0.65	0.36	1.04	-1.21	-0.32	-0.02	1.64	-0.45	0.43	-2.36	-0.58	1.08	0.05	0.19	-0.65
Map3k10	-0.21	-0.14	-2.88	-1.23	-0.34	-0.33	0.61	1.20	-0.61	0.33	0.45	-0.37	-0.13	1.59	0.75	-0.42	-0.44	1.11	-0.84	-1.28
Nnt	-0.13	0.41	-1.23	-0.15	-2.71	0.23	0.65	0.73	0.31	0.19	0.76	0.71	-0.52	0.10	0.43	-0.44	0.13	-0.11	0.30	0.22
Zfr	-0.77	0.39	0.38	-1.65	-1.78	0.44	0.50	0.31	0.42	0.81	0.25	-0.32	0.60	0.26	0.62	0.20	0.87	-4.91	0.09	0.15
Kcnq5	0.16	-0.29	-1.68	-1.81	-1.29	0.47	0.67	0.20	0.12	-0.47	1.70	-0.39	0.37	1.10	-2.05	-0.60	0.27	0.35	-0.34	-1.85
Ehmt1	-0.45	-2.13	0.07	-1.23	-0.80	0.85	-0.87	-0.87	0.58	1.62	0.67	0.41	-2.61	-0.93	0.97	-0.67	-0.16	0.19	-0.59	0.24
Tmem189	-1.05	-0.44	0.21	-1.14	0.71	0.24	0.78	0.90	1.14	1.09	1.29	0.44	0.55	-0.65	0.82	0.64	0.06	-0.23	-0.71	0.41
Nek9	0.32	0.32	-0.85	-0.46	-1.79	-0.07	0.29	0.61	1.01	1.49	-0.96	0.07	0.45	-0.05	-0.25	1.55	-0.15	-0.30	1.94	1.23
Fam49b	-0.78	-1.00	-1.82	0.19	-1.58	0.60	0.48	-1.15	-0.62	1.47	0.34	-0.63	-0.82	0.84	-1.17	0.94	-0.69	0.70	-0.67	0.37
Defb10	-0.73	0.09	-0.72	-2.13	-0.49	0.30	-0.28	0.22	0.59	0.91	-0.48	0.50	0.29	-1.86	1.02	-0.16	-0.67	0.04	-2.42	0.89
Sae1	-0.17	-0.47	-1.57	-0.89	0.17	0.00	0.73	0.27	1.12	0.59	-0.61	0.56	0.20	0.60	-0.68	1.34	0.86	-0.78	0.95	-1.09
Rab11fip1	-0.55	0.06	-0.33	-2.10	-1.41	0.97	0.30	-0.06	0.16	-0.12	0.54	0.68	-0.50	0.74	0.55	-0.12	-0.01	0.82	1.12	-1.66
Kdm6b	0.63	-1.62	-0.38	0.00	-1.92	0.74	0.74	0.44	-0.49	0.86	-1.48	0.24	-1.95	0.70	-0.49	-0.47	0.96	-0.29	0.33	0.91
Fbxl14	0.67	-0.16	-0.07	0.16	-1.85	0.87	1.32	0.98	0.91	0.20	0.02	-0.17	0.65	0.17	-0.49	0.76	0.51	-0.48	1.13	1.14
Ccdc88a	-1.23	-0.74	-1.05	0.61	-1.22	0.78	-1.05	1.20	0.80	0.13	0.39	-0.22	-0.44	0.04	0.04	-0.84	-0.47	-0.13	0.66	0.31
Cxcl16	-0.23	-0.20	-0.83	-0.29	-1.65	0.96	-0.49	1.27	-0.71	1.26	0.77	0.34	-0.62	-0.73	-1.52	1.20	-0.11	0.51	0.61	0.67
Ccdc122	0.64	0.77	-1.39	-0.75	-1.25	1.59	0.40	0.00	0.90	0.56	-1.37	0.15	-1.46	-0.23	0.16	0.75	-0.10	-0.49	1.78	0.39
Ccdc138	-1.06	-1.68	-0.30	0.24	-0.53	-0.06	0.89	1.39	-0.56	0.39	0.26	0.69	0.62	-0.10	-1.22	1.88	-0.50	-0.92	-0.88	-0.19
Emp2	0.28	-0.84	-1.28	-2.30	-1.42	0.73	-0.76	-0.86	0.05	0.44	0.78	0.50	-2.62	0.31	-0.33	0.35	0.18	0.70	-1.63	0.96
D10Jhu81e	-1.93	-0.28	0.43	-0.53	-0.03	0.72	1.21	0.41	-0.20	0.65	0.36	-1.57	0.13	0.01	-0.65	-1.10	-0.67	-1.44	1.62	1.63
Cr2	0.28	-1.55	0.31	-0.14	-1.74	0.85	0.21	0.23	0.24	0.71	0.45	1.20	-0.73	-0.19	0.20	0.26	-1.13	0.35	1.01	0.47
C1ra	-0.98	0.23	-0.39	-0.93	-1.26	-0.18	0.80	0.94	-0.54	0.67	0.37	0.65	-0.33	0.44	-0.15	1.26	0.07	0.98	0.56	-0.40
Chd9	0.20	0.17	-1.10	0.17	0.12	1.46	1.05	0.62	-0.24	1.68	1.28	-0.60	0.20	-0.85	0.66	-0.16	1.12	-0.32	0.10	-0.22
Hmgb3	-0.43	0.86	-0.28	-0.48	-0.35	1.40	0.69	0.35	1.33	0.38	1.06	-0.13	1.09	-1.27	0.60	1.74	-0.85	-0.20	0.44	1.32
Scyl2	0.35	-0.34	0.40	-1.17	0.64	1.90	0.49	0.41	0.51	1.35	0.49	-0.60	-0.67	0.52	0.60	0.81	-0.14	-0.16	0.80	-0.09
Ubn2	0.41	-1.40	-1.47	-1.16	-1.67	0.18	-0.04	0.36	-0.01	-1.00	-0.49	-0.42	-1.89	0.17	0.55	-1.14	-0.07	0.67	-0.27	1.61
Efcab6	-0.13	0.49	0.58	-1.22	-0.38	0.22	1.11	0.91	0.36	1.47	1.33	0.61	0.51	-0.29	-1.48	1.26	0.30	0.42	-1.58	0.24
Dxo	-0.28	0.52	-0.08	-0.85	0.40	1.43	0.11	1.50	0.90	0.48	0.19	1.09	0.06	1.14	-0.55	-0.28	-0.44	-0.09	0.20	-0.06
Mvb12a	0.36	-0.40	-0.26	-1.69	-0.67	0.99	0.19	-0.48	1.05	0.26	-1.42	1.76	0.45	0.68	-0.11	-1.33	0.29	0.23	0.33	-0.65
Fam173a	-1.13	0.55	-1.74	-0.30	-0.43	-0.19	0.25	0.88	0.28	0.40	-0.18	0.28	-1.74	-0.17	0.78	0.70	0.56	0.09	-0.32	1.60
Muc13	-0.42	-0.57	-1.51	-0.11	0.49	0.07	0.96	0.81	-0.23	0.80	0.64	-0.31	0.87	0.44	-2.06	0.63	0.62	-2.34	0.65	0.96
Akr1b7	-0.63	-0.55	-1.37	-0.14	-0.14	0.29	1.38	0.23	-0.46	0.25	0.47	-1.37	-3.12	1.52	0.46	-1.98	0.12	-0.97	0.85	-1.33
Trnau1ap	-0.21	0.42	-0.45	-1.16	-0.64	0.24	0.82	-0.46	1.20	0.63	-0.30	-0.23	0.57	0.67	-0.22	1.46	-0.50	-0.20	0.81	0.19
Glmn	-0.45	-0.55	-0.08	-1.19	0.27	0.81	-0.42	0.12	0.67	1.04	0.32	0.77	-1.40	1.12	-0.94	0.81	1.62	0.17	1.36	-0.87
Dach1	-0.57	0.68	0.40	-0.13	-0.74	0.83	0.85	0.06	1.37	0.63	0.68	0.70	0.95	0.64	-1.35	0.99	-0.41	0.26	-0.13	0.22
Zfp111	-0.14	0.45	-0.71	0.42	-0.27	0.62	0.59	1.10	0.85	0.65	1.22	-0.26	0.48	0.16	1.33	-0.01	0.64	-1.41	-0.10	-0.37
AI956758	-0.99	-0.58	-1.26	-1.40	-1.02	0.77	-0.58	-0.68	-0.27	-0.42	-0.23	0.31	-1.10	-0.18	-0.51	0.20	-0.79	-0.46	1.99	1.42
Bbs7	-3.23	-1.26	-1.17	-2.76	-2.71	0.40	-0.44	0.15	0.13	0.49	0.50	0.60	0.44	0.91	0.01	0.81	-0.43	0.07	0.75	0.40

Mastl	-4.49	-0.07	0.24	-2.20	-1.28	0.38	0.68	0.69	0.79	-0.43	0.21	0.22	0.19	0.22	-0.26	0.27	-0.03	-1.00	0.78	-1.57
C78760	-1.13	-2.25	-1.45	-1.72	-0.71	0.53	-0.03	0.98	0.25	0.20	0.29	-0.01	-1.26	1.41	0.76	0.17	1.78	-0.66	0.64	1.10
Etl4	-0.60	-1.74	-1.05	-0.86	-2.34	0.13	0.27	0.54	0.25	1.11	0.48	0.31	0.05	0.52	0.34	1.00	-0.06	-0.09	0.34	1.24
Ppp2r2b	0.33	-3.17	-1.33	-2.11	-1.09	0.51	0.36	-0.01	-0.51	1.09	-0.01	0.95	0.65	-0.21	0.26	0.69	0.83	0.86	0.69	0.26
Heatr1	-1.58	-4.25	-0.52	-1.65	-0.32	0.31	0.28	0.51	-0.89	-0.30	0.87	0.84	-1.33	0.58	0.74	0.16	1.18	-0.18	0.23	0.81
AA415038	-3.21	-1.30	-1.76	-1.01	-0.66	-0.15	0.31	0.09	-0.10	0.06	0.29	0.31	-0.20	0.35	0.69	0.06	-0.21	-0.61	0.08	0.62
Vax1	-1.68	-1.78	1.29	-1.98	-0.83	1.40	1.19	0.63	-0.79	0.34	-0.06	-1.75	0.80	-0.30	0.73	0.47	-0.17	-0.27	-0.33	-0.04
Foxo3	-2.29	-1.80	-0.57	-1.23	-1.01	-1.36	0.29	0.17	0.09	1.60	0.25	1.72	0.08	0.31	0.63	-0.34	1.19	0.01	-0.11	0.80
Ass1	0.91	-2.96	-0.25	-1.71	-2.06	0.70	-0.38	1.24	0.52	-0.50	0.43	-0.02	-0.16	-1.40	1.07	0.15	1.22	-0.25	-0.60	0.73
Piga	-1.53	-0.04	-0.90	-1.46	-1.89	-0.32	0.10	-1.25	1.32	1.91	2.46	-0.08	-0.21	-0.33	1.42	0.60	1.28	-0.46	1.07	0.56
C80998	-1.31	-1.43	-2.20	-1.56	-0.54	0.19	0.57	0.04	0.06	-0.36	-0.06	-0.63	-1.41	0.83	1.92	1.22	0.15	-0.10	-0.01	1.16
Syp	-2.93	-3.11	0.79	-0.76	-0.04	0.62	-0.01	0.13	-0.18	0.88	-1.20	-1.57	0.61	1.11	0.37	0.05	-0.44	0.34	-2.10	0.14
Arhgef18	-0.86	-1.25	-2.52	-3.12	-0.37	-0.31	-0.18	-0.05	0.74	-0.87	0.71	1.31	0.81	0.52	0.80	-1.30	0.84	0.08	-1.15	0.19
Cwc27	-0.89	-0.44	0.26	-2.47	-1.01	0.92	0.08	0.01	1.61	0.18	0.02	-0.70	-0.32	0.66	0.92	0.40	0.06	1.48	1.10	1.12
St8sia4	-1.89	-0.67	-1.77	-0.50	-0.94	-0.10	0.61	1.56	-0.96	0.42	-0.33	0.17	0.00	2.03	1.21	-1.35	0.42	0.25	-0.20	-1.46
Plcl1	-0.58	-4.58	-1.72	-0.80	-1.59	-0.57	-0.17	-0.44	-0.28	-0.52	0.77	0.53	0.56	0.81	0.48	0.52	0.58	0.51	0.65	0.07
Lrch3	-1.04	-2.22	-1.60	-1.86	-2.21	-0.40	-0.86	-0.55	0.88	-0.75	0.71	-1.42	0.42	0.40	0.14	1.36	0.63	-0.43	1.04	1.19
Vill	-0.61	-2.46	0.01	0.10	-3.59	0.58	-0.48	0.33	-0.19	0.45	0.19	0.34	0.71	-2.77	0.01	0.72	0.20	1.02	0.30	-0.40
Ice2	-1.99	-0.68	0.58	-1.01	-0.96	1.14	0.56	0.20	0.63	0.59	1.38	1.00	0.51	-0.25	0.03	-0.24	0.23	0.38	-0.35	0.50
Gm14964	-0.58	-2.54	-0.92	-1.48	-1.49	0.01	1.29	0.07	-0.51	-0.72	-0.68	-0.23	0.49	1.90	-1.12	0.28	-0.40	-0.26	-0.65	0.13
Erlec1	-2.17	-1.19	-1.39	-1.74	-2.05	0.35	-0.84	-0.42	0.48	-1.00	0.84	0.54	-0.31	0.01	1.50	0.08	0.30	0.37	-0.01	0.49
Strbp	-0.11	-2.49	-1.86	-2.11	-1.33	0.29	-1.69	-0.34	0.13	0.75	-0.09	-0.26	-1.43	0.18	0.61	-0.51	0.54	0.10	-0.53	-0.96
Tango2	-1.91	-1.81	-1.24	0.92	-1.82	1.16	-0.65	-0.02	0.55	0.07	-0.48	0.38	1.14	0.82	-1.00	-0.49	0.60	0.23	0.50	1.89
Zdhhc21	-2.69	-0.76	-0.95	-1.05	-0.25	0.78	-0.12	0.04	2.07	-1.53	-0.29	-0.35	0.41	-0.21	-0.20	-0.11	0.48	0.68	0.87	0.61
C80425	-3.76	-2.10	-0.15	-1.64	-0.69	-0.60	-0.94	-0.27	0.40	-0.05	0.46	0.64	0.58	0.81	0.57	1.27	-0.03	0.65	-0.40	0.64
Prrg1	-0.81	-1.79	-1.48	-0.47	-1.13	-0.33	1.09	-0.01	1.17	-0.72	0.09	0.11	0.53	0.46	0.42	0.04	0.62	-0.37	-1.25	-0.73
Arx	-1.43	-3.34	-2.51	-1.12	-0.97	-0.44	-1.12	-0.85	-0.30	-0.09	0.10	0.51	0.01	-0.44	0.22	0.96	0.32	-0.21	1.54	1.80
Sp110	-3.00	-1.59	-0.16	-0.10	0.32	-0.30	-0.18	0.97	0.43	1.11	0.50	-2.72	-0.49	-0.29	0.55	-0.86	-0.29	0.48	0.91	1.45
Fzd4	-0.01	-1.19	0.41	0.48	-2.29	0.79	1.09	1.40	1.50	-0.88	0.72	-3.05	1.24	0.55	1.20	0.89	-0.23	0.73	-0.10	0.29
Pias1	-1.16	-1.73	-1.24	-1.79	0.58	-1.25	0.23	-0.25	1.05	1.34	-0.43	1.23	-0.59	1.10	0.71	1.42	-0.44	0.41	0.85	-1.66
Atat1	-0.39	-1.06	-2.46	-0.14	-1.25	-0.40	-0.60	1.24	0.50	0.40	-1.13	-0.35	0.93	0.32	0.31	0.86	1.62	0.21	0.38	0.12
Elmsan1	-1.64	-1.54	-0.92	-0.54	-0.74	0.99	0.42	-0.45	-0.07	0.05	-1.62	-0.97	-0.59	0.58	1.92	0.52	0.99	1.11	-0.16	-0.04
Wdr76	-0.46	-2.43	0.58	0.08	-1.80	0.54	0.04	0.61	0.60	0.49	-0.06	1.03	0.42	0.56	0.64	-2.71	-0.20	0.29	0.42	0.49
D15Wsu126	-0.93	-1.61	-0.35	-1.78	-1.65	0.62	-1.03	0.75	-0.36	-0.01	0.15	-1.07	-0.96	-0.01	-0.19	-0.73	-0.96	1.13	0.75	1.01
Rnf217	-1.74	-0.38	-0.56	-0.77	-1.72	0.97	0.19	0.74	-1.86	1.02	0.61	-0.65	0.11	0.65	0.10	1.11	0.37	0.54	-0.12	-0.10
AW555355	-2.39	-2.63	-1.18	-1.50	-0.60	-0.52	-1.13	-0.10	0.43	-0.77	-0.11	-0.39	-0.59	0.23	0.29	0.26	-0.69	0.19	0.55	0.23
Cd200r4	-2.11	-1.49	-1.39	0.21	-1.30	-1.16	-0.50	0.70	0.03	0.98	-0.09	0.75	0.25	0.48	0.70	0.86	0.75	0.03	1.33	1.23
Gpatch8	-0.67	-2.38	-0.43	-1.01	-0.75	0.58	0.25	-0.97	0.34	0.55	0.51	-1.14	-1.57	-0.06	-0.17	-1.66	0.75	0.68	-0.21	-0.20
LOC553089	-2.58	-2.00	-2.80	-0.49	0.07	-0.27	-0.58	0.03	0.21	-1.22	-0.17	0.44	-0.31	0.57	1.03	-0.40	0.75	0.97	-0.03	0.90
Amy1	-1.47	-1.09	-1.18	-1.28	-1.37	0.48	-0.57	-0.21	0.15	-0.32	-0.07	0.34	0.60	0.38	2.44	0.82	0.58	1.14	1.05	0.39
Trim44	-1.15	-0.73	-1.12	-0.83	-2.01	-0.30	0.08	-0.66	0.80	0.14	0.07	0.42	0.46	2.84	0.48	-1.77	0.46	0.56	-0.70	-0.15
Nampt	-2.07	0.61	-1.88	-2.45	-1.58	-0.20	-0.62	-0.64	-0.65	0.46	0.64	1.05	0.19	-0.20	0.20	1.34	1.09	1.50	0.28	1.16
Thbs2	-2.10	-1.15	-0.51	-0.49	-1.39	0.44	-0.41	-0.46	0.95	-0.46	-0.07	1.13	0.08	-1.52	0.84	0.27	0.85	0.26	1.68	1.19
Slc40a1	0.17	-1.03	-0.63	-0.39	-0.59	0.02	1.24	-0.10	1.05	1.01	-0.36	-0.57	0.86	0.79	0.43	0.32	-0.98	1.39	0.39	0.61
D930001B0	-0.67	-0.49	-2.32	-2.30	-1.74	-0.59	-1.43	-1.09	0.35	0.89	1.03	0.28	-0.21	-0.37	1.61	-1.01	-0.77	0.62	-0.43	-0.67
Ankrd33b	-1.65	-1.64	-1.58	-2.32	-1.65	-0.69	-1.84	-0.26	-0.85	0.40	0.37	0.59	-0.04	0.13	0.15	0.15	0.36	0.41	0.18	-1.34
Nr1d2	-1.26	-1.15	-1.90	-1.05	-0.35	-0.43	0.14	-0.35	0.23	0.30	1.07	0.70	0.81	0.77	1.42	-0.02	-0.26	1.49	1.18	2.65
Tnfaip6	-1.79	-0.88	-0.82	-0.93	-1.65	0.17	0.35	0.27	0.17	-1.50	0.45	0.59	0.69	-0.56	0.58	1.45	0.13	0.71	2.41	1.71
Bvht	-1.14	-0.69	-1.45	-1.39	-2.38	-1.46	0.98	-0.02	0.14	-1.27	0.65	-1.72	-0.94	1.39	1.39	-0.33	0.44	-0.23	0.91	-0.49
BC028789	-1.26	0.51	-1.41	-1.98	-0.50	0.56	0.47	0.18	-0.91	0.49	0.30	-1.47	1.63	-1.47	0.45	1.67	0.37	1.00	-0.88	0.92
Mfsd2b	0.33	-2.16	-0.35	-1.01	0.33	0.87	0.49	0.60	-0.04	0.55	0.50	0.55	0.53	0.02	1.05	0.44	1.03	0.66	0.49	0.39
Plk4	-0.71	-1.24	-1.75	-1.20	-2.37	-1.33	-1.41	0.17	0.67	-0.06	0.76	1.08	0.27	-0.26	0.33	0.67	0.75	0.70	0.57	1.03
Eif1a	-1.26	-0.19	-0.65	-0.31	0.29	0.31	1.01	-0.26	-0.66	-1.20	1.22	-1.28	1.75	-0.58	0.81	1.04	1.25	-0.34	1.18	0.49
Lonp2	-1.27	-1.09	0.33	-1.43	0.56	0.77	-0.03	0.20	0.40	1.03	-1.27	0.46	0.61	-0.85	0.88	0.26	-0.78	0.48	-0.61	0.96
Slc44a1	-1.58	-3.04	-0.34	-0.56	-0.25	0.23	-0.03	-0.22	-0.44	-0.09	-0.45	-0.16	0.64	1.05	1.04	1.23	0.28	1.21	0.49	0.86
Nkain2	-1.91	-0.04	-1.13	-1.99	-0.10	0.71	-1.05	-0.10	0.51	-0.04	0.09	0.69	0.13	0.52	1.49	-0.86	1.69	1.57	-0.93	-1.70
Polr3e	-0.78	-0.77	-1.12	-1.33	-1.98	-0.88	-0.21	1.29	-1.40	0.38	0.16	0.24	0.13	0.65	1.12	1.13	-1.14	1.19	1.10	0.49
Zfp942	-0.62	0.48	-0.68	-0.24	-0.11	0.28	0.51	0.80	2.28	0.08	-0.87	0.26	2.69	0.08	0.93	0.85	0.76	0.17	1.24	-0.20
Bcl2	-2.09	-0.75	0.38	-1.24	-0.54	0.03	-0.08	-0.19	0.61	0.46	-0.77	-0.88	-0.17	0.20	-0.05	0.44	-0.78	-0.49	0.88	2.08
Fign	-2.23	-0.39	-0.53	-0.23	-0.78	0.42	0.27	-0.23	0.33	0.11	0.92	0.03	-0.91	0.25	-0.04	0.42	-0.24	0.55	0.75	0.30
Six4	-0.25	-2.01	0.44	-0.28	-1.47	0.11	0.39	-0.46	1.08	0.36	-0.22	0.88	-0.46	1.13	0.91	0.85	1.13	-0.90	0.35	-1.11
Rhou	-1.11	0.06	-0.34	-1.29	-1.64	0.28	-1.08	0.17	0.70	0.62	0.06	0.85	-0.19	1.04	0.67	1.57	0.87	0.20	1.36	1.77
Zfp189	-1.86	-1.56	-1.57	-0.26	-0.67	0.28	0.10	-0.48	-0.14	-0.68	0.30	-0.53	0.80	0.92	0.30	-0.28	1.21	1.53	0.92	-1.16
Gpr158	-1.12	-1.50	-1.73	-1.31	-2.82	0.01	-0.40	-0.83	-0.78	-1.70	0.15	0.91	0.47	0.83	1.20	0.27	1.53	0.32	0.23	-0.33
AU021128	-0.65	-2.15	-1.66	-1.11	-1.04	0.17	-0.16	-1.04	-0.11	-0.78	0.56	0.03	1.10	-0.59	0.89	0.52	0.77	0.72	0.73	-1.88
Cpsf7	-0.62	-1.17	-1.22	-0.86	-1.02	0.01	-0.67	0.11	0.75	-0.46	-0.61	-2.44	0.27	0.66	0.40	-1.04	-0.44	0.60	0.52	-0.10
Rbm3	-1.40	0.11	-1.35	-0.80	-0.75	0.48	0.09	0.41	-0.30	-0.27	0.39	-0.42	0.04	0.00	1.12	1.18	0.81	1.61	1.38	-0.38
Cntn1	-0.98	-2.06	-1.64	-2.02	-1.68	-0.58	-1.41	-1.32	0.37	-0.86	0.70	0.34	0.24	0.23	0.90	-0.75	0.05	-0.03	-1.03	-1.04
D11Ertd717	-0.41	-2.25	-1.98	-0.62	-1.07	-0.61	-0.89	-0.43	0.21	-0.07	-0.19	-1.84	0.47	-0.63	0.27	-1.34	0.78	-0.99	-0.41	-0.08
Gbf1	-2.07	-2.19	-2.31	-0.92	-1.80	-0.60	-0.64	-1.05	-1.37	-1.10	0.69	0.46	0.41	0.61	0.96	0.58	1.06	0.44	0.40	-0.29
Coa6	-0.98	-0.94	-1.01	-0.52	-1.20	-1.84	-0.43	-1.47	-1.12	-1.44	0.61	-0.13	-0.69	0.69	-0.30	0.65	-0.14	-0.89	-1.05	1.30
Armcx5	-1.18	-0.21	-1.56	0.27	-0.59	0.06	0.70	0.25	0.33	-0.23	1.26	-0.70	1.88	-0.46	0.63	-0.12	0.85	0.50	-0.18	1.17
Abcg2	-0.47	-0.11	-0.19	0.10	-0.74	0.87	0.76	0.68	1.05	-0.37	-0.25	1.00	1.44	0.54	0.65	0.35	-0.43	-0.46	0.94	2.89
Per2	-2.15	-0.78	-1.17	-0.93	-0.41	-0.50	-0.48	0.01	0.41	-0.55	0.83	0.01	0.26	0.67	1.00	0.97	1.28	0.91	1.08	0.87
Unc80	-1.31	-1.89	-0.61	-1.30	-0.85	0.09	-0.95	-0.69	-0.10	-0.05	1.23	0.26	0.92	0.29	1.06	-1.01	0.51	1.27	-1.61	-1.45
Ppargc1b	-0.97	-1.19	-0.34	-0.35	-1.07	-0.18	-0.92	-0.07	1.14	0.32	0.65	-1.02	-0.80	0.22	0.52	-1.08	1.77	0.56	-0.65	-0.95
Tex30	-0.44	-1.23	-0.76	-0.96	-1.22	-0.31	0.54	-0.60	0.71	-0.82	1.26	-0.18	-1.20	1.25	2.07	0.25	0.20	0.40	2.38	0.83
Lrif1	-1.25	-0.17	-1.30	0.07	0.07	0.28	0.19	-0.10	0.95	0.22	0.82	0.56	1.57	0.21	0.11	0.39	-1.14	-0.10	0.16	-0.47
Gm26782	-0.47	0.27	0.21	-0.96	-1.55	0.59	0.43	-0.13	0.26	0.47	0.86	0.40	1.53	0.76	0.85	0.20	-1.11	-0.47	0.87	0.13
Clk1	-1.42	-1.67	-1.62	-1.08	-0.84	-1.14	-0.50	-0.48	0.07	-0.48	0.02	0.02	-0.01	0.98	1.46	1.41	0.94	0.61	1.67	1.57
Id4	-1.16	-1.18	-0.91	-1.54	-1.73	-1.26	-0.13	0.04	-0.69	-0.40	0.00	-1.68	0.50	0.24	0.09	-0.12	0.15	0.36	2.17	1.56

Rpl8	-0.36	-1.05	-1.67	-1.64	-0.46	-0.03	-0.65	-0.61	0.01	0.17	-0.25	0.64	-0.97	-1.40	0.83	1.85	-1.06	1.20	-0.71	0.79
Lmbr1	-0.84	-0.65	-1.42	0.07	-0.23	0.19	0.14	-0.24	0.95	-0.21	0.81	0.11	1.54	-0.21	0.32	0.42	-0.37	1.04	-1.51	-0.89
Fbxo46	-0.57	-1.19	0.12	-1.06	-0.40	-0.10	0.33	0.33	0.42	-0.20	0.48	1.18	0.88	1.09	0.69	-0.10	-0.81	0.57	0.34	0.84
Ldhb	-0.77	-1.36	-0.77	-0.87	-0.94	-1.09	-0.34	0.24	0.33	-0.06	-0.22	0.59	0.19	-0.89	0.12	0.51	-0.89	-0.61	1.16	0.97
Tcerg1	-1.60	-1.61	-1.92	-1.83	-2.14	-1.22	-0.97	-1.35	-0.66	-1.68	0.91	1.33	0.36	0.42	0.41	0.33	0.62	0.78	-0.07	0.11
Echs1	-1.00	-1.19	-1.28	-1.01	-1.08	-0.31	-0.17	-0.55	-0.96	-0.61	-0.18	0.22	-0.48	1.08	-0.72	1.11	-0.03	-0.12	1.27	2.22
Mdh2	0.20	-0.27	-0.99	-1.64	-1.64	0.61	-0.39	0.48	3.09	1.64	0.55	1.48	-0.72	0.15	0.76	-0.99	-0.55	0.37	-1.09	-1.74
Rab33a	-0.40	-0.72	0.95	-0.84	-0.54	1.88	0.57	0.91	1.21	1.66	0.26	1.78	0.05	0.21	0.60	0.37	-1.15	-0.67	-1.49	-1.70
Hr	-1.60	-0.27	-0.59	-1.06	-0.74	0.25	1.02	0.47	0.73	0.73	2.22	0.53	1.00	0.55	-0.10	0.02	-0.57	-1.14	-1.47	-1.74
Tmem170b	-0.63	0.14	-1.04	0.25	0.07	1.80	0.72	1.63	1.87	0.10	0.14	-0.25	-0.78	0.74	-0.46	0.37	-0.58	0.91	-0.46	-1.15
Dcaf12l1	-0.25	-0.28	-1.05	-1.21	-0.10	0.84	-1.25	1.64	1.19	1.85	0.62	0.18	0.07	-0.62	0.93	-0.53	0.21	0.44	-1.63	-1.31
Fat4	-1.66	0.28	0.10	-0.51	0.74	1.50	1.23	1.21	0.82	0.87	0.04	0.89	-0.33	0.04	1.03	-0.31	0.72	1.13	-1.95	-2.64
Ccdc62	-0.78	-0.18	0.87	-0.35	-0.07	1.52	0.51	0.74	1.47	1.88	0.90	1.32	-0.68	-1.71	0.68	-0.29	-0.34	0.15	-0.97	-0.08
Hp1bp3	0.59	0.06	-0.34	-1.26	-1.20	1.52	-0.68	1.20	1.50	0.79	0.07	1.11	0.69	0.83	1.42	0.55	0.38	0.81	-1.73	-1.46
Zbtb11os1	0.38	-0.37	0.70	-1.11	0.35	1.45	1.02	-0.26	2.21	1.90	-0.29	0.96	-0.52	-1.26	0.68	0.28	-0.53	-0.16	-1.97	0.06
Gramd4	-0.18	-0.67	-0.90	0.20	-0.59	0.54	0.94	1.39	1.70	-0.40	0.31	-1.53	-1.45	0.05	0.62	-1.51	0.16	-1.58	-1.50	-0.53
Rab11fip4	-2.22	0.64	0.23	0.52	-0.56	1.05	1.39	0.36	0.52	1.44	0.84	0.99	-1.79	-0.86	0.12	1.08	-0.47	1.01	-0.84	-1.51
Atxn1l	-0.06	0.61	0.06	0.18	0.45	1.76	2.06	0.81	0.88	1.77	0.60	0.27	-0.82	-0.15	-0.74	-0.60	0.52	0.34	-0.78	-0.29
Ascc2	0.03	0.45	0.93	-0.24	0.21	0.37	1.33	2.56	1.75	1.37	0.13	0.23	-1.91	0.90	1.20	-0.40	-0.24	-0.19	0.53	-0.55
Dll1	-0.80	-2.11	-0.87	-0.36	0.71	1.31	0.99	-0.53	0.40	0.40	0.23	1.28	0.54	-1.09	0.37	0.98	-0.62	-0.04	-0.82	-1.96
Ccno	-0.71	0.25	0.86	-1.32	-1.06	2.00	0.79	0.92	-0.05	0.31	0.06	1.16	-1.51	0.45	0.92	0.08	-0.16	1.08	-0.85	-0.34
Herc3	-2.76	-0.69	-0.47	-0.85	-0.35	1.01	-0.97	-0.23	0.93	0.00	-0.34	-1.98	-0.22	0.84	-0.21	0.56	0.18	0.77	-2.51	-0.88
Ripk2	-0.40	-1.31	-0.01	1.16	-0.14	1.20	1.12	0.86	1.32	0.63	-0.05	0.10	0.14	0.52	0.29	0.38	-0.47	0.90	-3.25	-0.26
Hdgfrp3	-0.48	0.96	0.29	-0.87	0.50	1.98	0.69	0.02	1.46	2.10	0.74	-0.22	0.77	0.09	0.43	-0.43	-0.13	0.91	-1.58	-1.88
Pgrmc1	-0.84	0.33	-0.92	-0.23	-0.29	1.46	0.59	1.46	-0.43	0.70	1.02	-0.25	0.36	0.59	-0.46	-0.87	1.27	0.70	-1.69	-2.07
Fanca	0.83	-0.68	-0.17	-0.58	-0.17	1.08	0.67	1.86	0.53	0.76	-1.91	1.32	0.87	0.12	-0.65	0.23	0.04	0.85	-0.06	-1.25
Scamp5	-0.71	0.97	0.90	-0.21	-0.47	1.60	1.24	1.52	0.32	1.42	-0.33	0.69	-0.65	0.98	-0.22	0.06	-0.41	0.11	-2.01	-1.30
Wnt7a	0.10	-0.44	0.57	-0.01	-1.64	0.90	0.75	0.29	0.52	1.60	0.93	1.44	-1.28	-0.34	-0.39	-1.12	1.00	0.19	-1.00	-0.22
Hnrnpd	0.27	0.52	-1.50	-0.59	0.27	2.08	0.13	1.16	0.03	0.98	-0.49	-1.37	0.00	1.26	0.93	-0.90	0.51	0.57	-1.15	-1.22
Wnk3	0.66	0.28	1.57	-0.39	0.49	1.28	2.09	1.50	1.57	1.57	-0.50	-0.18	-1.74	0.20	0.03	-0.95	0.63	0.17	-0.53	-0.60
AU019559	-2.14	-0.31	0.17	-0.56	-0.20	0.54	0.50	0.84	-0.01	0.46	-0.63	-1.00	-1.69	-0.06	0.74	-1.41	-0.27	0.48	-1.19	-1.63
Capn6	0.58	0.37	0.59	0.10	-0.86	1.40	0.34	1.28	1.57	1.55	-0.32	1.12	-1.63	-0.04	-0.28	0.12	0.43	-1.80	-2.54	0.04
Zkscan16	-0.25	0.20	0.67	-0.01	0.53	1.40	1.21	1.02	1.42	1.40	0.51	0.42	0.00	-0.19	0.83	-0.68	0.59	0.83	-2.53	-2.22
Smco4	-0.26	1.28	-0.70	0.91	-0.65	1.70	1.24	0.67	1.27	0.93	-2.04	0.66	-2.89	-0.20	0.90	-0.44	0.01	-0.06	-1.49	-0.37
Cyp4x1	-0.55	0.24	-0.09	-0.48	-0.35	0.82	0.71	0.13	0.93	1.35	0.03	0.52	2.02	0.57	0.97	-0.46	1.85	0.18	-0.33	-3.03
Ipo5	0.08	1.21	-0.13	-0.07	0.89	0.66	2.24	1.46	0.89	1.89	0.66	0.56	0.30	0.43	-0.69	-0.80	0.97	0.68	-0.79	0.13
Acvr2a	-0.98	0.88	-0.33	0.13	0.51	1.71	0.96	0.84	0.99	0.85	-0.46	0.78	-0.84	0.38	0.48	-0.60	-0.59	0.51	-2.10	-1.22
Strn4	0.78	1.36	0.23	1.10	-0.26	1.53	1.52	1.27	0.96	2.97	-0.11	0.44	-0.84	0.11	-0.39	-0.98	-0.67	0.31	-1.35	-2.55
Dffb	-0.32	0.20	-0.70	-0.43	-1.21	-0.80	-0.18	0.90	1.19	1.47	-0.35	1.38	0.03	0.36	1.14	-1.56	0.14	0.34	-0.70	-2.24
Rab36	1.07	0.35	0.46	-0.26	0.95	0.66	2.45	2.01	0.82	1.65	0.90	-0.01	-0.70	0.09	1.31	-0.95	-0.70	-1.13	-1.18	-1.63
Slc25a27	-1.73	-1.22	0.09	-0.07	0.10	-0.35	1.27	0.92	0.10	0.24	0.37	0.87	-0.98	1.18	1.38	-1.55	1.00	-0.20	0.74	-2.49
Gm10523	0.13	0.39	-0.96	-0.40	-0.39	1.20	1.01	1.51	0.41	-0.37	0.19	0.17	-0.39	-0.66	1.50	0.12	-0.95	-1.48	-1.93	-0.35
Ptprs	0.75	-0.06	0.24	0.36	0.51	1.95	1.45	1.35	1.04	0.97	-0.40	-0.06	-0.21	0.02	-0.36	-2.03	-0.06	0.00	-1.16	-0.77
Tab3	-0.10	1.67	-0.62	-0.20	-0.09	1.55	0.76	0.83	0.51	1.89	0.70	1.90	-0.38	1.54	0.14	1.98	-0.64	-0.13	-1.75	-1.79
Astn1	0.64	0.72	0.68	0.72	0.64	1.94	0.67	1.53	2.05	1.99	0.02	0.83	-1.01	0.01	0.19	-1.32	-0.41	-0.60	-2.31	-1.60
Zfp781	0.39	-0.32	0.01	0.35	-1.01	0.85	0.54	0.64	1.17	0.88	-0.35	-0.26	-0.04	2.11	0.09	-1.49	0.49	-0.73	-0.92	-0.48
Arnt2	-1.07	-0.14	-0.10	0.06	0.17	0.67	0.64	0.45	0.91	0.89	0.57	-0.28	-0.10	0.09	0.77	-0.10	0.42	0.98	0.48	-0.86
Rwdd2a	-0.07	-0.13	-0.38	-0.76	-1.29	0.35	0.01	0.03	1.22	0.30	1.10	0.71	1.18	0.71	0.11	0.05	0.45	0.87	-1.10	-2.06
Resp18	0.31	0.31	0.06	0.67	-0.39	1.47	1.31	0.89	0.17	1.52	0.69	-0.22	-0.48	-0.48	0.44	-0.04	0.54	0.02	-1.66	-1.32
Ddx27	-0.26	1.22	-0.07	0.34	-0.28	1.20	0.93	1.90	0.69	0.62	-0.37	1.21	-0.38	-0.35	1.21	-0.33	0.94	0.28	-1.14	-0.52
Mef2d	-0.42	-0.06	0.73	-0.36	0.77	1.55	0.40	0.66	1.17	1.23	0.22	-0.51	-1.02	-0.73	0.11	-1.84	-0.55	-0.24	-4.17	-1.22
Elfn2	0.78	-0.53	-0.20	0.20	0.90	1.41	1.99	0.34	1.12	0.62	0.15	0.40	-0.69	-0.96	-0.27	-2.33	1.07	0.50	-1.83	-0.72
Pcbp2	1.38	-0.07	-0.22	-0.54	0.58	0.61	0.97	1.67	1.25	0.97	0.16	0.10	-0.48	-0.22	0.72	0.34	-1.32	-0.17	-2.35	-1.26
Frs2	0.69	0.17	0.61	1.09	0.18	1.61	1.32	0.71	2.06	1.39	-0.17	-1.24	-1.24	0.54	-0.42	0.55	1.56	1.42	-0.80	-1.00
St6gal2	-0.63	0.72	0.09	0.84	0.75	1.47	0.85	1.27	1.42	1.08	0.56	-0.34	-0.20	0.66	-0.11	-0.27	0.63	0.95	-1.63	-1.21
Smarcd1	0.34	-0.42	0.83	0.95	0.89	1.63	1.32	0.85	1.37	1.70	0.87	-0.15	-2.31	0.28	-1.25	-1.35	0.10	0.04	-2.02	0.15
Pi4k2b	0.21	-0.71	-0.45	-0.18	0.35	0.12	1.19	0.58	0.81	0.79	0.78	-0.48	-2.63	0.16	0.77	0.69	1.34	-0.71	-3.68	0.50
Rapgefl1	0.42	0.85	0.90	-0.51	0.56	1.74	0.62	1.43	1.16	1.52	-1.49	0.17	0.10	0.42	0.29	-1.35	0.39	1.09	-1.15	-2.55
Slitrk5	1.12	-0.43	0.19	0.12	1.00	0.85	1.60	1.14	1.82	0.85	1.40	-0.45	0.12	1.07	0.38	-0.74	-1.38	-0.47	-0.76	-2.08
Iqca	-0.74	0.42	0.60	0.27	-0.16	1.61	0.32	1.56	0.69	0.47	-1.46	-2.21	-0.76	-0.07	0.10	1.14	0.09	-0.08	-1.79	-1.25
Gm9899	0.71	0.54	1.20	-0.63	0.48	1.94	0.95	1.34	1.03	1.29	0.50	0.12	-1.16	-2.14	-0.67	-1.43	-0.24	1.10	-1.80	-1.05
Pcdhb17	-0.32	0.74	-0.34	0.35	0.38	1.50	0.82	1.22	0.81	0.70	-1.20	1.62	-0.61	1.08	-1.68	-1.86	-0.64	0.56	-1.24	-1.05
Cul3	0.07	0.60	0.57	-0.37	-0.22	1.49	0.96	0.53	0.57	1.33	0.65	-0.63	0.06	1.04	-0.18	0.10	0.43	0.70	0.72	-1.97
Rbbp6	0.44	0.68	0.44	0.09	-1.31	0.87	0.75	1.05	0.87	0.98	0.70	0.71	0.48	-0.52	0.09	0.35	0.33	0.52	-3.45	-0.16
Lrba	0.38	-0.25	-0.21	-0.79	-0.47	0.78	0.81	0.52	0.10	0.63	1.07	0.22	0.10	0.18	1.21	1.88	-0.35	1.47	-1.70	-0.99
Akap11	1.04	0.03	1.52	0.54	0.62	2.05	1.33	1.10	1.38	2.01	-0.26	0.05	-0.31	0.07	0.27	-0.49	0.15	0.81	-1.52	-2.04
Nlgn1	-0.67	-0.53	-1.07	-1.48	-0.86	0.62	0.66	-0.67	-0.95	-0.17	1.35	1.51	0.61	1.44	0.62	-0.64	-0.93	0.33	-0.83	-3.11
Ldlr	-0.72	-0.65	-0.44	-0.59	0.17	0.49	0.35	0.59	0.23	0.20	0.12	0.59	0.82	0.48	0.22	-1.08	0.95	0.98	-3.28	-2.00
AI451458	0.12	0.37	-0.35	-0.54	0.05	0.85	1.11	0.37	1.19	0.17	-2.48	0.50	0.62	-1.72	1.22	0.30	0.78	-0.72	-0.50	-2.87
Spry1	0.09	0.94	0.07	-0.13	0.98	1.51	1.00	1.00	1.52	0.95	-0.62	1.40	-0.14	0.19	0.02	0.17	-0.52	-1.78	-2.42	0.60
B3gat1	0.77	0.74	0.74	0.91	0.68	1.87	1.17	1.29	1.54	1.97	-0.30	-0.72	-0.09	-0.29	-0.38	-1.68	0.02	0.38	-1.63	-2.28
Gls	0.54	0.77	0.69	0.04	0.03	1.27	0.57	1.02	1.01	2.18	0.77	0.52	-0.37	0.59	0.11	-1.46	0.86	0.27	-2.48	-2.32
Ly6h	0.45	0.81	1.19	-0.10	0.10	1.23	1.06	0.74	1.66	1.76	0.10	0.50	-1.76	-0.72	-0.02	0.02	-0.49	-0.52	-0.99	-1.87
Actg1	1.42	0.54	1.05	0.84	0.75	2.08	0.84	1.70	1.85	2.08	-0.61	-0.33	-0.06	0.54	-0.71	-0.91	-0.79	0.65	-1.58	-1.27
Ctps2	-0.84	0.31	0.11	-0.05	-0.37	0.72	-0.21	1.24	0.49	0.80	0.74	0.10	-1.59	0.06	1.06	-0.37	0.05	0.57	0.91	-2.99
Dyx1c1	0.13	-1.02	-0.02	-0.34	0.35	0.89	0.54	0.78	0.13	0.58	0.06	-1.06	0.77	0.62	0.38	-0.42	-0.35	-0.15	-2.61	0.06
Fam132a	0.56	0.20	0.35	-0.33	-0.49	0.59	0.34	1.20	0.54	1.43	-0.04	0.97	0.44	0.42	0.73	0.02	1.54	0.87	-0.33	-2.76
Pnck	0.41	0.90	0.80	-0.40	0.10	1.41	1.54	0.62	1.08	0.94	0.76	0.30	-1.37	-0.03	0.78	-0.84	-1.34	-0.23	-1.77	-1.83
Smarcal1	-0.27	0.02	0.43	0.37	1.07	1.06	1.06	0.83	1.27	1.10	1.35	-0.31	-0.37	0.02	-0.10	-0.78	-0.81	-0.58	-2.66	-2.68
Dnajc30	1.10	0.38	1.21	0.56	0.75	1.32	1.93	1.31	1.87	1.26	-0.38	0.87	-0.38	0.06	-0.54	-0.96	-0.41	-0.05	-1.05	-1.72
Tceal3	-0.46	-1.20	-0.50	-0.83	-0.31	-0.27	-0.12	0.23	0.74	-0.33	0.12	1.14	-0.69	0.25	0.23	-0.94	-0.17	0.23	-1.09	-2.20

Nsdhl	0.51	0.62	0.40	0.58	0.73	1.54	0.59	1.20	1.64	1.30	-0.45	0.24	-0.03	0.24	0.10	-1.49	0.48	-0.31	-2.43	-2.89
Mrps31	0.62	-0.43	0.24	-0.17	-0.25	0.38	0.68	1.03	0.71	0.50	-0.02	0.77	0.31	0.31	-1.23	0.40	0.52	-0.31	0.09	-4.78
Efnb2	0.14	-0.17	0.10	0.49	0.24	0.85	0.58	1.05	0.30	1.24	0.24	1.17	0.28	0.32	0.61	0.51	0.69	-0.23	-1.81	-1.65
Scly	1.11	0.51	0.49	0.43	0.95	1.26	0.90	1.68	1.38	1.44	-0.79	0.25	0.03	-0.31	0.19	-0.44	-1.89	-0.73	-1.17	-2.61

Supplementary Table 2. List of genes exclusively downregulated in the hippocampus of P301S mice at 3 months, compared to 3-month-old WT mice.

Gene symbol	WT_1_3M	WT_2_3M	WT_3_3M	WT_4_3M	WT_5_3M	Tau_1_3M	Tau_2_3M	Tau_3_3M	Tau_4_3M	Tau_5_3M	WT_1_9M	WT_2_9M	WT_3_9M	WT_4_9M	WT_5_9M	Tau_1_9M	Tau_2_9M	Tau_3_9M	Tau_4_9M	Tau_5_9M
Sdf2l1	1.96	0.73	1.60	1.58	1.31	-0.46	-0.26	0.12	-0.82	-0.75	-0.79	0.70	-0.76	-0.29	-1.38	-0.50	-0.97	-2.58	-0.30	0.66
Manf	1.32	0.15	1.99	1.98	1.62	-0.82	0.50	-0.40	-0.31	-1.17	-0.60	1.34	-0.84	-0.32	-0.35	-0.57	-1.36	-0.88	0.30	-0.26
Bok	2.18	1.16	1.16	1.08	1.05	-1.09	0.28	-0.32	-1.80	0.84	-0.19	0.53	1.21	-0.49	-0.39	-0.07	-0.15	-0.99	-0.35	0.56
Pdia4	1.14	1.03	1.84	0.60	0.46	-1.14	0.58	-0.67	-1.09	-0.92	-0.89	-0.61	-0.83	-0.71	-0.71	1.52	-1.35	-1.44	0.70	0.24
Ypel5	0.30	0.32	-0.04	2.23	1.17	-0.93	0.94	-0.41	-2.32	-0.01	-1.10	0.25	-1.13	0.40	-0.95	-0.86	0.86	-1.73	0.24	0.76
Rasl12	1.10	1.21	1.04	1.24	0.45	-0.89	1.13	-0.17	-0.56	-1.15	0.86	0.44	0.35	-0.07	-0.54	0.09	-1.36	-0.17	-0.80	0.62
Ubash3b	1.43	-0.37	0.95	1.75	0.08	-0.57	-1.35	-0.46	0.13	-0.56	0.26	-0.61	0.16	-1.36	-0.11	-1.18	-0.77	0.94	1.48	0.51
Zfp983	0.74	0.76	0.38	0.54	1.06	-1.20	-0.35	-2.19	1.20	-0.60	-0.95	-1.15	0.18	-2.61	-0.13	0.53	-0.81	-0.20	0.25	-0.14
Ube2z	0.73	2.01	0.08	2.17	1.21	-0.44	-0.11	1.40	-0.74	-0.55	-0.12	-1.62	1.02	-0.18	-0.21	-0.41	0.04	-1.80	0.81	0.91
Capzb	1.24	1.84	0.97	1.43	1.23	-0.17	0.47	1.28	-0.51	-0.82	-0.01	0.07	-0.73	-0.21	-0.13	0.49	1.05	-1.57	-0.53	0.09
Snx12	1.42	1.90	1.11	1.86	0.66	-0.37	1.32	0.91	-1.04	-0.27	0.30	-0.19	0.12	-0.51	0.29	1.28	-0.46	0.26	0.84	-0.20
Rbm28	0.56	0.93	1.26	-0.10	0.16	0.82	-0.79	-0.46	-1.25	-1.79	-0.97	0.21	-0.90	0.60	-0.79	0.04	-0.29	-1.17	-1.36	0.49
Faap24	1.42	1.05	-0.07	1.20	0.78	-0.94	0.74	-0.62	0.00	-1.09	-2.03	0.95	0.21	1.26	-1.45	0.28	-0.76	-0.28	0.56	-0.37
Scarb1	0.11	1.59	1.44	2.12	1.17	0.08	0.08	-1.32	1.31	0.21	0.23	-1.12	-0.57	-2.45	-2.16	0.20	-1.61	-0.19	-0.79	-1.80
Sema5b	1.50	1.07	-0.35	1.08	0.66	0.86	-1.83	0.86	-1.28	-0.64	-1.24	-0.48	-1.16	-0.81	-1.23	-0.13	0.77	0.26	0.48	0.56
Qsox1	1.11	1.14	0.73	0.18	0.77	-0.31	-0.06	-1.44	-0.35	0.16	0.10	-0.35	0.26	0.57	-0.57	-1.82	1.04	-0.55	0.83	1.21
H2afv	0.93	1.06	0.51	0.64	0.17	-0.42	0.04	-0.79	-0.92	-0.49	-0.34	-0.70	2.28	-0.11	-0.13	-1.25	0.03	-1.75	0.08	0.84
Uap1	-0.44	1.37	1.36	1.91	1.58	-0.83	0.34	0.37	0.09	-0.04	0.26	0.73	-0.83	-2.05	-0.46	1.47	1.39	-1.50	1.07	-0.27
Perp	1.42	1.17	0.70	2.25	0.63	-0.33	-0.64	-0.04	1.10	0.29	-0.50	-1.61	0.29	-1.89	0.02	-0.53	0.20	-0.44	-0.15	-0.59
Cox7c	0.35	-0.98	0.75	1.74	1.81	-1.02	-0.18	-0.38	0.54	-1.09	0.75	-0.07	-0.73	-1.11	0.16	-0.24	0.37	0.30	0.44	1.35
Elfn1	1.40	0.93	1.38	2.12	1.10	0.64	-0.12	0.60	0.05	-0.02	0.60	-0.87	0.28	0.02	0.59	-0.24	-0.20	-0.40	0.79	0.74
Vti1b	1.43	0.65	-0.03	0.44	0.81	-0.21	-0.32	-0.56	-1.00	-0.35	-0.67	-0.51	-1.67	0.10	-0.48	-0.13	-1.38	-2.10	-0.71	-0.03
Capn3	-0.15	0.95	-0.13	0.50	1.68	-0.92	-0.34	0.08	-0.13	-1.52	-0.70	0.30	-1.28	-0.32	-0.11	-0.44	0.21	-0.58	-0.93	0.40
Eed	1.52	0.95	0.52	0.60	-0.39	0.52	-1.42	-0.74	-0.48	-0.24	0.18	0.84	-0.23	-0.29	-3.23	0.70	-0.47	-1.09	0.89	-0.72
Mns1	1.02	0.62	0.60	0.63	1.13	-1.36	0.92	-0.77	-1.18	0.87	-1.35	0.73	0.61	-1.32	1.31	-1.12	0.34	-1.18	0.03	1.35
Kcns3	0.99	1.51	1.05	0.86	1.70	-0.20	0.90	-0.43	0.27	0.08	0.58	-2.30	-0.39	1.19	0.84	-1.15	1.05	0.43	0.62	0.46
Ppp2r5a	1.62	1.19	1.00	1.25	1.85	0.29	0.77	0.36	-0.94	0.95	-0.60	-0.72	-0.46	-0.54	-1.48	0.32	-0.19	-2.67	1.10	1.56
Rngtt	1.57	0.95	0.97	1.36	1.35	-0.27	0.28	1.06	-0.58	0.26	-0.30	1.12	0.29	0.09	-0.57	-0.63	-0.65	-1.72	-0.16	0.35
Nudc	1.34	2.24	1.66	1.39	1.19	-0.65	-0.18	1.00	2.01	0.17	-1.24	-0.35	-1.26	0.01	0.33	-0.46	-0.59	-1.06	-1.04	0.77
Mrpl52	1.29	1.34	0.90	0.74	-1.09	-1.31	0.34	-0.99	-0.49	0.27	0.13	1.11	-1.09	0.76	-1.27	-0.30	0.51	-0.68	0.08	0.53
Sec24a	0.73	0.31	-0.26	0.80	0.64	-1.49	0.16	0.13	-1.65	-0.28	0.64	-1.27	-0.99	-1.04	0.21	0.23	0.70	-0.76	-0.43	0.58
Apln	1.51	1.44	0.15	-0.14	0.63	-0.40	0.15	0.19	-0.52	-1.09	0.77	-1.92	-0.90	-1.34	0.85	-1.26	-0.75	-1.61	-1.01	0.59
Yipf6	0.29	0.61	0.33	1.12	0.10	-0.13	-1.07	0.68	-2.12	-0.16	-1.41	-2.40	0.86	0.24	-1.22	-1.90	0.06	-0.32	-0.09	0.45
Cnpy4	2.29	0.68	-0.75	0.60	1.00	-0.73	-0.22	0.04	-0.35	-0.12	-1.19	0.59	-2.21	0.13	1.47	0.61	0.15	-0.98	0.73	-0.10
Lman2	1.05	0.35	1.39	0.85	0.93	-0.04	-0.24	-0.66	0.52	-0.21	-1.12	-1.34	0.55	0.80	-1.36	-0.26	-0.86	-0.02	0.09	0.27
Timmdc1	1.36	0.98	0.14	1.05	0.28	-0.50	1.37	-0.25	-0.83	-1.08	0.10	-0.79	-0.54	-0.97	-1.99	-1.10	0.67	-2.51	-0.93	-1.17
Eif4ebp3	1.10	-0.99	-0.56	1.11	0.87	0.75	-0.10	-0.51	1.32	1.74	0.71	-0.64	-1.37	0.52	-0.64	0.70	0.78	-0.21	1.11	2.06
St5	1.47	2.24	2.03	1.51	1.40	0.59	0.42	0.89	1.07	0.64	-0.50	-0.35	-0.43	-0.93	-1.66	0.17	0.30	-0.56	-0.26	-1.02
Galt	0.45	0.80	1.36	1.07	1.58	-0.08	0.30	0.74	-0.29	-0.40	-1.92	-0.12	-0.98	0.32	0.87	0.20	0.20	-2.22	-1.26	-0.39
Ryk	0.52	1.48	0.88	1.43	1.84	0.78	0.42	0.76	0.30	-1.07	0.05	-1.01	-1.10	-0.41	-1.50	0.61	0.33	-1.18	0.57	1.33
Vps18	0.95	1.14	1.39	0.13	0.11	-0.20	0.59	-0.07	-0.54	-1.00	0.48	0.78	-2.24	-2.58	-0.08	0.07	0.01	-0.92	1.16	-0.91
Slc25a26	1.84	0.55	0.57	1.12	0.69	-0.34	-0.15	-0.83	0.13	1.04	-0.80	0.01	0.59	0.44	-3.96	-0.27	0.47	-1.50	0.07	-0.69
Rps6ka1	0.16	0.99	1.06	1.71	0.41	-0.95	-0.37	0.58	-0.26	0.42	0.06	-1.02	0.18	-0.98	-0.72	1.22	-0.90	0.53	1.56	1.65
Creb3l2	1.20	0.88	0.42	0.79	0.00	-0.44	0.33	-1.60	0.40	-0.29	-1.87	-0.61	-0.27	-1.47	-0.43	0.58	-0.04	-1.32	2.28	2.26
Inhba	1.28	0.32	0.36	1.25	0.64	-0.79	-0.13	-0.12	0.25	-0.22	0.56	-0.05	-0.60	-0.34	-0.59	1.07	-1.99	0.28	0.70	0.54
Slc16a9	0.83	0.50	0.36	0.98	-0.38	0.35	-0.60	-0.02	-0.85	-1.34	0.32	0.61	0.12	-0.23	-0.22	-1.01	-1.02	-0.77	0.19	0.30
Jtb	1.77	0.78	0.24	0.22	-0.09	-0.16	-0.52	-0.63	-0.75	0.21	-1.10	-1.84	-2.12	0.61	-0.26	0.86	0.39	0.33	0.51	2.11
Gcc1	2.30	0.52	2.31	0.42	1.57	1.06	0.55	0.33	-0.18	0.62	-0.10	-0.34	-1.75	0.25	1.00	0.06	-0.21	-0.03	2.08	-1.17
Eri1	1.38	0.78	1.16	-0.18	1.30	-0.34	0.18	-0.30	0.02	0.13	-0.35	0.16	-1.27	0.48	0.92	1.49	0.35	-0.81	0.14	1.33
Lgals4	0.66	0.18	0.64	0.43	0.95	0.09	-0.73	-1.60	0.54	-0.09	0.62	-0.09	-0.12	-0.25	-0.69	-0.78	0.13	-1.99	0.04	1.70
Srxn1	2.23	0.95	1.42	1.31	1.21	0.55	1.33	0.16	-0.13	0.59	0.55	1.23	-1.06	-0.91	-0.95	0.07	-2.24	-0.43	0.18	0.94
Zfp27	0.42	-0.28	1.38	1.21	1.03	-0.96	0.98	-0.30	-0.06	-0.49	0.25	0.44	-1.21	-0.25	0.01	-0.25	0.00	0.46	0.26	0.51
Cenpp	1.00	1.44	1.53	0.27	1.15	-0.64	0.21	0.01	0.52	0.73	1.36	-0.25	-0.26	0.21	0.56	0.36	-1.12	-0.18	0.27	0.90
Wbscr22	1.15	0.90	1.00	0.34	1.91	-0.59	0.73	0.55	-0.64	0.74	-0.03	-0.36	-0.12	-1.04	-0.51	-1.01	-1.43	-1.11	1.16	0.84
Pycrl	1.86	1.36	1.75	1.02	0.64	-0.24	0.58	1.13	0.43	0.21	-2.39	-1.36	-0.01	-0.13	-0.69	-0.84	0.54	0.70	-1.63	1.08
Commd4	0.31	0.57	0.58	1.14	1.14	-0.55	0.63	-0.57	-0.64	0.37	-0.21	0.75	-1.71	-1.29	0.83	-0.75	0.78	-1.34	-0.24	0.58
Dhodh	0.91	1.17	1.21	0.76	1.33	-0.29	0.64	0.64	-0.03	0.00	0.06	0.08	2.20	0.56	0.78	-1.27	-0.47	-0.12	-0.02	0.84
Mdc1	0.18	1.60	0.71	1.57	0.22	-0.14	-0.56	-0.17	0.36	0.40	-0.67	-1.15	-0.19	-2.80	0.28	0.14	-0.10	-0.42	1.52	1.14
Usp19	1.97	0.50	2.39	1.75	1.64	0.60	1.34	0.57	0.81	0.57	-0.23	-0.45	-1.35	-0.27	-2.39	-1.49	0.20	-1.00	-0.55	-0.15
Fam101b	1.44	1.08	1.01	1.01	1.13	0.43	0.28	-0.84	1.32	0.20	-0.35	-0.84	-0.22	0.38	1.10	0.25	-1.05	-2.86	-0.97	0.64
Ctla2a	0.61	0.84	-0.35	1.58	0.93	-0.85	0.09	0.24	0.35	-0.50	-0.82	0.00	-1.29	0.23	0.01	0.81	0.18	-2.91	2.30	0.65
Rnf145	2.09	1.94	2.01	1.82	0.54	1.23	0.95	1.27	0.31	0.41	0.37	0.14	-0.65	-0.85	0.10	0.80	-0.32	0.05	-0.12	0.11
Phkg2	1.50	1.39	2.45	1.61	1.65	0.98	1.03	1.70	-0.07	0.74	-0.31	-0.95	-0.41	-0.06	-0.86	-0.46	-0.81	-0.42	0.00	-0.10
Cth	0.63	0.12	0.23	0.56	0.92	-1.17	0.58	-0.94	-0.08	-0.11	-0.87	0.71	1.17	-2.24	0.19	-2.15	0.43	-1.64	1.22	0.20
Mpzl2	0.76	1.48	0.75	1.46	0.60	0.50	0.10	0.22	-0.84	0.87	0.27	0.78	0.42	-0.38	0.71	-0.73	-0.03	-2.35	0.66	-0.14
Fam195a	1.04	1.66	2.04	0.43	0.70	-0.10	0.57	0.57	-0.09	0.81	-0.07	1.25	-1.54	-0.89	-0.55	-0.13	-0.55	-0.61	-1.21	1.27
Twf2	1.86	2.06	0.89	1.55	0.80	0.75	0.64	0.23	0.24	1.20	-0.85	-2.03	0.30	-0.65	-0.80	0.78	0.62	-0.83	0.37	-0.22
Mthfd1	1.31	1.42	1.32	0.55	0.95	0.51	1.07	-0.31	0.38	-0.16	0.13	-1.94	1.21	-1.80	-0.49	-0.88	0.38	0.75	1.77	1.53
Plin4	0.96	0.40	0.90	0.41	0.74	0.01	-0.02	-0.65	-0.15	0.17	0.49	0.54	-0.46	0.26	0.28	-0.62	0.61	-2.53	1.19	0.57
Mapk4	1.86	0.76	1.71	1.37	1.26	0.43	0.68	0.80	1.08	-0.07	-0.69	-1.34	-0.92	-0.52	0.84	0.11	-2.52	-0.71	-1.66	-0.40
Stac2	1.64	1.71	2.32	1.07	1.77	0.85	1.17	0.37	0.93	1.17	-0.89	-0.13	0.39	-0.38	-0.41	-0.20	-0.66	-1.28	-0.96	-0.11
Stmn4	0.75	1.05	0.14	0.57	0.06	-0.24	-0.08	-0.28	-0.26	-0.55	0.07	-0.80	-0.61	-0.75	-0.32	0.09	-0.29	-0.63	0.86	1.04
Gprc5b	0.36	1.07	1.62	0.83	1.17	0.63	-0.04	-0.17	0.20	0.47	-1.51	-1.15	-0.74	-0.93	-0.48	-0.24	-0.69	-0.40	0.88	0.35
Plekhg2	0.48	1.24	1.23	0.72	1.57	0.27	-0.47	0.32	1.04	0.18	-0.09	-0.15	0.30	-0.03	0.88	-0.05	0.59	0.15	0.23	1.78
Gng5	-0.06	0.72	0.84	0.00	-0.17	-0.84	-0.29	-1.24	-0.29	0.13	0.73	0.54	0.04	0.93	-1.14	0.76	-0.20	-3.94	0.39	1.04
Nadk	1.16	0.20	0.36	1.42	0.59	-0.45	-0.49	0.41	0.32	0.15	-0.47	-0.21	-2.30	-1.16	0.30	-0.61	-0.77	0.27	1.86	0.93
Six3	0.94	0.21	1.07	0.64	0.92	-0.15	-0.37	-0.18	0.25	0.49	0.40	-1.49	0.48	0.33	0.10	-0.61	0.99	0.49	-0.16	0.77
Myl6	-1.31	1.67	1.41	0.80	0.86	0.10	0.51	1.21	1.19	0.46	0.95	0.63	-0.34	0.82	-1.99	0.02	-0.43	-0.80	-0.17	-0.02
Slc15a4	1.29	0.97	0.53	1.12	0.39	-0.32	0.17	-0.07	0.43	0.37	-1.97	-0.28	-1.10	0.04	0.16	1.01	-1.05	-0.42	2.62	1.17
Nbl1	1.51	1.81	1.36	1.37	1.34	1.21	1.25	0.26	0.21	0.76	-0.78	-0.12	-0.68	-0.01	0.56	-1.05	-0.44	-1.02	0.05	0.35

Yrdc	0.32	0.43	0.62	0.30	0.42	-0.63	-0.16	-0.63	-0.05	-0.06	-1.68	-0.24	-0.49	-0.54	0.03	-0.35	-0.32	-1.07	-2.32	0.43
Plpp7	0.73	1.59	1.74	1.56	1.72	0.57	0.91	0.95	0.92	0.40	-1.38	-0.17	-0.27	-0.44	-2.19	-1.13	0.67	0.21	0.08	1.56
Pomk	1.85	1.80	1.84	1.22	1.45	0.93	0.70	1.21	0.87	0.99	0.40	-0.28	0.40	-1.20	0.63	0.64	0.22	-0.10	-0.87	0.31
Cyp39a1	0.39	1.06	0.89	0.76	1.11	-0.29	-0.01	0.02	0.94	0.08	-0.55	-1.89	-0.82	0.40	0.45	1.29	-1.09	-0.10	1.26	0.10
Kcnn1	0.59	1.15	0.61	1.46	0.83	0.49	-0.36	0.23	0.77	0.08	-1.00	0.47	-0.20	-1.32	-0.12	-0.35	0.49	-1.28	-1.26	-0.33
Lamc1	1.18	0.98	1.18	1.62	1.49	-0.08	0.80	0.98	0.38	1.00	-1.25	-1.67	-1.05	-1.31	-0.66	0.00	0.93	-0.26	0.88	-0.48
Hspb1	1.22	0.66	1.02	0.97	0.67	0.36	0.33	0.39	0.00	0.50	-0.66	0.01	-1.11	-0.54	-0.50	0.58	-0.69	-0.29	1.06	1.10
Zfas1	-0.68	0.82	0.18	0.70	0.92	-1.81	-1.02	-0.90	-3.66	-1.50	0.34	-0.76	0.70	0.27	0.22	0.28	0.68	-0.23	1.77	-0.03
Hist3h2a	0.97	0.78	0.26	1.82	0.75	-1.82	0.40	-1.24	-2.22	-0.74	0.52	0.04	0.08	0.66	-0.44	0.43	0.28	-1.74	0.26	0.71
Dleu2	1.60	0.30	0.40	0.81	0.68	-2.81	0.05	-1.01	-0.93	-1.46	-1.31	0.30	1.22	0.56	0.93	0.83	-1.13	-0.62	0.24	0.27
Rpl17	-1.67	0.42	-1.58	-0.10	-1.76	-1.38	-1.22	-1.17	-2.04	-1.99	-0.25	0.39	-0.88	-0.40	0.64	1.17	-0.19	-0.15	1.46	1.20
Bod1	0.96	0.87	0.74	1.22	0.62	-1.52	1.23	-0.53	-3.23	-0.34	-0.63	0.96	-0.72	0.25	0.33	0.05	-0.02	-0.31	0.09	0.43
Pdia3	0.78	-0.44	0.65	0.75	0.67	-1.49	0.73	-1.69	-2.08	-1.67	-0.85	0.55	-1.32	-0.44	-1.36	1.10	-0.40	-0.58	0.70	0.47
Mrpl48	0.69	1.12	0.09	-1.70	-0.45	-0.82	-0.46	-0.02	1.46	0.95	0.77	1.68	-1.86	0.54	0.37	0.78	0.48	-0.53	0.60	0.58
Cx3cr1	0.48	-0.08	0.61	0.23	-0.62	0.34	-1.93	-3.18	-3.19	0.11	-1.31	0.65	-1.17	0.07	0.70	0.65	0.17	0.46	0.84	1.12
Clta	-0.47	1.43	0.89	1.53	-0.41	-1.36	0.09	0.16	-0.98	-3.23	0.05	-0.65	-0.28	0.10	0.43	1.53	1.02	0.24	1.23	1.50
Cyct	1.07	-0.32	0.02	0.33	0.82	-0.79	0.29	-4.07	-2.16	0.37	0.65	1.15	0.43	0.61	-0.26	-0.70	-0.59	0.51	0.08	-1.21
Gja1	0.25	0.16	-0.01	-0.04	0.50	-1.48	-1.02	-0.41	-0.02	-4.46	0.06	0.10	-0.72	0.41	0.42	-0.89	-0.32	-0.70	0.87	0.43
Cct5	0.03	0.29	1.49	1.46	0.60	-1.02	0.64	0.01	-3.25	-0.66	0.69	0.48	-1.48	1.30	0.46	0.30	0.18	-0.04	-0.28	1.33
Sparcl1	0.50	0.82	2.14	0.56	0.34	-0.11	0.10	0.64	-2.16	-2.21	0.82	-0.91	0.39	0.45	0.90	-1.73	-0.04	1.35	0.90	0.75
Sfmbt2	1.26	0.40	0.47	0.54	0.47	-1.30	-1.31	-1.11	-1.50	0.33	-0.41	-2.05	0.29	-1.02	0.40	1.18	1.77	-0.15	-0.43	-0.06
Bri3	-0.20	0.74	0.23	1.27	-0.69	-0.90	-1.25	-0.13	-2.36	-1.87	-0.14	1.15	-0.61	0.67	-0.32	0.36	-0.55	0.43	0.00	1.56
Sec13	-0.64	1.28	0.68	0.14	0.50	-0.25	-0.59	-0.72	-3.13	-1.18	-0.32	-0.35	-0.99	-0.63	0.50	0.16	-0.14	1.12	0.12	1.21
Chordc1	-0.27	-0.43	0.24	0.40	-0.47	-2.45	-0.74	-1.73	-1.97	-1.41	-0.04	-0.13	-0.19	0.50	-0.20	0.22	0.54	-0.83	-0.03	0.50
N4bp2	-0.08	0.39	0.08	0.09	0.08	-0.44	-0.39	-2.52	-1.11	-2.48	-0.11	0.37	-1.06	0.07	1.45	-0.04	-0.19	-0.52	0.04	0.38
Foxq1	0.66	1.30	0.88	-0.18	0.40	-0.04	-0.15	-0.99	-1.45	-1.81	0.06	-0.03	-1.00	0.67	-0.32	0.37	0.11	0.12	0.21	0.24
Rho	0.77	-0.89	1.26	1.03	0.36	0.35	-0.72	-2.31	-1.47	-0.75	-1.71	-0.71	0.29	0.34	0.09	1.76	-0.95	-1.11	0.76	0.19
Mylip	1.49	1.05	-0.14	0.52	1.64	0.19	0.95	-1.31	-1.85	-0.80	0.47	0.68	-0.89	0.32	0.20	1.39	-0.42	-0.02	0.54	1.30
Phospho	0.84	0.89	0.46	0.48	0.00	-0.45	-0.82	-1.26	-1.86	-0.26	-0.48	-0.09	1.27	0.26	0.79	-1.01	-0.78	2.52	0.90	-0.35
Pla2g4b	0.41	0.63	0.63	0.82	0.61	-0.16	-1.44	0.67	-3.41	0.16	-0.24	0.08	-0.81	-1.75	0.05	-0.07	0.35	0.69	0.25	-0.05
Dock8	-0.11	0.06	0.38	0.63	1.59	-0.77	-0.59	-2.61	0.28	-0.94	0.08	0.33	-0.68	-0.38	-0.37	0.45	-0.66	-0.17	2.03	0.76
Eps15	0.95	-0.10	0.67	0.89	-0.21	-0.02	-0.72	-1.97	-1.58	-0.58	1.29	0.52	-2.48	-0.61	0.37	-0.57	-0.93	-0.31	2.16	1.19
Fam46c	-0.47	0.51	0.05	0.28	0.07	-1.57	-0.46	-0.15	-0.31	-4.06	0.47	0.39	0.40	-0.89	-0.52	1.44	0.41	0.64	1.72	1.69
Mettl3	0.52	-0.30	1.12	-0.20	0.59	-2.32	-1.42	-0.68	-0.94	0.12	0.52	0.90	-0.23	-1.26	2.38	-0.15	-0.95	-1.92	-0.13	-0.53
Hspa1b	0.57	-0.12	0.42	1.19	1.29	-1.72	0.19	-1.29	-0.89	0.14	0.21	0.54	-0.46	0.81	-0.55	1.62	0.48	-0.02	1.71	0.81
Gm8273	-0.49	1.32	0.35	0.13	0.87	-0.61	-1.59	-1.76	-0.19	-0.57	0.22	0.92	-0.65	0.09	-0.09	-0.56	0.37	0.17	0.15	-0.22
Bag1	0.11	1.25	-0.04	1.18	0.61	-1.61	-0.49	-0.25	-0.68	-0.63	-1.28	0.65	-0.84	-0.65	0.83	3.23	1.05	-0.06	0.98	0.43
Cetn2	-0.78	0.09	1.07	0.45	-0.13	-1.16	-0.81	-1.78	-1.75	-0.58	0.23	0.90	0.32	1.19	0.09	0.84	0.75	-1.27	-0.13	0.48
Hint1	0.49	-0.18	0.32	-0.13	0.12	-2.84	-0.31	-0.36	-2.20	-0.40	0.12	1.52	-0.49	1.73	0.69	1.27	-0.36	-1.72	0.49	0.65
Pom121	0.26	-0.57	1.28	0.66	0.47	-0.78	-2.16	-0.19	-1.95	0.49	0.77	-0.47	-0.07	-0.92	1.67	0.31	0.08	0.47	0.85	-1.58
Nhlrc2	-0.19	0.11	0.68	-0.11	-1.31	-2.56	-0.38	-0.61	-3.18	-0.75	0.80	-0.76	-0.67	0.49	-0.50	1.33	0.19	0.59	0.77	-0.34
Lcn8	0.89	0.56	0.56	-0.35	0.59	0.09	0.03	-1.32	0.07	-3.27	0.45	-0.16	-0.14	0.55	-0.09	0.34	-0.76	-0.73	-0.25	0.60
Hspa1a	-0.21	-0.92	0.43	1.19	1.47	-1.88	-0.85	-1.47	-0.91	0.41	0.59	0.59	0.08	0.42	-0.54	1.38	0.35	1.48	1.84	0.86
Ccdc43	-0.55	0.37	-0.46	0.27	2.32	-1.75	0.34	-1.25	-0.41	-1.59	-1.13	0.75	1.51	1.60	0.38	0.05	-0.05	-0.48	0.40	-0.31
Cyp26b1	0.41	0.81	0.70	-0.02	0.44	-1.18	-1.01	-1.32	0.57	-1.29	1.87	0.03	0.19	-0.27	0.34	-1.06	0.44	-1.37	0.22	1.31
Socs4	0.08	0.00	0.32	0.27	0.51	-2.01	-0.40	-2.94	-0.08	0.01	0.42	-3.93	0.97	0.50	0.22	0.29	0.56	0.40	0.88	0.41
Ebf3	-0.03	0.82	0.40	-0.18	0.80	0.53	-2.13	-2.19	0.44	-1.37	0.98	-0.02	0.76	0.64	0.44	0.14	-0.37	-1.12	1.08	1.05
Faap20	0.19	0.21	1.07	0.17	0.48	-1.13	0.03	-0.25	-1.62	-1.40	1.52	-0.91	-0.03	-0.68	-0.40	0.12	-1.41	-0.83	0.36	0.80
Idnk	1.01	0.27	1.29	-0.52	-0.03	-1.77	-0.08	0.16	-1.41	-1.35	-0.15	-0.20	0.67	1.62	0.95	0.68	-1.05	-1.38	1.27	0.43
Msl3	0.55	0.90	1.12	0.70	0.94	-2.31	-0.35	0.84	-0.36	-0.04	-0.46	0.29	1.03	0.74	0.07	0.83	0.48	-1.06	0.93	-0.15
Tomm7	0.51	0.69	0.75	0.23	-0.80	-1.51	-0.33	-1.71	-0.61	-0.87	-0.48	0.46	-0.74	0.20	0.62	1.26	0.50	0.20	-0.55	0.86
Yae1d1	-0.03	0.34	-1.21	0.46	-0.22	-1.15	-2.39	-0.62	-1.66	-1.25	0.41	-2.89	1.37	1.62	0.98	-0.06	-0.28	0.41	-0.18	-0.19
Ccdc73	-0.24	1.13	-0.52	-0.02	0.48	0.31	-3.45	-0.91	-1.05	-0.48	0.39	-0.05	0.05	0.41	1.18	-1.07	0.64	-1.26	0.81	-0.31
Zfp958	-0.24	0.62	0.11	0.71	0.43	0.26	0.48	-2.07	-0.98	-2.39	0.59	-0.05	0.90	0.99	0.85	2.29	0.09	-0.36	1.35	-0.72
Hspe1	0.84	0.72	1.15	0.63	0.45	-0.10	0.23	0.23	-2.05	-0.85	-0.77	0.19	-0.67	-0.22	-0.28	1.03	1.05	-0.88	1.02	2.14
Pik3cg	1.23	-0.20	-0.30	0.11	-1.63	-1.12	-2.38	-2.11	-0.67	-0.80	-0.44	0.16	-0.19	-1.08	-0.09	-0.24	0.01	-0.66	1.52	1.47
Ccdc191	0.05	0.87	0.48	-0.68	-0.71	-1.97	-2.54	-0.36	0.34	-1.72	-0.27	-0.47	0.48	0.46	-0.49	0.94	-1.96	0.12	-0.21	-1.72
Ythdc1	0.01	-0.36	-0.46	1.89	1.22	-1.07	-0.34	-0.53	-1.19	-0.80	-1.07	-2.22	1.66	0.53	0.80	0.08	0.09	1.09	0.48	1.03
Rpl29	1.58	1.34	-1.16	0.73	0.84	-1.16	0.35	0.34	-0.63	-1.65	-1.11	0.63	0.40	0.70	0.85	0.02	-0.23	0.79	0.87	1.31
Zwint	-0.62	-0.25	0.06	0.60	0.66	-1.07	-0.80	0.29	-0.69	-3.34	-2.01	0.86	0.61	0.40	0.64	0.75	0.70	0.68	0.33	0.46
Pigu	0.45	1.10	0.82	1.06	1.31	1.29	0.16	0.12	-1.38	-1.52	-1.10	1.58	0.45	0.25	0.38	0.37	0.74	0.27	0.56	1.13
Smad1	0.57	0.50	-0.29	0.02	-0.09	0.28	-1.86	-1.86	-1.03	-0.81	-1.35	0.82	-0.61	-1.83	0.58	0.42	0.52	0.54	1.88	1.92
Rint1	0.37	0.31	0.15	-0.77	0.74	-2.09	-0.64	-0.97	-0.96	-0.49	1.22	-0.59	1.14	-0.01	0.44	-1.31	-1.22	-0.30	0.36	0.57
Fam58b	0.64	0.23	0.76	-0.36	1.45	0.44	-1.04	-1.73	-0.77	-0.10	0.21	1.13	1.06	0.47	0.61	-0.10	0.77	-0.69	-0.08	1.15
Oma1	1.62	-0.34	0.02	0.71	0.27	-1.40	-0.56	-0.69	0.16	-1.11	0.52	-0.05	0.52	0.04	-0.19	0.43	-0.85	1.58	0.30	0.40
Cdkn2d	-0.64	0.64	0.59	0.82	0.88	-1.58	1.23	-1.16	-1.55	-0.52	-0.08	0.15	0.58	1.06	-0.43	-0.91	1.58	0.24	-0.35	0.61
Scamp2	0.02	-0.52	-0.12	-1.11	-0.63	-2.76	-2.00	-1.20	-1.13	-1.06	0.24	-0.77	-0.30	-0.34	0.97	0.91	-0.29	-0.22	1.55	1.93
Rrm1	0.04	-0.53	-0.26	-1.20	1.11	-0.50	-1.24	-1.52	-2.17	-1.18	-0.43	0.13	-0.31	0.57	-1.26	0.54	-0.88	0.34	-0.82	0.83
Rab37	-0.53	1.12	1.19	0.67	0.78	0.45	0.01	-0.37	-1.15	-1.48	-0.23	0.65	-0.36	0.08	0.74	0.90	-0.88	0.63	0.39	1.19
Acadm	-0.79	-0.19	0.66	1.06	0.24	-1.16	-0.52	0.27	-2.41	-0.91	-0.88	-1.49	0.18	1.38	0.12	-0.63	0.61	0.48	2.29	1.06
Sox21	-0.37	1.28	0.11	1.39	0.12	-1.14	-0.44	0.30	-0.83	-1.05	-0.42	0.53	0.37	0.45	0.12	0.88	1.69	0.91	1.47	0.75
Zfhx3	0.81	-0.16	0.28	0.56	0.80	-2.84	0.09	0.22	-0.18	-0.66	0.51	0.40	-0.63	0.53	-0.30	-0.04	-0.16	0.17	0.78	1.07
Ctsc	1.47	1.30	0.14	-0.20	1.29	-0.06	0.23	-1.91	0.08	0.00	-0.17	0.59	0.96	-0.59	0.77	0.52	-0.74	-0.43	0.31	1.07
Tmem23	-0.93	-1.25	0.74	0.26	0.77	-1.27	-0.34	-0.66	-1.21	-2.55	0.76	-1.51	0.18	0.45	1.06	0.57	0.02	-0.34	0.54	0.05
Trp53inp	0.35	0.13	0.33	-0.08	-0.44	-0.88	0.18	-0.82	-2.83	-1.00	0.65	-0.27	0.84	0.77	0.84	0.87	0.02	0.62	0.38	0.76
Creld2	-0.13	0.28	1.37	-0.45	0.08	-1.35	-1.60	0.08	-0.40	-1.09	-1.88	-0.94	-0.67	-0.38	1.37	-0.03	1.09	0.49	0.84	0.30
Sh2b3	0.81	0.62	-0.35	0.60	0.58	-0.41	0.82	-0.29	-2.49	-0.89	-1.03	0.39	0.67	0.90	0.68	0.50	-1.59	1.03	0.65	-0.47
Letm2	0.05	0.38	-1.28	0.03	0.66	-0.70	-0.39	-2.42	-0.95	-1.14	-0.04	0.17	-0.98	1.22	2.61	-0.45	0.60	-1.99	0.63	-0.91
Gpat4	-1.22	0.42	-0.74	0.69	0.15	-1.50	-1.57	-0.49	-2.24	-0.31	-0.35	-1.06	-0.56	1.63	-0.20	1.70	1.05	-0.76	-0.16	0.57
F9	0.66	-0.45	0.02	0.85	0.41	-1.05	0.57	-0.53	-1.32	-1.57	0.67	-1.48	0.61	0.29	0.32	0.81	0.54	0.89	0.90	0.99
Klf9	0.31	-0.60	-0.46	0.29	0.09	-1.00	0.13	-0.77	-2.86	-1.25	-0.73	0.93	0.43	1.37	-0.73	0.16	-0.41	-0.60	-1.08	-0.44
Sntg1	0.47	0.99	0.44	0.58	0.73	-0.42	-0.33	0.25	-2.29	0.62	-0.11	0.17	0.79	0.31	0.61	0.46	0.94	-0.39	0.93	-0.60
Abhd5	0.21	0.14	0.64	-0.14	1.44	-0.89	-0.04	-1.44	-1.41	0.70	-1.26	1.70	0.67	-1.92	0.29	0.76	-0.10	-0.37	0.85	0.54

Golm1	0.21	-0.15	0.57	0.34	1.06	-1.24	-0.83	-0.53	-0.37	-0.37	-0.41	1.34	-0.62	-0.22	-0.80	0.17	-0.11	-0.52	1.79	1.27
Sec61b	-0.58	0.69	-0.16	0.84	-0.50	-0.89	0.07	-1.13	-2.09	-0.99	-0.73	0.26	0.59	-0.54	-0.10	1.32	1.14	0.84	1.39	1.69
Fuca1	-0.89	0.59	0.46	0.52	0.17	-1.36	-1.05	0.03	-0.65	-1.42	0.35	0.74	0.29	-0.51	-1.07	1.51	0.78	0.46	2.55	0.61
Pdss1	0.62	0.07	1.14	0.48	-0.77	0.34	-1.34	-0.41	-0.19	-2.16	0.90	-0.75	-1.21	1.30	0.04	-0.24	-0.97	1.10	-0.91	0.85
Zswim7	0.36	0.66	-0.28	1.44	-0.02	-1.00	-0.98	-0.30	-0.76	-0.05	1.45	0.38	-1.05	0.17	2.15	0.83	-0.64	-0.07	-0.30	-0.26
Diaph3	0.04	1.02	0.02	0.46	0.94	-1.59	0.45	-0.78	-0.67	-0.14	0.65	-0.61	-0.52	0.79	-0.09	0.15	0.41	0.44	1.15	0.65
Chd8	-0.07	0.12	0.48	1.67	0.31	-0.38	-0.35	-1.30	-1.18	0.55	0.55	-1.65	1.26	-0.26	1.37	-1.57	1.15	0.25	0.81	0.40
Pigr	0.46	0.13	-0.50	1.29	0.30	0.21	-0.11	-1.61	-1.74	-0.24	0.69	0.15	0.02	-0.60	0.24	0.65	1.51	-2.24	0.45	-1.27
Aven	-0.39	0.31	0.85	1.24	0.29	-0.93	0.09	-0.27	-0.83	-0.90	0.71	0.26	-0.56	1.70	1.21	0.79	-0.21	0.80	0.20	-0.13
Mrrf	0.75	-0.26	-0.50	0.19	1.40	-0.02	-0.41	-0.75	-0.98	-1.35	-0.95	-0.72	1.11	0.16	2.06	-0.84	-0.39	-1.26	0.55	-0.69
Zfp110	-0.68	0.95	1.13	1.41	0.37	-0.95	-0.83	-0.13	-0.72	0.75	0.78	0.60	1.92	0.52	1.14	2.20	-1.66	0.62	0.91	0.08
Dubr	1.41	-0.57	0.92	0.37	0.33	-0.89	-1.37	-0.75	0.56	-0.11	0.41	-0.26	0.07	1.14	-1.26	0.34	-0.72	-0.01	0.41	0.92
Tial1	-0.88	0.30	0.75	-0.29	0.71	-1.30	-0.44	-1.14	-1.08	-0.46	0.03	-0.18	-0.95	1.62	2.00	0.31	1.20	-0.15	0.39	0.78
Lpar6	0.61	-0.08	-0.55	-0.72	-0.36	-2.08	-0.45	-0.35	-2.02	-1.19	-0.52	0.29	0.39	-1.41	0.52	0.07	0.17	-0.76	-0.17	0.32
Mageh1	-0.42	1.03	0.25	0.07	-0.55	-0.95	-0.42	-0.04	-1.41	-1.69	0.27	0.43	1.31	-0.70	0.86	-0.65	0.41	-2.22	-0.09	1.13
Pinx1	0.22	-0.05	0.08	0.81	1.26	0.02	-0.98	-1.33	-0.72	0.44	0.26	-0.55	0.42	1.36	1.31	0.32	0.75	-0.99	0.46	-0.11
Slc25a17	-0.55	0.49	1.52	-0.28	-0.13	-1.02	-0.82	-0.58	-0.33	-1.07	0.56	-0.73	0.92	0.49	-1.28	0.97	1.09	-1.24	-0.66	0.09
Osgep	-0.10	0.44	0.08	0.23	-0.57	-0.60	0.09	-2.08	-0.93	-1.23	0.37	0.93	-0.70	0.41	0.71	0.06	0.23	-1.29	0.27	1.63
Mok	0.67	-1.04	-0.25	0.66	1.41	-1.03	-0.65	-0.03	-1.04	-0.56	0.18	1.88	-0.59	0.66	0.97	-1.50	0.55	-0.17	0.48	0.62
Ttc32	0.33	1.76	0.55	0.55	-0.05	-0.87	-0.55	-0.29	0.30	-0.18	-0.36	1.37	-0.07	0.98	-0.82	0.16	-1.55	-0.19	1.66	1.22
Cacna2d	0.50	0.78	0.52	0.61	0.30	-0.96	0.61	-1.10	-1.00	0.46	1.60	-0.04	0.48	-0.59	-0.57	-1.10	0.14	0.64	0.74	1.19
Ahsa2	-0.33	0.88	0.99	-0.61	0.86	-0.49	-0.92	-0.23	-1.43	0.18	-0.25	0.62	-0.10	0.66	-0.08	0.16	0.93	-1.42	0.49	0.79
D10Wsu	0.61	-0.01	-0.84	-0.33	0.31	-1.16	0.20	-1.60	-1.19	-1.15	1.06	-2.55	0.01	-0.13	0.33	-0.05	1.17	0.09	0.55	-2.60
Rpl10	-0.04	-0.21	0.61	0.00	-1.15	-1.02	-1.02	-1.36	-0.48	-1.36	-0.78	-0.35	-0.29	-0.11	-0.04	1.32	-0.13	-0.13	1.94	2.76
Cnn3	0.18	0.16	0.46	0.91	1.25	-0.89	0.15	-0.34	-0.07	-0.31	0.35	-0.93	0.34	0.11	0.61	0.57	-0.02	-0.97	2.02	2.24
Gm6607	-0.01	-0.10	0.32	0.51	0.88	-0.07	-0.24	0.01	-1.20	-1.27	0.25	0.71	0.74	0.73	-0.23	-0.13	-0.54	0.04	0.23	-0.44
Aph1b	0.40	-0.09	0.69	0.62	-0.57	-0.30	-0.56	-0.05	-1.10	-1.23	-3.07	0.95	0.58	-0.01	0.73	-0.25	0.99	-0.08	0.64	-1.43
Oraov1	-0.10	-1.01	-0.70	0.41	0.58	-1.21	-0.76	-0.92	-0.91	-1.29	-0.60	1.38	0.08	-1.04	-0.61	0.76	-0.22	-0.27	0.51	2.55
Birc3	0.31	0.57	0.52	0.39	0.28	-0.33	-0.46	-0.42	-0.31	-0.70	-0.06	-0.20	-0.97	-0.24	-0.14	0.43	0.53	0.45	2.36	1.32
Fam107a	0.79	-0.74	0.02	0.47	-0.15	-1.11	-0.42	-0.58	-0.36	-1.40	-0.66	0.61	0.52	-0.50	-0.56	-0.38	-0.96	-0.32	1.58	1.63
Frem2	0.95	-0.04	0.43	0.47	0.00	-1.38	-0.19	0.24	-0.79	-0.31	-0.20	-1.05	0.35	-1.40	0.70	1.26	1.41	-0.30	2.56	1.22
Tcf7l1	0.85	0.15	0.70	1.11	-0.01	-0.35	0.16	-1.03	-0.44	0.24	-0.36	-1.01	0.00	0.35	0.03	0.94	0.32	0.63	1.81	2.17
Rps3	1.32	0.89	-0.10	1.27	0.10	0.30	-0.16	-0.27	-0.53	0.01	1.04	0.52	-0.99	1.32	0.22	1.41	0.30	0.19	0.30	1.02
Parp14	-0.06	0.12	-0.03	0.34	0.13	-0.97	-1.81	0.00	-0.76	-0.07	-0.91	0.33	0.25	0.20	0.22	1.41	0.30	0.86	2.21	2.01
Pex11a	-0.16	0.29	-0.64	0.15	-0.40	-0.71	-0.09	-1.12	-1.93	-0.99	0.28	1.00	-0.88	-1.03	-1.55	0.09	-0.09	0.46	-1.29	1.06
Slc7a6os	0.36	0.82	0.66	0.03	1.16	0.02	-0.05	-0.71	-0.79	0.55	1.07	1.04	-0.97	0.21	0.36	0.98	0.00	1.15	1.32	0.35
Arl4c	-0.68	-0.48	-0.30	-0.48	0.07	-0.97	-1.18	-1.28	-1.69	-0.71	0.43	0.15	-0.18	0.25	-1.92	1.05	-0.19	-1.71	1.01	1.33
Hist1h2b	-0.28	0.12	-0.40	-0.22	-0.18	-0.92	-0.28	-0.80	-1.40	-0.74	-1.02	-0.33	-0.03	-0.98	-1.02	0.82	-0.05	-0.66	1.26	2.09
Fam26e	0.23	0.34	1.51	1.05	1.15	-0.72	-0.99	-1.21	-1.08	-1.41	1.57	0.78	1.29	0.27	-1.35	-0.56	0.34	-0.79	0.48	-2.57
Hspa5	0.79	0.50	1.05	1.52	1.18	-1.17	0.49	-1.22	-1.02	-1.30	-0.53	0.58	-0.22	0.02	-0.75	-0.42	-0.34	-1.10	-0.25	-1.43
Cdc25a	2.05	0.47	0.02	0.97	1.83	-0.40	-0.77	0.65	-0.75	-1.83	-0.15	0.36	0.96	0.38	-0.56	-1.14	0.39	-1.29	0.15	-1.67
Ppa1	1.12	1.15	1.18	1.64	1.13	-0.15	-1.90	0.61	0.47	-1.21	-1.08	0.39	0.14	0.87	0.17	0.82	-0.44	0.28	-0.20	-0.24
Foxd1	1.00	2.32	-0.07	1.48	1.57	-1.40	0.28	-0.31	0.33	-0.83	0.68	1.34	0.43	-0.18	0.22	-0.65	0.54	0.81	-1.45	-0.74
Htra1	0.86	0.75	1.41	0.79	1.60	-1.39	0.07	0.43	-0.86	-0.90	-1.40	1.26	-0.62	1.18	0.33	-0.89	-0.74	-1.08	-0.82	-0.55
Hsph1	1.41	-0.36	1.50	1.88	1.67	-1.07	0.60	0.51	-1.87	0.08	-0.41	0.87	0.31	0.76	-0.51	0.95	-0.61	-0.42	-0.29	-0.13
Kcnb1	0.24	-1.27	-0.94	-0.41	-0.40	-0.16	-1.20	0.06	0.24	0.20	0.22	0.53	-0.61	0.51	1.06	-1.06	0.32	0.73	-1.54	-2.32
Wdr60	0.25	0.49	0.38	0.86	0.65	0.44	-1.51	-0.60	-2.34	-0.93	-0.98	0.89	0.10	-0.68	0.30	-0.63	-1.18	0.96	-0.66	-0.53
Ap3s2	0.78	0.57	0.56	0.54	0.43	0.68	-2.56	-3.28	0.37	0.18	0.86	0.49	-0.15	-0.29	-0.07	-0.08	-3.08	0.88	-0.52	-0.37
Rpusd2	0.82	0.58	0.88	0.71	0.80	-2.09	-1.19	0.33	-0.76	0.02	0.35	1.10	-0.40	0.95	-0.57	0.58	0.43	0.16	-1.20	-0.70
Gopc	1.42	0.99	0.91	0.97	0.81	0.31	0.42	-0.22	-2.76	0.03	0.05	1.41	0.02	0.29	0.57	-1.11	1.66	0.22	0.16	-2.34
Hnrnpm	-0.12	1.15	2.31	1.26	2.21	-0.16	1.21	1.26	-1.68	-1.10	0.27	-0.39	0.79	-0.71	-0.60	-1.39	-1.19	-0.34	-1.56	-0.15
Galnt11	1.42	0.31	2.17	-0.45	0.94	-1.20	-0.68	0.05	-0.64	-0.41	0.29	-0.25	0.05	2.29	-0.24	-0.85	0.50	1.55	-0.86	0.53
Gm3740	1.10	1.56	-0.25	0.84	0.99	0.64	-0.43	-2.35	-0.47	-0.37	0.77	0.16	0.19	-1.01	-0.44	-0.44	0.44	0.84	-0.22	-0.76
Suv39h1	1.62	0.88	0.41	0.29	0.51	0.02	-0.42	-3.24	0.04	0.19	-0.33	-0.64	0.43	-1.23	0.76	-0.91	0.34	-0.39	0.23	-1.21
Mrpl44	0.96	1.64	1.52	0.51	-0.32	-1.17	-0.48	0.65	-0.84	-0.89	-0.29	1.44	-1.52	-1.42	0.91	1.34	0.56	0.72	-0.66	0.02
Cys1	1.48	-0.92	0.33	1.55	0.95	-0.86	-0.41	0.72	-2.20	-0.87	-0.12	-0.19	0.07	0.20	0.00	-0.17	0.68	-0.63	-0.06	-2.17
Mtg2	2.23	1.03	0.93	1.12	2.24	-0.81	0.01	0.45	0.52	0.37	-0.99	0.02	-2.12	-0.77	0.55	-0.75	-0.43	0.41	-1.52	-0.19
Sft2d3	1.43	1.61	2.30	-0.10	0.28	0.02	-0.75	-0.48	0.01	-0.17	-1.60	1.01	0.37	-0.10	-1.55	-0.37	-0.64	0.12	-1.03	0.03
Gm4926	0.92	1.55	0.10	-0.32	0.79	-1.95	-1.76	0.97	-0.38	-0.69	-0.08	0.28	0.09	0.34	0.74	0.01	1.32	0.10	-1.89	-1.17
Wdr74	0.90	1.81	1.58	0.04	0.41	1.06	-1.97	-0.04	-0.84	-0.18	-0.43	-0.30	-0.31	-0.80	0.32	0.52	0.88	1.23	-1.37	0.20
Sycp2l	0.67	0.72	0.70	0.93	0.98	0.76	-1.97	0.69	0.06	-2.19	0.71	0.82	-0.84	0.40	-0.45	0.55	0.42	0.53	-0.60	-1.01
Slc35c2	1.93	1.74	1.72	1.93	0.77	-1.36	1.87	1.05	-0.39	0.31	-0.96	0.68	0.45	-0.65	-0.67	-0.13	0.12	0.16	-1.77	-1.04
Cx3cl1	0.18	0.50	1.72	1.68	-0.20	-1.16	-1.24	0.75	-1.33	0.33	0.09	0.23	0.31	-0.55	-0.43	-0.18	0.06	0.42	-2.94	-0.57
AU02102	2.19	-0.40	1.30	1.47	1.64	0.07	-0.92	0.54	-1.00	0.99	1.24	0.21	-1.15	-0.42	0.27	-0.56	0.74	-0.56	-0.47	-1.40
Inpp4a	1.39	1.18	0.33	0.72	0.84	0.56	-0.88	0.92	-2.29	-0.31	1.05	-1.01	-0.11	0.56	-1.18	0.53	-0.34	0.88	-0.68	-0.10
Bcr	0.55	1.06	2.02	0.54	2.19	-0.36	0.66	0.12	-0.35	-0.15	0.38	-0.50	-0.98	0.58	-0.79	-1.85	-0.84	0.18	-1.33	-2.58
Mgat4b	2.06	2.48	1.09	1.12	0.98	0.66	-0.36	-0.53	1.27	0.30	0.47	-0.39	0.35	-1.40	-0.32	-1.33	0.14	0.71	-2.15	-0.44
Neto2	0.78	0.50	0.64	0.09	-0.28	-2.35	0.76	-2.06	-1.13	0.16	0.35	0.52	-0.19	0.85	0.58	0.70	-0.59	-0.37	0.14	-3.93
Gm3499	1.74	0.95	1.33	0.51	1.28	0.24	0.69	-0.63	-0.42	-0.34	0.22	-0.13	-0.99	-0.19	0.12	0.91	0.07	-0.58	-1.36	-4.36
Vwa9	1.89	1.26	1.55	1.24	0.69	-0.65	1.41	0.53	-1.00	0.08	-1.40	-0.37	0.26	0.18	-0.65	-1.05	-1.08	-0.76	-1.97	-1.53
Rpn1	1.00	0.80	1.22	0.90	1.10	-0.83	0.49	0.28	-1.11	0.05	-0.23	0.99	-0.58	0.40	-0.66	0.22	0.25	0.01	-0.51	-0.11
Zfp3	0.84	1.96	1.63	1.74	0.66	0.77	0.33	-0.23	-0.17	0.03	0.15	-0.59	0.65	-0.05	0.25	-0.23	1.15	-0.84	-0.69	-1.47
Naif1	1.70	0.79	0.57	1.00	0.32	0.19	-2.16	0.01	0.28	0.01	1.11	0.60	-1.39	1.33	-1.03	0.26	-1.20	-0.06	-0.90	-1.81
Shh	2.07	0.28	1.01	0.65	1.62	1.02	-0.17	0.50	-1.07	-0.63	-1.64	-1.08	0.35	0.88	0.36	-2.61	-0.25	1.11	-1.05	0.76
Pygo1	0.78	1.77	0.13	0.79	0.53	-0.73	-0.25	-1.67	0.82	-0.04	0.17	-1.34	1.43	-0.52	-0.09	0.31	0.93	0.89	0.79	-1.28
Gzf1	0.43	-0.02	2.39	0.37	1.37	-0.61	0.47	0.18	-0.23	-1.14	0.89	0.64	0.91	0.60	0.22	1.79	1.03	-0.63	-1.18	-1.89
Vdac3	0.51	0.75	0.44	1.81	1.19	0.02	-0.05	0.31	-1.61	0.17	-0.66	0.76	1.27	0.98	1.50	0.54	0.54	0.19	0.35	-1.43
Eftud2	1.28	0.09	0.80	-0.01	0.10	-1.46	-0.12	-0.36	-0.81	-0.84	-0.49	-0.99	0.32	1.18	-0.90	-1.73	0.25	0.46	-1.00	-1.66
Clec11a	1.45	1.23	1.28	1.04	1.31	0.86	-1.52	-0.05	0.39	0.81	0.59	0.51	0.84	0.35	-1.69	0.29	1.05	-0.32	-0.30	-1.57
Mast1	1.06	1.67	0.21	0.84	0.82	0.21	-0.53	-0.70	-0.27	0.07	0.76	-1.55	-1.30	-1.38	0.59	-1.23	-0.54	-0.08	0.02	-1.46
Mrps35	1.27	0.41	0.40	0.50	0.14	-0.76	-1.59	0.40	-0.70	-0.36	-0.45	0.80	-0.33	0.65	-0.22	0.11	-1.52	-1.24	-0.78	-0.53
Ypel1	0.67	-0.05	0.40	0.94	0.04	0.00	-0.99	-2.01	0.58	-1.31	0.45	0.54	0.29	0.78	0.07	-0.49	-3.11	1.38	-0.63	-0.75
Emc8	1.85	0.58	1.17	0.51	1.26	0.76	-1.45	-0.62	0.54	0.43	0.27	0.39	-0.61	0.18	-0.74	0.16	-1.83	1.01	1.04	-0.64

Kif26b	0.90	-0.18	1.22	0.64	1.22	0.31	-2.08	0.58	-0.04	-0.65	-0.29	1.26	-0.82	-1.12	0.94	-1.64	0.74	-0.30	-1.51	-1.04
Magef1	0.90	0.05	1.50	1.66	0.33	-0.62	1.15	0.16	-1.00	-0.81	-1.50	-1.82	0.95	-1.01	0.30	0.37	1.12	0.56	0.15	-2.06
Tmem16	1.42	0.52	1.23	0.94	-0.61	0.25	-0.58	0.21	-1.23	-0.66	-1.52	0.55	-0.63	0.73	-0.65	-1.47	-0.16	-0.51	-1.75	0.22
Endog	0.94	0.68	1.33	1.71	1.77	-0.06	0.38	0.58	-0.79	0.89	-0.42	0.29	0.97	0.22	-1.42	0.11	-0.81	-0.25	-0.27	-0.80
Dusp14	1.62	1.84	1.69	1.92	1.74	1.11	1.00	0.61	0.43	0.24	-0.37	0.20	-1.48	-0.83	0.32	-0.01	-1.10	-0.46	-2.04	-1.14
Rasd1	1.17	0.85	0.84	1.38	0.46	-0.93	-0.27	0.15	-0.74	1.18	-0.11	-0.12	-0.96	0.15	-0.09	-1.73	0.11	0.66	-0.73	-2.39
Cyp4f15	1.75	-0.16	1.38	2.30	0.77	0.25	0.06	0.39	-0.20	0.28	1.42	1.25	0.36	-0.03	-0.91	-0.74	0.66	0.68	-1.39	-0.47
Dbn1	1.11	0.73	1.71	1.47	1.56	0.22	-0.64	0.49	0.75	0.52	0.25	-0.37	-0.33	0.13	-1.09	-2.19	0.10	-0.14	-0.97	-0.33
Rnf114	1.31	1.47	0.91	1.60	1.24	0.92	0.18	0.53	-0.54	0.21	1.65	0.79	0.55	0.53	-1.38	-0.63	-1.24	0.65	0.50	-0.58
I7Rn6	1.37	-0.33	0.14	1.09	0.56	0.14	-0.13	-1.11	-1.38	0.08	0.07	0.04	0.11	1.76	-0.44	-1.16	1.28	-0.30	-0.47	-0.53
D5Ertd57	1.16	1.28	-0.18	1.09	1.95	0.11	0.62	0.41	-1.46	0.42	0.09	-0.11	-1.28	0.36	1.62	-1.45	-0.54	1.69	0.05	0.18
Cdk5	1.87	0.81	1.56	1.30	1.19	0.37	1.47	0.21	-0.48	-0.02	-1.56	-0.24	-1.20	-0.21	0.53	-0.42	-1.44	-0.09	-0.12	-1.76
Syce2	1.50	1.36	1.04	0.72	0.55	-0.06	-0.14	1.05	-1.52	0.70	1.13	1.49	1.38	0.58	-1.25	0.65	0.17	0.06	0.65	0.27
Slc25a20	2.20	0.33	0.59	0.81	0.75	-0.80	0.08	0.34	0.02	-0.05	0.36	-0.41	-1.81	0.85	-0.46	-0.59	-0.27	-0.28	0.06	-1.50
Dpcd	1.74	0.86	-0.29	0.82	-0.59	-1.22	-0.79	-0.52	0.03	-0.03	0.15	0.20	0.63	0.42	0.46	-0.27	1.68	-0.83	-1.75	-1.35
Camk2b	1.76	1.29	2.13	1.56	1.48	0.86	0.89	1.64	-0.21	0.00	0.03	-0.67	0.38	0.37	-0.26	-1.67	-1.25	0.64	-1.36	-1.40
Trim17	1.26	0.27	0.53	0.67	0.22	1.64	0.81	1.14	0.31	-0.63	0.27	0.48	-1.81	-0.40	0.28	-0.59	0.21	0.70	-1.69	-2.39
Srl	1.36	1.39	1.25	1.24	1.00	0.07	0.02	-0.27	0.23	1.16	0.71	-0.04	-0.06	-0.83	-0.67	0.80	0.40	0.24	-1.02	-1.43
Dph1	1.48	2.37	1.97	1.07	0.81	0.42	0.39	1.30	0.55	0.14	-0.86	-0.45	0.28	0.16	-0.04	-1.34	0.54	0.22	-1.37	-0.16
Vps45	2.07	1.42	1.33	0.40	0.41	-0.08	-0.56	-0.10	0.93	0.56	0.95	-0.91	-1.89	1.18	-0.78	0.10	1.30	0.10	-0.27	-1.17
Ist1	1.27	1.41	2.18	1.79	1.41	0.85	0.48	0.50	0.87	0.51	-0.43	-0.76	-0.48	-1.09	0.42	0.49	-0.80	0.05	-0.19	-1.16
Fam162a	0.73	1.47	1.17	0.40	0.69	-1.26	0.03	-0.26	0.75	0.36	0.17	0.11	-0.63	0.55	-0.40	-0.37	0.79	-0.93	-0.73	-0.33
Pabpc1	-0.27	1.47	1.43	1.12	1.46	0.90	-0.42	0.15	-0.89	0.66	0.70	-0.06	0.16	-0.26	0.65	-0.11	0.43	0.01	-0.18	0.43
D17Wsu	2.20	1.35	1.06	1.99	0.99	0.09	1.17	1.08	0.52	-0.06	-0.69	-0.86	0.58	-0.58	-0.57	-1.30	0.88	0.03	-1.29	-0.55
Vps8	1.73	0.60	0.80	-0.49	0.02	-0.31	-0.80	-0.68	-0.85	0.53	1.61	0.32	0.25	0.35	1.56	1.65	0.40	0.76	-0.50	-2.45
Rps6kl1	0.79	1.11	0.93	0.87	1.19	-0.24	-0.83	0.47	0.99	-0.24	0.37	0.75	0.73	-0.67	-0.81	1.03	-1.87	-0.01	-2.18	-1.13
Zbtb40	1.85	0.29	0.23	1.61	1.52	-0.24	0.33	-0.45	0.04	1.09	-1.12	1.30	-0.45	-0.04	-0.23	-0.15	0.09	-1.52	-0.53	-2.61
Dnajc21	0.39	0.46	0.52	0.47	0.51	-1.69	-0.66	-0.85	0.62	0.21	0.48	1.24	0.28	-0.27	0.42	-0.45	0.69	0.38	-2.08	-2.27
Mef2b	0.81	0.21	0.36	0.95	0.48	-0.12	-0.63	-0.38	-0.10	-0.66	1.40	0.37	-1.66	0.59	-1.15	-0.38	-1.69	-0.70	-1.28	-0.91
Fn3k	1.16	0.29	0.42	0.42	0.12	-0.22	-1.60	0.20	-0.57	-0.09	0.06	1.51	-0.53	-0.07	0.01	-1.08	-0.31	-1.25	-1.26	0.10
Ier5	0.98	0.39	-0.07	0.97	1.19	-0.33	-0.64	1.16	-0.25	-1.13	0.60	-0.14	-1.36	0.92	1.03	-0.03	1.61	-1.44	-2.43	0.38
Gps2	1.71	1.25	1.02	1.10	0.44	-1.23	0.14	0.32	0.76	0.92	-0.93	-0.99	-0.27	0.46	-1.21	-0.13	0.06	-1.30	-3.76	0.19
Snrpn	1.64	1.96	1.34	1.56	1.64	1.17	1.35	1.08	0.70	-0.74	0.73	-0.17	0.25	-0.04	-1.35	0.14	0.36	-0.24	-0.11	-0.53
Pex5	0.56	0.55	0.89	0.13	0.65	0.63	0.05	-0.44	-1.49	-0.56	0.51	0.41	-1.26	0.10	0.12	-0.96	-0.70	0.83	-0.11	-0.99
Arf5	1.11	1.98	1.30	0.72	0.74	0.32	0.14	0.89	-0.72	0.64	-0.67	0.59	-0.66	-0.09	0.58	-0.10	-0.02	-0.48	-2.37	-2.40
Depdc5	0.55	0.31	0.41	0.31	0.49	0.22	-1.02	-1.25	0.22	-0.66	0.79	-0.69	0.37	0.92	0.16	-2.48	0.56	-0.56	-0.18	0.01
Nap1l4	0.72	0.81	0.38	1.24	2.53	0.54	0.17	-0.51	0.73	0.20	-0.06	-0.93	-1.69	0.22	0.24	-0.81	-0.28	1.14	-0.46	0.03
Psmb5	0.11	-0.09	0.80	0.11	-0.12	-0.95	-0.74	-0.15	-1.60	-0.27	0.41	0.89	-0.17	0.21	-0.61	-0.26	0.28	-0.03	-1.61	-1.98
Nt5m	2.03	1.45	0.89	1.81	0.95	0.12	0.19	1.03	0.01	1.30	-0.11	-0.25	-1.47	-0.83	-1.10	-0.50	-0.89	0.07	-0.83	-1.25
Plk2	1.74	1.07	1.76	1.70	0.87	0.66	-0.18	0.92	-0.12	1.40	-0.11	0.42	-0.07	0.55	0.53	0.17	-0.15	1.15	-0.24	-0.98
Srfbp1	1.41	0.47	0.84	0.62	-0.23	0.13	-0.30	-0.23	0.45	-1.29	-1.55	1.17	-0.58	1.61	0.29	0.54	-0.29	-0.94	-3.22	1.17
Mon2	1.37	0.94	1.09	0.98	0.10	0.16	0.17	-0.69	0.45	0.18	-0.64	-0.17	-1.13	-0.08	0.71	0.86	0.21	-0.52	-0.07	-1.71
Cntd1	1.46	1.07	1.01	0.40	0.54	0.31	-0.26	-0.35	-0.44	1.07	0.79	0.12	-2.71	0.29	0.43	1.65	0.34	0.06	-2.80	-1.03
Bank1	0.35	1.43	0.98	1.21	1.31	-0.64	0.58	0.26	0.34	0.61	1.11	1.27	0.64	-0.44	0.18	-0.62	1.11	0.09	-0.93	-2.12
Entpd4	1.46	1.61	1.16	1.76	1.21	0.62	0.68	1.18	0.35	0.25	-0.77	-0.15	0.02	-0.52	-0.68	-1.90	-0.27	-1.25	-0.58	-1.42
Pfdn2	0.36	0.55	0.42	0.50	0.31	-0.62	-0.23	-0.41	-0.90	0.26	-0.71	-0.61	-2.12	0.39	0.48	0.90	0.34	-0.60	-2.55	-1.95
Lrrc10b	1.29	1.04	1.93	1.49	0.90	-0.02	0.45	0.44	0.44	1.37	-1.73	0.35	-0.75	-0.34	-0.29	-0.81	-0.99	0.34	-2.81	0.25
Gm1033	1.50	0.54	1.48	1.47	2.06	0.60	0.77	0.54	-0.02	1.30	0.25	-2.05	0.64	-0.48	-1.12	-1.97	0.00	0.46	-0.16	-0.92
Fras1	0.61	0.72	0.97	0.51	0.50	-0.41	0.05	-0.21	-0.10	0.12	1.05	-1.33	1.19	-0.37	-0.34	-1.25	0.09	0.39	-1.13	-2.55
Dmap1	1.35	1.71	0.37	0.56	0.96	0.96	0.21	0.08	-0.05	0.00	0.67	-0.36	0.26	0.19	0.51	-0.20	1.14	-0.82	0.14	-0.49
Mrpl49	0.91	1.43	0.98	1.17	0.74	0.27	0.39	0.80	-0.26	0.64	-0.57	-1.30	0.50	0.73	0.20	-1.23	0.78	-1.95	-2.16	-0.69
Tbc1d17	1.17	0.76	1.34	1.50	1.31	0.93	0.25	0.40	0.52	0.67	-0.88	0.22	0.05	0.45	-0.11	0.31	0.18	0.49	-0.96	-1.32
Htra4	0.99	0.66	0.09	0.82	1.04	-0.09	0.00	-0.19	0.54	0.09	-0.56	0.12	-0.62	0.35	0.38	-0.30	-0.46	0.48	-0.28	-4.72
Pramef8	1.15	1.19	1.49	1.08	1.36	0.96	0.65	0.89	0.44	0.34	0.35	1.63	-0.59	0.13	-1.15	-0.15	-1.11	-0.61	-0.74	-3.20
C5ar1	0.26	0.71	0.61	-0.44	0.55	-3.45	-3.21	-0.33	0.57	-1.92	0.50	0.59	0.59	0.82	0.55	0.40	0.04	0.01	0.37	0.36
Smo	-0.39	0.05	0.31	0.35	0.33	-3.06	-3.22	-2.71	0.24	-0.57	0.67	0.76	-0.20	0.54	0.66	-1.18	0.56	0.55	0.49	-0.35
Tmsb4x	1.33	1.33	-0.35	-1.00	0.48	-1.92	-1.92	-1.92	-1.52	-0.35	0.48	1.33	-0.35	0.48	0.48	1.33	0.48	0.48	1.33	-0.35
Ift81	-0.46	0.52	0.88	-0.46	0.18	-0.12	-1.83	-4.33	-0.43	-0.67	0.72	0.43	0.09	0.20	0.97	0.48	-0.30	-0.39	0.14	0.16
Rbm27	0.26	0.68	0.58	-0.83	-0.70	-4.22	-0.48	-2.61	-0.24	-0.45	0.98	0.06	0.11	0.76	-0.02	0.27	0.86	0.24	0.64	0.48
Prkca	0.18	-0.32	-0.32	0.14	-1.15	-2.85	-1.28	-2.22	-2.58	-0.21	0.58	1.15	0.65	0.92	0.49	0.17	0.54	0.75	0.00	0.53
Ulk2	0.35	0.11	-0.51	-0.97	0.41	-1.64	-0.85	-1.82	-1.32	-2.50	-0.85	0.67	0.25	2.08	0.22	0.55	0.98	-1.02	-0.22	-0.24
Mob1a	0.83	-2.18	0.08	0.57	0.02	-1.41	-1.81	-1.73	-0.27	-2.97	-0.11	0.66	0.61	1.03	0.11	1.27	0.69	1.01	0.91	0.17
Ptbp3	0.32	-0.31	-0.76	-0.49	-0.13	-2.08	-3.30	-0.33	-2.32	-0.87	-0.53	-0.40	-0.04	0.42	0.62	0.79	0.42	0.74	1.05	0.38
Mettl13	-0.41	0.12	1.35	0.06	-1.14	-2.82	-2.11	-1.05	-1.01	-0.52	1.51	-0.46	1.09	0.95	-1.07	-0.52	0.37	0.54	-0.35	1.27
Mgrn1	-0.03	-0.42	0.58	-0.40	0.10	-2.78	-1.53	-1.03	-0.49	-1.72	0.26	0.22	-0.28	0.78	0.76	0.61	1.54	0.44	-1.10	1.09
Ralgps2	-0.43	-1.60	-0.26	-0.10	0.50	-1.46	-2.56	-1.06	-2.73	-1.39	0.71	0.80	0.50	0.20	1.22	-0.18	0.24	0.94	0.52	0.39
Galnt2	-0.31	0.32	0.17	-0.67	-0.21	-2.18	-0.97	-2.93	-0.43	-1.34	0.66	0.20	-0.82	0.28	-0.61	-0.21	1.36	0.51	0.68	-0.83
Cp	-0.56	0.40	-0.62	-0.55	-0.60	-0.65	-1.79	-1.45	-2.79	-2.37	-0.23	0.10	0.60	0.52	0.60	1.15	0.13	0.37	1.36	1.72
Cdc40	0.69	0.09	-0.05	-0.80	-0.92	-0.83	-1.70	-1.98	-1.82	-1.76	0.75	-0.46	-0.75	0.01	0.11	-1.79	0.45	0.41	-1.26	0.45
Tmem23	-0.13	0.45	-0.58	0.44	-1.38	-1.37	-1.87	-1.35	-1.36	-2.18	0.27	2.05	0.15	0.83	-0.78	-0.41	0.39	0.14	-0.27	1.17
Luc7l2	-1.03	-1.18	-0.63	0.31	-0.34	-1.96	-2.15	-1.75	-1.64	-2.10	0.59	0.06	1.79	0.62	0.38	-0.32	0.61	1.21	-0.19	0.81
Lysmd4	0.24	0.80	0.82	0.73	0.04	-2.28	-0.66	-1.83	0.27	0.60	0.78	1.38	0.18	0.60	0.38	0.54	-0.02	0.57	-0.66	-0.15
Rchy1	-0.04	-0.33	1.16	-0.70	-1.22	-1.26	-1.25	-1.81	-1.72	-1.61	1.14	0.33	0.48	1.01	0.38	0.25	0.88	2.38	0.68	-0.06
Ccdc77	0.87	-0.13	0.05	-1.42	-0.17	-1.73	-0.49	-0.32	-1.49	-3.18	0.19	0.32	0.04	0.42	1.09	0.36	0.48	0.16	0.91	1.21
Nup107	1.01	1.56	1.02	-0.36	-1.27	-1.07	-0.97	-0.13	-1.03	-1.16	1.01	0.53	0.13	0.29	1.09	-0.02	0.60	0.57	-0.19	0.84
Hba-a1	-0.32	0.32	0.32	0.32	-1.06	-3.17	-1.06	-0.82	-0.39	-1.24	1.09	1.08	1.72	0.30	0.16	-0.38	-0.86	-0.51	0.03	0.42
Hnrnpab	-1.13	-0.02	-0.89	-0.14	0.12	-1.10	-0.98	-1.09	-3.02	-2.08	0.87	1.81	-0.26	0.24	1.10	0.60	1.13	0.56	0.45	0.51
Nme7	-0.59	0.58	-0.21	-1.10	-0.28	-1.80	-1.92	-0.56	-2.73	-0.72	0.70	0.99	0.86	0.13	0.62	-0.93	0.71	0.38	-0.51	1.30
Mrps33	-0.26	0.06	-1.38	0.24	-0.03	-1.74	-0.52	-1.18	-2.38	-1.60	0.03	0.20	-0.43	1.87	0.52	-0.17	1.30	-0.48	0.48	0.37
Dync1li1	1.38	-0.04	0.19	-1.46	-0.65	-1.38	-1.53	-1.43	-0.87	-1.30	1.18	1.95	1.02	0.05	0.12	-0.87	0.86	0.19	-0.88	0.22
Cgrrf1	-1.12	-0.97	-0.18	0.01	0.18	-1.35	-1.14	-1.20	-1.70	-2.57	1.13	0.20	0.97	0.17	-0.38	0.82	0.42	-1.13	1.12	-0.42
Rtcb	0.16	0.51	1.55	-0.35	0.24	-1.39	-1.14	-2.12	0.21	0.70	1.00	0.84	1.32	0.35	0.87	1.89	0.20	1.49	0.40	-0.01
Zbtb5	-0.66	-0.78	-1.01	-0.66	-0.58	-1.92	-1.28	-2.05	-2.85	-1.43	-0.73	0.75	0.33	0.34	0.35	0.00	-0.38	-0.05	0.20	1.08

Ndufc1	0.21	0.25	-0.13	-0.34	-0.17	-2.40	-0.51	-1.99	-0.51	-0.56	-0.36	1.09	-0.65	0.41	0.66	1.07	0.99	-0.23	-0.48	0.43
Kcmf1	-0.55	0.11	-0.17	-1.07	-0.67	-1.84	-1.51	-0.83	-1.37	-2.57	0.54	1.07	-0.90	0.76	0.81	1.28	0.21	0.09	0.37	0.15
Pmpcb	0.08	-0.44	0.09	-0.53	-0.37	-1.94	-1.70	-1.84	-0.17	-1.18	1.35	1.01	-1.01	-0.30	1.10	0.06	0.02	-1.19	0.92	1.77
Csmd1	-0.36	-1.30	-0.39	-0.59	0.20	-1.20	-1.68	-3.04	-1.20	-0.96	0.53	0.41	0.10	0.68	0.47	1.08	-0.10	1.96	-1.91	0.44
Hbegf	-0.20	0.01	-0.32	-0.23	-0.94	-1.45	-0.93	-2.23	-1.16	-1.53	-0.08	0.68	-1.58	0.11	0.24	0.85	0.63	0.56	1.03	1.41
Mrpl9	-0.65	-0.40	-1.05	-0.59	0.20	-1.80	-1.51	-1.14	-1.55	-2.07	0.44	0.82	1.17	0.02	-0.15	0.75	1.15	0.44	1.48	1.01
Zfp365	0.40	-0.27	-0.67	-1.19	-0.55	-0.85	-1.21	-1.71	-1.76	-2.30	0.83	0.39	0.46	0.48	0.66	-0.02	0.55	0.36	-1.03	-2.52
Btaf1	0.78	-1.59	0.15	0.05	-0.99	-2.10	-1.96	-1.77	-0.19	-1.08	-0.76	-0.05	-1.21	0.10	1.33	0.78	0.39	0.64	1.01	0.23
Mospd2	0.68	-0.55	-0.04	-0.76	-0.82	-1.67	-0.72	-0.83	-1.28	-2.47	0.45	1.11	0.22	-0.24	0.57	1.14	0.94	1.00	0.71	-0.10
Hbb-bt	-0.76	-0.22	-0.48	-0.13	-1.18	-3.03	-1.03	-1.10	-1.31	-1.74	1.02	1.15	1.83	0.75	0.54	0.27	-0.12	-0.30	0.24	0.66
Srp9	-0.59	1.31	-0.60	-1.47	-1.04	-1.69	-1.49	-2.18	-0.52	-1.91	0.66	2.70	0.30	0.73	0.49	0.64	0.53	-0.24	-0.28	0.50
Zeb2	-0.43	-0.16	-0.95	-1.54	-0.89	-1.61	-2.33	-1.89	-1.27	-2.25	0.93	1.09	0.83	1.09	0.80	0.43	0.74	0.53	0.05	-0.64
Zcchc24	-0.25	0.09	0.07	-0.90	-0.70	-1.30	-0.59	-1.50	-1.76	-1.90	-0.08	0.10	0.09	1.60	-0.64	0.72	0.40	0.64	1.57	1.71
Ints8	-0.43	-0.88	-0.62	-0.88	-0.95	-2.06	-1.24	-2.01	-1.75	-2.04	0.75	0.80	1.05	0.63	0.81	0.94	0.96	0.68	1.03	0.34
Acp2	-0.42	0.33	-1.83	-0.17	-0.69	-0.87	-1.46	-1.57	-2.76	-1.38	1.48	0.46	-0.29	0.44	0.10	-0.28	0.11	0.20	-0.07	1.26
Atp6v1h	-0.47	-0.86	-0.80	-1.02	-1.07	-0.92	-2.27	-2.43	-1.89	-1.97	-0.18	0.52	0.65	1.25	0.30	0.23	-0.19	1.08	-0.06	-0.56
Ubxn2a	-0.29	-0.83	0.56	-1.78	-0.54	-2.06	-1.97	-2.36	-0.52	-1.11	0.27	0.77	0.78	1.17	0.68	0.72	1.14	0.90	-0.37	1.62
Arhgap3	-0.49	-0.46	-1.16	-1.20	-0.47	-1.58	-2.03	-2.45	-1.07	-1.79	0.07	0.40	0.37	1.05	0.87	-0.56	0.67	0.80	-0.85	-0.15
Luzp1	-0.78	-0.86	-0.91	-1.30	-0.60	-1.44	-1.73	-3.07	-1.36	-1.99	-0.01	1.17	-0.48	0.66	0.54	0.97	0.88	0.17	0.93	0.67
Ptprf	0.27	-0.05	-0.15	-0.92	-0.48	0.04	-1.91	-1.85	-2.08	-0.64	-0.33	-0.87	0.45	0.67	0.40	1.86	1.21	0.80	1.01	0.48
Mettl7a3	-1.31	-1.07	-0.25	-0.17	-0.31	-2.63	-1.56	-2.09	-0.62	-1.31	0.42	0.27	0.85	0.11	-0.80	-0.19	-0.08	0.65	0.50	1.26
Dhx36	-0.38	-0.52	-1.38	-1.41	-0.58	-1.51	-1.55	-2.05	-2.10	-2.03	0.66	0.36	0.92	0.67	0.52	-0.27	0.90	0.64	0.21	0.41
Polr3f	-0.17	-1.33	-0.10	-1.46	-0.18	-1.42	-1.57	-1.45	-2.20	-1.53	0.70	1.38	1.06	0.87	1.16	0.70	0.68	0.31	-0.12	0.20
H3f3a	-0.64	-0.89	-0.88	0.50	-0.94	-1.55	-0.67	-1.83	-2.51	-1.20	1.01	0.36	-0.17	0.58	1.46	0.08	-0.14	-0.02	1.36	0.67
Jarid2	0.95	-0.37	-1.17	-0.42	-0.68	-1.11	-0.86	-2.25	-1.49	-0.88	0.81	1.09	0.39	-0.72	0.32	-1.46	1.06	-0.91	-0.93	0.31
Elp4	0.67	-0.23	-0.05	0.79	-0.26	-1.22	-2.10	0.07	-0.38	-0.34	1.75	1.94	0.45	0.09	1.12	0.79	0.11	-1.00	0.43	0.15
Tsnax	-0.84	0.30	0.11	-0.23	0.39	-1.22	-0.44	-0.75	-2.07	-0.68	0.61	0.51	0.06	-0.53	0.66	0.41	0.57	1.30	-1.59	-1.05
Rpl9	0.16	-0.27	-0.82	-0.58	-0.35	-1.61	-1.54	-1.38	-1.02	-1.13	-0.35	0.47	-0.52	0.70	0.07	-1.02	-0.01	-0.44	-0.73	0.32
Insig2	-0.53	-1.11	-1.04	-1.49	-1.03	-2.14	-1.75	-2.12	-2.11	-1.90	0.48	0.83	0.32	1.09	0.54	0.38	0.80	0.51	-0.07	0.38
Psmd10	-0.43	-0.44	-0.85	-0.82	-1.24	-1.30	-2.35	-1.36	-1.40	-2.18	0.04	0.83	0.30	-0.29	-0.44	0.34	-0.43	0.74	0.65	0.19
Zcrb1	-0.70	0.00	-1.04	-0.87	-1.63	-1.59	-1.35	-1.75	-2.03	-2.35	-0.12	-0.18	0.34	0.38	0.88	0.69	1.22	0.60	0.33	-0.13
Endod1	-0.61	-0.78	-1.11	-0.99	-1.50	-2.15	-1.97	-1.64	-2.00	-2.00	0.35	0.63	1.09	0.70	0.34	0.67	0.75	0.66	0.57	0.98
Hyls1	0.81	0.33	-0.84	-0.53	0.11	-2.10	-0.31	-0.50	-1.18	-0.80	0.98	1.73	0.77	1.71	0.34	0.61	0.82	0.58	0.12	-0.73
Abhd15	0.52	-0.14	-0.26	-0.62	-0.11	-2.03	-1.26	-1.29	-1.12	0.35	1.59	0.39	0.63	-1.07	0.83	0.69	0.74	0.14	-1.89	0.20
Trpc5	-0.78	-0.63	-0.43	-0.57	-0.95	-1.52	-0.91	-1.27	-2.91	-1.50	1.11	0.94	1.03	1.16	0.52	-0.22	0.98	0.65	-0.40	-0.23
Rad21	-0.53	-1.52	-0.01	-0.66	-0.24	-2.20	-2.49	-0.40	-1.33	-1.24	0.08	0.05	0.15	-0.30	0.63	0.79	0.25	-0.79	0.63	1.38
Gad1	-0.01	0.42	-0.38	-0.85	-0.21	-0.46	-1.71	-1.17	-0.69	-1.64	1.64	2.28	0.83	0.30	0.65	0.00	0.97	0.51	-0.22	0.85
Galk2	0.48	-0.15	1.14	-0.25	-0.41	-0.60	-0.42	-0.49	-1.03	-1.29	-0.49	1.14	0.45	1.02	-0.83	0.77	1.01	0.54	0.94	0.69
Syt4	0.22	-0.38	-0.29	-1.12	-1.33	-1.35	-1.58	-1.47	-1.95	-1.18	1.04	1.42	0.30	0.41	0.26	0.65	0.70	0.20	-0.84	-1.10
Psmc1	-1.41	-1.15	-0.20	-0.65	-1.30	-2.31	-2.19	-1.76	-1.56	-1.46	0.42	0.87	1.40	0.63	0.51	0.99	0.17	0.68	-0.30	0.51
Cyld	0.55	0.14	-0.39	-0.15	-0.45	-1.96	-0.86	-1.35	0.11	-0.74	1.81	0.46	-0.23	0.16	0.30	0.24	0.96	-0.25	-0.36	-0.85
Gtf2h2	0.12	0.37	-0.18	-0.27	0.86	0.14	-0.28	-0.68	-1.09	-1.65	1.03	0.96	0.74	0.95	0.87	-1.98	1.62	0.19	-0.36	-0.09
Chchd6	0.30	0.36	-0.97	0.52	-0.16	-1.28	-0.87	0.23	-1.51	-0.94	0.58	1.67	2.28	-0.24	0.07	1.37	0.16	-0.44	-1.38	-0.60
Prpf38b	-0.81	-0.93	-1.09	-1.20	-0.67	-2.18	-1.72	-1.39	-1.71	-2.11	0.64	-0.83	0.57	0.54	0.57	-0.80	0.98	-0.35	0.50	0.35
Ankrd17	-0.64	-1.12	-1.05	-1.31	-1.09	-1.88	-1.77	-1.93	-1.96	-2.07	0.60	0.32	0.40	0.37	0.80	0.71	0.38	0.39	0.35	-0.11
Ints12	-0.52	-0.94	-1.31	-0.65	-0.19	-2.07	-1.35	-1.58	-1.92	-1.04	-0.16	0.77	0.75	-0.32	1.20	0.37	0.53	0.78	1.06	1.69
Aff4	-1.61	-1.66	-0.17	-1.20	-0.06	-1.76	-1.50	-1.55	-2.07	-2.04	-0.62	0.51	1.08	1.02	1.15	-0.02	0.73	1.12	0.19	0.09
Esyt2	-0.80	-0.93	-0.97	-0.84	-1.30	-1.73	-1.85	-1.92	-1.74	-1.80	0.74	0.62	1.20	0.38	0.54	0.80	0.37	0.25	0.69	0.09
Peli1	-0.97	-1.00	-1.34	-0.62	-1.23	-1.61	-1.32	-2.21	-1.87	-2.35	0.34	0.78	0.92	0.52	1.02	0.02	0.76	0.77	0.25	0.62
Nicn1	-0.05	-0.24	-0.31	-1.13	-0.46	-1.38	-1.12	-1.59	-0.75	-1.51	1.83	-0.39	1.22	-0.11	-0.48	-0.58	-0.03	0.56	-0.17	-0.05
Trappc6b	0.09	0.41	-0.45	0.04	-0.30	-0.30	-1.00	-1.10	-1.41	-0.58	0.77	1.26	-0.27	1.60	0.58	0.79	1.16	0.30	0.02	-0.27
Msantd4	0.05	-0.99	-1.14	-0.83	-0.26	-2.02	-1.09	-1.49	-2.07	-0.66	1.15	0.37	1.07	0.87	-0.01	1.02	0.31	-0.22	1.23	0.72
Slitrk2	-0.24	-0.37	-1.45	-0.96	-0.99	-0.97	-1.12	-1.50	-2.31	-2.26	1.17	1.39	0.02	0.98	0.63	0.15	0.52	0.78	1.08	-0.85
Rfwd2	-0.85	-1.43	-0.54	-1.16	-0.84	-1.98	-1.55	-1.97	-1.69	-1.75	0.37	1.52	0.07	0.82	0.99	0.81	0.33	-0.30	0.12	0.46
Kansl1	-0.88	-0.75	-0.90	-0.62	-0.67	-0.85	-2.27	-1.81	-1.67	-1.32	0.19	1.14	0.36	0.60	1.10	-0.22	1.16	1.11	0.89	1.07
Elf2	-1.31	-1.06	-1.29	-0.88	-0.74	-1.53	-2.22	-2.38	-1.54	-1.68	-0.05	0.70	0.40	1.20	0.70	0.38	0.99	0.41	0.11	1.07
Sp4	-0.19	-0.78	-1.29	-1.92	-0.86	-2.14	-1.96	-1.20	-1.57	-2.20	0.38	0.55	0.72	1.25	0.14	-0.29	0.56	0.33	-0.70	0.17
Homer1	-0.50	-1.07	-1.26	-1.15	-1.71	-1.63	-1.53	-1.41	-2.17	-1.69	0.17	1.10	0.59	0.83	0.80	-0.10	0.87	0.88	-0.31	-0.59
Ccnt2	-0.97	-0.80	-0.54	-1.42	-0.78	-1.58	-1.83	-1.84	-2.12	-1.12	0.43	-0.85	0.73	1.11	0.34	0.71	0.94	0.48	-0.25	0.55
Hsp90b1	-1.02	-1.08	-1.18	-1.23	-1.26	-1.59	-1.92	-2.18	-1.70	-2.31	0.50	0.61	0.26	0.75	0.47	0.54	0.56	0.31	0.43	0.39
Rnf20	-0.77	-0.70	-0.93	-2.36	-0.89	-1.79	-1.54	-2.22	-1.88	-2.11	0.22	0.76	0.57	0.75	0.77	0.22	0.64	0.76	0.28	-0.08
Baz2a	-0.61	-0.94	-1.17	-1.56	-0.84	-1.04	-1.93	-2.70	-1.68	-1.64	0.96	0.15	0.39	0.73	0.71	-0.19	0.96	0.85	0.18	-0.83
Magi3	-1.19	-1.08	-1.49	-1.08	-0.60	-1.54	-1.63	-2.34	-1.78	-1.99	0.49	0.40	0.79	1.03	0.65	0.52	0.64	0.30	0.36	-0.03
Pcmtd1	-1.12	-0.97	-1.30	-1.55	-1.00	-1.61	-1.85	-1.91	-2.36	-2.01	0.67	0.78	0.67	0.87	0.76	0.45	0.59	0.41	0.44	0.56
Hspa13	-0.78	-0.92	-1.41	-1.29	-1.22	-2.18	-1.90	-1.84	-1.65	-1.84	0.10	1.42	0.39	0.73	0.04	0.72	1.02	0.87	0.08	0.74
Psmc5	-0.51	0.03	0.26	-0.81	0.17	-1.21	-0.82	-0.44	-0.68	-1.43	-0.53	2.45	0.31	-0.39	1.48	0.30	1.20	-0.87	-0.79	-1.27
Nop10	0.22	-0.30	-0.82	-0.68	-0.70	-1.41	-0.93	-0.88	-1.59	-1.15	-0.14	1.08	-0.82	0.26	0.00	0.60	0.44	-0.74	0.13	0.72
Zc3h13	-1.00	-0.65	-1.35	-1.60	-1.18	-2.19	-1.96	-2.03	-1.28	-1.97	0.23	1.02	0.61	0.69	0.96	0.20	0.66	0.67	-0.15	-0.12
Gabpb2	-1.13	-1.37	-1.00	-1.15	-0.94	-2.35	-2.17	-1.87	-1.31	-1.50	0.52	0.35	0.71	0.38	1.25	0.49	0.91	1.13	-0.13	0.60
Zbtb38	-1.56	-0.83	-0.96	-1.31	-1.14	-1.87	-1.51	-1.86	-1.52	-2.39	0.08	1.18	0.46	0.62	0.63	0.63	0.84	0.51	0.30	-0.10
Dkk3	-0.62	-1.15	-1.27	-1.71	-0.91	-1.97	-1.93	-1.57	-1.48	-2.00	0.74	1.10	0.29	0.78	0.35	0.43	0.78	0.53	-0.52	-0.28
